# The origin and evolution of *ARGFX *homeobox loci in mammalian radiation

**DOI:** 10.1186/1471-2148-10-182

**Published:** 2010-06-17

**Authors:** Guang Li, Peter WH Holland

**Affiliations:** 1 Department of Zoology, University of Oxford, South Parks Road, Oxford, OX1 3PS, UK; 2 Key Laboratory of the Ministry of Education for Cell Biology and Tumor Cell Engineering, School of Life Sciences, Xiamen University, Xiamen, 361005, China

## Abstract

**Background:**

Many homeobox genes show remarkable conservation between divergent animal phyla. In contrast, the *ARGFX *(*Arginine-fifty homeobox*) homeobox locus was identified in the human genome but is not present in mouse or invertebrates. Here we ask when and how this locus originated and examine its pattern of molecular evolution.

**Results:**

Phylogenetic and phylogenomic analyses suggest that *ARGFX *originated by gene duplication from *Otx1, Otx2 *or *Crx *during early mammalian evolution, most likely on the stem lineage of the eutherians. *ARGFX *diverged extensively from its progenitor homeobox gene and its exons have been functional and subject to purifying selection through much of placental mammal radiation. Surprisingly, the coding region is disrupted in most mammalian genomes analysed, with human being the only mammal identified in which the full open reading frame is retained. Indeed, we describe a transcript from human testis that has the potential to encode the full deduced protein.

**Conclusions:**

The unusual pattern of evolution suggests that the *ARGFX *gene may encode a functional RNA or alternatively it may have 'flickered' between functional and non-functional states in the evolutionary history of mammals, particularly in the period when many mammalian lineages diverged within a relatively short time span.

## Background

The homeobox genes comprise a large and diverse gene superclass characterized by presence of a DNA motif encoding the homeodomain. Most homeodomain proteins function as transcription factors involved in switching other genes on or off during embryonic development, cell fate specification and cell differentiation. The pivotal importance of homeobox genes to animal development is demonstrated by the fact that mutation or experimental misexpression can cause dramatic developmental abnormalities or cancers [1-3]. The best known homeobox genes include *Hox*, *ParaHox*, *NK*, *Otx*, *Pax *and *Dlx *genes which have been extensively studied in many animal model systems including mice, zebrafish, nematodes and *Drosophila*. Indeed, it was comparison of homeobox genes between species that led to one of the most striking findings of twentieth century biology: the remarkable conservation of homeobox and other developmental patterning genes between very divergent animal phyla [4,5].

Not all homeobox genes are ancient, however, and the extent of their evolutionary conservation varies considerably. For example, a detailed search for all homeobox loci in the human genome sequence revealed six novel genes, *DPRX, ARGFX, TPRX1, DUXA, DUXB *and *LEUTX*, each of which has no orthologue in the mouse genome, nor in invertebrate genomes [6,7]. It was hypothesized that these homeobox loci originated relatively recently in evolution and had undergone rapid sequence evolution. Booth and Holland [6] suggested that *DPRX*, *TPRX1 *and *DUXA *may have originated by tandem duplication and extensive sequence divergence from the *CRX *homeobox gene (a member of the ancient and conserved *Otx *gene family), because *TPRX1 *is adjacent to *CRX *and the other two homeobox genes are just 5.8 Mb and 9.2 Mb distant in chromosomal region 19q13. The evolution of *DUXA *and *DUXB *was studied by Clapp et al [8] who showed that these genes originated before mammalian radiation but have been lost from mouse. In contrast, the origin and subsequent evolution of the *ARGFX *locus remains very unclear. Human *ARGFX *maps to 3q13 so is not in the same chromosomal region as *CRX*, or indeed any other Paired (PRD) class homeobox gene. The sequence of its homeodomain assigns *ARGFX *as a divergent member of the PRD class [6], but gives no clear solution to its mode of origin. In addition, it is not yet certain whether *ARGFX *is a true functional gene or a nonfunctional pseudogene. The existence of two human retrotransposed pseudogenes derived from *ARGFX*, three ESTs from human testis tumor and a weakly positive RT-PCR amplification from human testis and embryonic stem cells indicates a low level of *ARGFX *transcription in humans [6]. Here we undertake a comparative study of *ARGFX *sequences in vertebrate genomes to investigate the origin, the patterns of mutation and gene loss, and the extent of evolutionary conservation of this locus.

## Results and Discussion

### Human *ARGFX *gene and transcript

**Figure 1:**
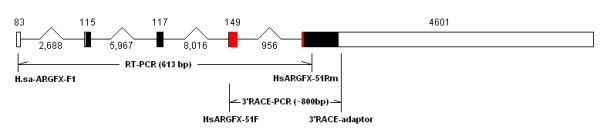
**Gene structure of human *ARGFX***. PCR primer positions and amplicons are shown relative to predicted human *ARGFX *gene structure [6]. Boxes indicate exons, drawn to scale; lines indicate introns, not drawn to scale. Numbers above boxes and beneath lines indicate the lengths of each exon and intron. The 5' and 3' untranslated regions are shown in white and the protein coding regions are shown in black, except for the homeodomain which is red.

Although human *AGRFX *has been shown to be weakly expressed in human testis and ES cells [6], the full transcript has not been previously cloned or experimentally verified. Indeed, only one of the four predicted intron positions in the human *ARGFX *was originally verified from cDNA (intron 4); the other three intron positions were predicted by sequence comparison to two retroposed pseudogenes deduced to be derived from *ARGFX *[6]. We therefore designed a range of *ARGFX*-specific forward and reverse primers and conducted RT-PCR and RACE PCR on human testis RNA. 5' RACE PCR was unsuccessful, but 3' RACE generated a product that was polyadenylated approximately 30 nucleotides downstream from the predicted stop codon. This termination may be artificial or an alternative polyadenylation site; we note that it is preceded by a canonical AATAAA polyadenylational signal. RT-PCR using forward and reverse gene-specific primers successfully amplified from exon 1 to exon 5, verifying that all four predicted introns are faithfully spliced out in a processed human RNA product (Figure 1). The assembled sequence has the potential to code for a protein of 315 amino acids including a 60 amino acid homeodomain. We conclude that the original annotation was correct, at least across the putative coding region of the *ARGFX *locus. This annotation includes the unusual exon 3 noted by Booth and Holland [6] which is comprised from the 'right arm' of an *Alu *element. Interestingly, the transcriptional direction of the *ARGFX *locus is opposite to that expected for parental *Alu *elements; this is consistent with the finding of Gal-Mark et al. [9] that most exonizations of *Alu *elements occur in the right arms of antisense *Alu *elements.

Since the *ARGFX *mRNA is present at very low levels, we suspected that detection of a protein product would be difficult. We carried out western blots on human testis total protein using a commercial polyclonal antibody and detected a single band of 50 kDa (data not show); this is much larger than the expected 36 kDa. The size discrepancy suggests the band is artefactual and may reflect the fact that the antibody was raised to the whole protein including the Alu region. We note that exonized coding *Alu *elements are also found in several proteins; examples include DSERG1 (GeneID 751816), ZMAT1 (GeneID 84460) and POLR3B (GeneID 55703). In addition, we searched the PeptideAtlas http://www.peptideatlas.org/ and PRIDE database http://www.ebi.ac.uk/pride/ and found four matches in the latter. However, these spectra did not pass the filtering criteria used, and thus do not provide conclusive evidence for translation of human *ARGFX *gene.

### The evolutionary origin of *ARGFX *sequences

**Figure 2:**
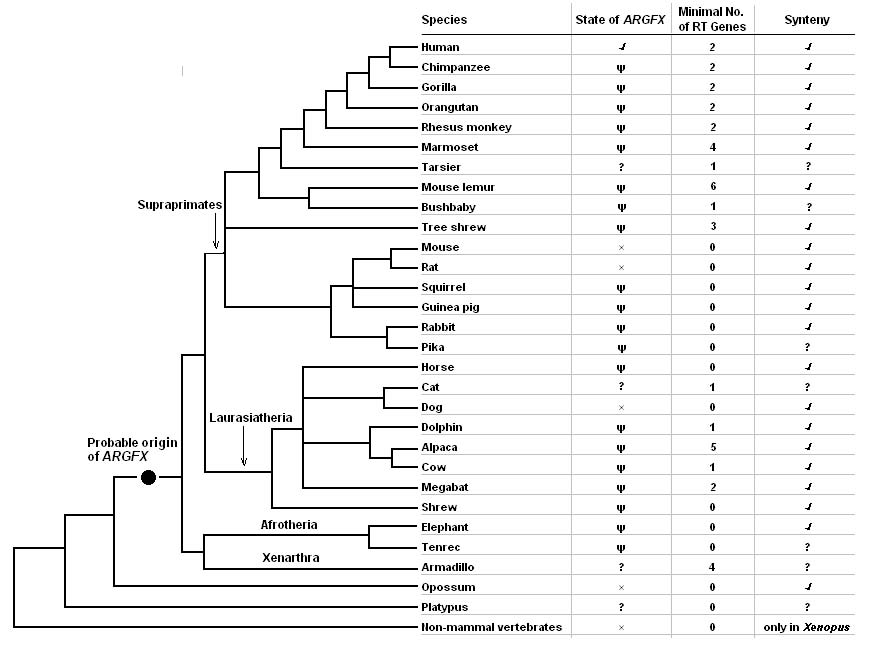
***ARGFX *gene in vertebrates**. The phylogenetic tree of mammals is based on [29-32]. Column 1: *ARGFX *is inferred to be a probable gene (√), possible pseudogene with disrupted coding region (ψ), secondarily completely lost (×) or not included in current genome data (?). Column 2: Minimal number of retrotransposed *ARGFX *pseudogenes based on current genome data. Column 3: Synteny with the human *ARGFX *genomic region is conserved (√) or not (×), or not sequenced (?).

The *ARGFX *locus was first identified in the human genome, but no homologous sequence was found in mouse, even though the syntenic region is readily identified [6]. To trace the origin of *ARGFX*, we searched a wide phylogenetic range of vertebrate genome sequences using tblastn. We detected *ARGFX*-related sequences in many, but not all, placental mammals (Figure 2; Additional file 1). *ARGFX*-related sequences with or without predicted introns were identified in chimpanzee, gorilla, orangutan, rhesus macaque, marmoset, tarsier, mouse lemur, bushbaby, tree shrew, squirrel, guinea pig, rabbit, pika, horse, cat, dolphin, alpaca, cow, megabat, shrew, elephant, tenrec and armadillo. No closely similar matches were detected in mouse, rat, dog, non-placental mammals (opossum and platypus) or non-mammalian vertebrates (or indeed invertebrates),

To further refine these results, we used a more sensitive method that exploited the fact that *ARGFX *has readily recognized flanking genes within an easily identified syntenic region. Starting with the human *ARGFX *genomic region, we were able to identify the corresponding genomic region in virtually all placental mammals, plus opossum (a non-placental mammal) and a frog (*Xenopus tropicalis*). We then used mVISTA to search these regions at low stringency for any sequences related to human *ARGFX*. In neither opossum nor frog was there detectible *ARGFX*-related sequence in the corresponding region. The locus was also missing from the syntenic region of mouse, rat and dog.

By combining the sequence similarity searching and the synteny analyses with a probable phylogenetic tree of the vertebrates, we can deduce the likely date of evolutionary origin of *ARGFX *homeobox loci (Figure 2). The absence of the locus in mouse, rat and dog clearly reflects secondary loss, since these species are nested within the tree of placental mammals. The minimal inclusive clade containing taxa possessing *ARGFX *sequences encompasses Xenarthra, Afrotheria, Supraprimates and Laurasiatheria. In contrast, there is no evidence that *ARGFX *sequences existed before the origin of placental mammals. We propose, therefore, that *ARGFX *originated after the divergence of Eutheria from Metatheria.

**Figure 3:**
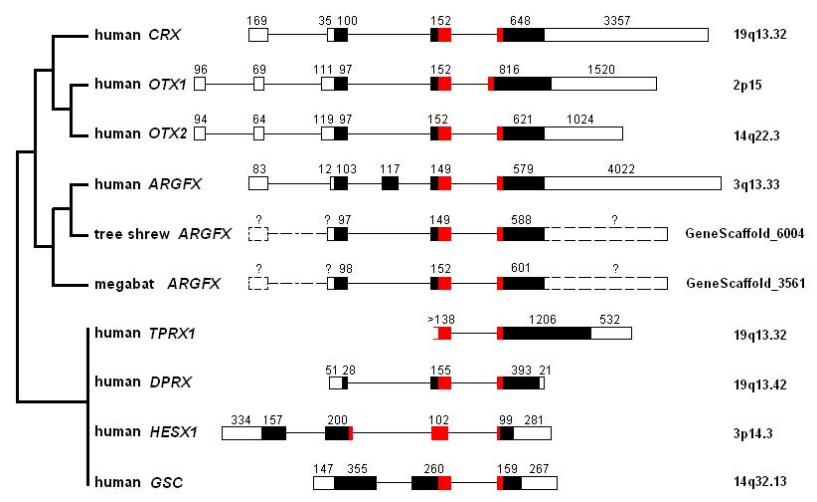
**Comparison of *ARGFX*, *OTX1*, *OTX2 *and *CRX *gene structures**. Human *TPRX1, DPRX*, *HESX1 *and *GSC *gene structures were used as references. Exons are represented by boxes and introns by lines, with the length in nucleotides written above. The 5' and 3' untranslated regions are shown in white and the protein coding regions in black except for homeodomains which are shown in red. Human gene structures follow the NCBI gene annotation; tree shrew and megabat *ARGFX *intron positions were deduced by reference to retroposed pseudogenes.

**Figure 4:**
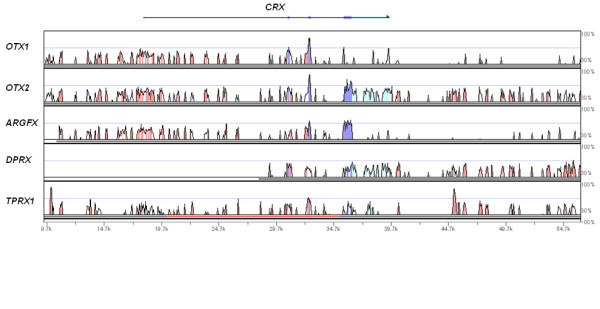
**Greater conservation of DNA sequences between human *ARGFX*, *OTX1 *and *OTX2 *genomic regions than with *DPRX *and *TPRX1***. Human *GSC *and *HESX1 *were also used as references, but no similarity was found. Genomic sequences from the last base pair of upstream gene to the first base pair of downstream gene for each locus (based on UCSC at http://genome.ucsc.edu/ were used, and compared using Shuffle-LAGAN [28] in mVISTA, which can detect sequence rearrangements. Coloured peaks (purple, coding; pink, intergenic; blue, transcribed non-coding) indicate regions of at least 30 bp and 30% similarity.

**Figure 5:**
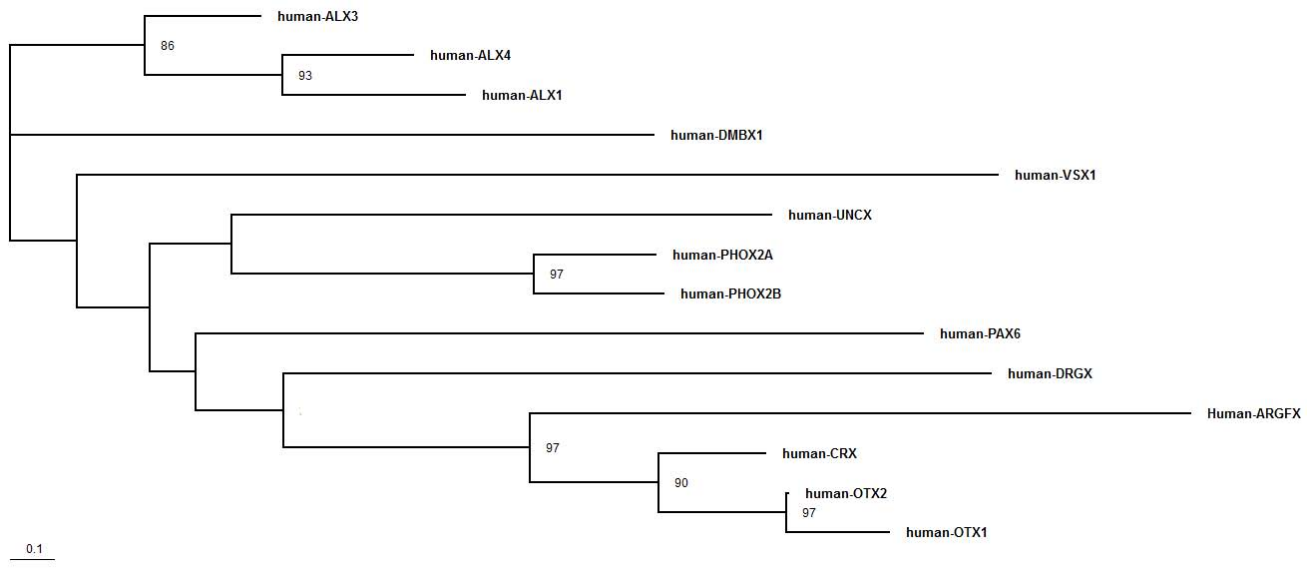
**Phylogenetic relationship between *ARGFX *and other PRD class homeobox genes**. Maximum likelihood phylogenetic tree constructed using complete deduced human ARGFX protein sequence and the most similar human homeodomain proteins. Bootstrap support values over 50% are shown. Essentially the same topology was recovered by Bayesian analysis except at weakly supported nodes, notably the position of VSX1.

The human *ARGFX *gene has similar exon-intron organisation to the human *OTX1, OTX2 *and *CRX *genes as depicted in Figure 3. The latter three genes are part of a gene family, *Otx*, which is well conserved across the Metazoa. Although human *ARGFX *gene has an additional coding exon compared to the three *Otx *genes, this derives from an Alu element (see above) and we find this is not present in the predicted tree shrew and megabat *ARGFX *genes (Figure 3). This is consistent with the observation that *Alu *elements are primate-specific repetitive sequences [10]. In addition to similar gene organisation, we also detected patches of shared sequence similarity in exons and surrounding genomic regions between *ARGFX *and the three canonical *Otx *genes (Figure 4). A phylogenetic analysis using complete predicted protein sequences reflects this, as it groups *ARGFX *with *OTX1*, *OTX2 *and *CRX *(ML 97% bootstrap; Bayesian 1.0 support value; Figure 5). One possibility is that *ARGFX *originated by gene duplication from one of the three canonical vertebrate *Otx *genes, on the stem lineage of the eutherian mammals. If this occurred, it must have been followed by extensive sequence divergence because the human ARGFX homeodomain shares only 55% to 56.7% amino acid sequence identity with human *Otx *family genes over 60 amino acids (genes within a homeobox gene family generally share 70 to 100% homeodomain amino acid identity [7,11]).

If *ARGFX *did originate as proposed, it is not clear which *Otx *gene was the progenitor, since *ARGFX *is on a different chromosome to all three candidates. Interestingly, the canonical *Otx *genes share more sequence similarity with *ARGFX *than they do with *DPRX *and *TPRX1 *which are located close to *CRX *chromosomally (Figure 4).

**Figure 6:**
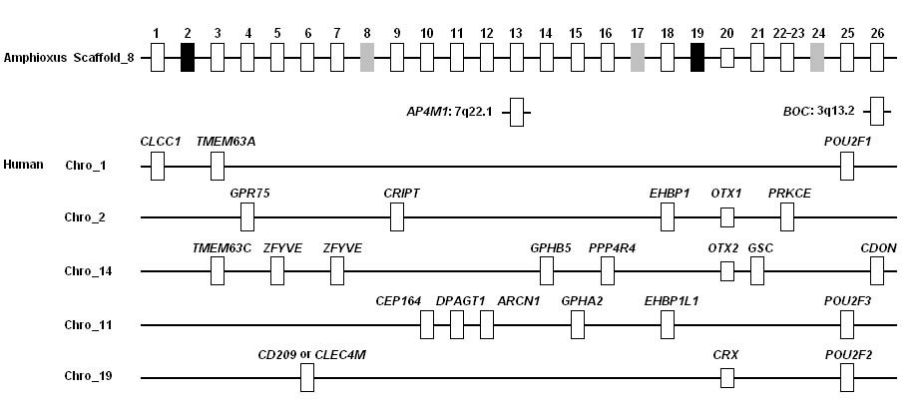
**Synteny and paralogy around the *Otx *gene family**. Map positions of amphioxus *Otx *and its neighbouring genes are compared to their human orthologues, which map primarily to chromosomes 1, 2, 14, 11 and 19, not chromosome 3. Amphioxus genes are shown in their physical order, and are numbered as in amphioxus (*B. floridae*) genome assembly v. 1.0. GeneID 20 is amphioxus Otx. GeneID 22 and 23 are most likely two parts of a gene and are treated as one locus. Human orthologues are not necessarily in order. Amphioxus genes 2 and 19 (black boxes) do not have clear human homologues; phylogenetic relationships are not well resolved for amphioxus genes 8, 17 and 24 (grey boxes). Human orthologues of amphioxus gene 13 do not map to on the five main chromosomal regions.

If we accept the proposal that *ARGFX *is a duplicate either of *CRX, OTX1 *or *OTX2*, then the vertebrate *Otx *gene family comprises four genes (or indeed more, if *TPRX1 *and *DPRX *originated by a similar mode). The number four is interesting because it is now clear that two rounds of whole genome duplication (2R) occurred in early vertebrate evolution [12,13]. These events expanded each ancestral chordate gene to a complement of four, before subsequent gene losses. Although we hypothesize that *ARGFX *originated on the stem lineage of the eutherian mammals, it is also worth testing an alternative hypothesis that *ARGFX *is actually the 'cryptic' fourth *Otx *paralogue dating to the much more ancient 2R genome duplications. Such a hypothesis would predict that the three canonical *Otx *genes, plus *ARGFX*, map to a fourfold paralogy region in the human genome, homologous to a single chromosomal region in amphioxus. To test this we identified 24 genes neighbouring the amphioxus *Otx *gene (scaffold 8; *B. floridae *genome assembly v1.0, http://genome.jgi-psf.org/Brafl1/Brafl1.home.html) and found their human homologues, verifying each by constructing neighbor-joining phylogenetic trees. Examining their chromosomal locations revealed that most human orthologues are distributed on four human chromosomes (2, 10, 11, 14), three of which accommodate *OTX1*, *OTX2 *and *CRX*, plus a smaller number on chromosome 1 (possibly reflecting breakage of a fourfold paralogy region; Figure 6). In contrast, only one human orthologue (*BOC*) was found on chromosome 3, the location of *ARGFX*. This result implies that while *OTX1*, *OTX2 *and *CRX *were generated by two rounds of genome duplication in early vertebrate evolution, *ARGFX *was not.

In summary, we propose that *ARGFX *originated after the divergence of Eutheria from Metatheria. This view is strongly supported by the phylogenetic distribution of *ARGFX *sequences in mammals and by analysis of the *ARGFX *syntenic region in placental and marsupial mammals and an amphibian. An alternative hypothesis, that the origin of *ARGFX *dates to genome duplication events in early vertebrate evolution, gains no support from paralogy analysis. It is likely, therefore, that the eutherian mammal *ARGFX *locus originated by gene duplication from *CRX*, *OTX1 *or *OTX2*, followed by extensive sequence divergence from these conserved *Otx *genes.

### Mutation in placental mammals

Although *ARGFX*-related sequences were identified in most placental mammals examined, we were surprised to find that each one carried critical sequence mutations when compared to the human *ARGFX *open reading frame. In no case did a non-human *ARGFX *locus contain an intact open reading frame of equivalent length to the human sequence (Figure 1), and hence we conclude that none can code for a functional protein. Furthermore, each lineage of mammals has different disabling mutations (Additional file 2). For example, within the primates, chimpanzee and gorilla have the start codon ATG mutated to ACG, in orangutan the stop codon TGA is mutated to TCA causing termination four codons further downstream, macaque has a four base-pair insertion causing a frameshift, and marmoset and mouse lemur each have different stop codon mutations resulting in a shorter protein. In other mammals, tree shrew has two frameshift mutations and one premature stop codon, guinea pig has four separate frameshift deletions in the C-terminus, cow has a ten base pair deletion at the N-terminus causing a frameshift and a pair of two-nucleotide deletions at the C-terminus, horse has a one nucleotide insertion, two premature stop codons and deletions within the homeobox, while megabat has three frameshift mutations and a premature stop codon. As already mentioned, mouse, rat and dog have lost the locus secondarily.

These results are particularly surprising when considered in relation to the phylogenetic tree of mammals (Figure 2), because the range of species in which mutation of the *ARGFX *locus is found do not form a monophyletic group to the exclusion of humans. There are several possible explanations for this unusual pattern. First, *ARGFX *may be a non-functional pseudogene in all mammals, and the intact open reading frame in humans is simply a stochastic variant of a sequence diverging in a neutral manner. This would imply a single loss of function event soon after the origin of *ARGFX *in the eutherians. Second, the *ARGFX *locus may have been functional for the entire evolutionary history of humans, and is still functional, but has become a pseudogene (or has been lost) in many independent mammalian lineages. This would imply at least 10 independent loss of function events in mammalian evolution, if the phylogeny used in Figure 2 is correct. Third, the gene may have 'flickered' between functional and non-functional states in the evolutionary history of mammals. This might imply a smaller number of independent loss of function events, yet selective maintenance of function for at least part of human evolutionary history. Fourth, the locus may be functional as an RNA but not as a protein in most mammals.

### Signatures of selection in *ARGFX *evolution

The unusual evolutionary pattern prompted us to reconsider whether human *ARGFX *locus encodes a functional gene, or indeed whether *ARGFX *has ever been functional. Detection of a transcription product from the locus, or even a putative translation product, is not sufficient evidence; many pseudogenes are transcribed [14,15]. Finding a human phenotype associated with mutation of *ARGFX *would be informative, but so far no such human condition is known. We therefore addressed this question from an evolutionary perspective. Two approaches were employed.

First, we tested whether there were signatures of positive or negative selection pressure in the coding region by calculating the ratio of numbers of nonsynonymous to numbers of synonymous substitutions per site, *dN*/*dS *(ω). We used human, chimpanzee and orangutan *ARGFX *sequences to facilitate accurate alignment. Among the three pairwise ω ratios, one is greater than 1.0 and the other two close to 1.0 (table 1). We then divided the coding region into three parts, N-terminal, homeodomain and C-terminal, and calculated ω values separately. All three pairwise ω values for the N-terminal part were more than 1.0, but ω values for the other two parts are far below 1.0, suggesting that the N-terminal part has encountered positive selection while the homeodomain and C-terminus sequences have been strongly constrained by negative selection. Plotting potential positively selected sites onto the open reading frame revealed nearly half of them (6/14, posterior probability > 80%) are in the region coded by the *Alu *element (data not shown). To control for the possibility that the *Alu *element is affected by evolutionary processes beyond *ARGFX *functional constraints, we removed the *Alu *element and re-calculated the nonsynonymous to synonymous ratio. As expected, the ω values for the full sequence dropped significantly and were now below 1.0 (p < 0.05, Z-test). Taken together, these results argue that human *ARGFX *is a functional gene with some sites under positive selection pressure, but the majority of the sequence is under negative or purifying selection.

**Table 1:** *dN *and *dS *values in different analyses

	**whole coding region with Alu**			**N-terminal (231 bp)**			**Homeodomain (183 bp)**			**C-terminal (495 bp)**			**whole coding region without Alu**		
	
	**dS**	**dN**	**dN/dS**	**dS**	**dN**	**dN/dS**	**dS**	**dN**	**dN/dS**	**dS**	**dN**	**dN/dS**	**dS**	**dN**	**dN/dS**
	
human-chimpanzee	0.008	0.012	1.500	0.000	0.044	NA	0.022	0.000	0.000	0.006	0.003	0.500	0.009	0.007	0.778
human-orangutan	0.032	0.024	0.750	0.017	0.056	3.294	0.044	0.015	0.341	0.033	0.014	0.424	0.032	0.015	0.469
chimpanzee-orangutan	0.032	0.028	0.875	0.017	0.076	4.471	0.022	0.015	0.682	0.040	0.014	0.350	0.031	0.020	0.645

**Figure 7:**
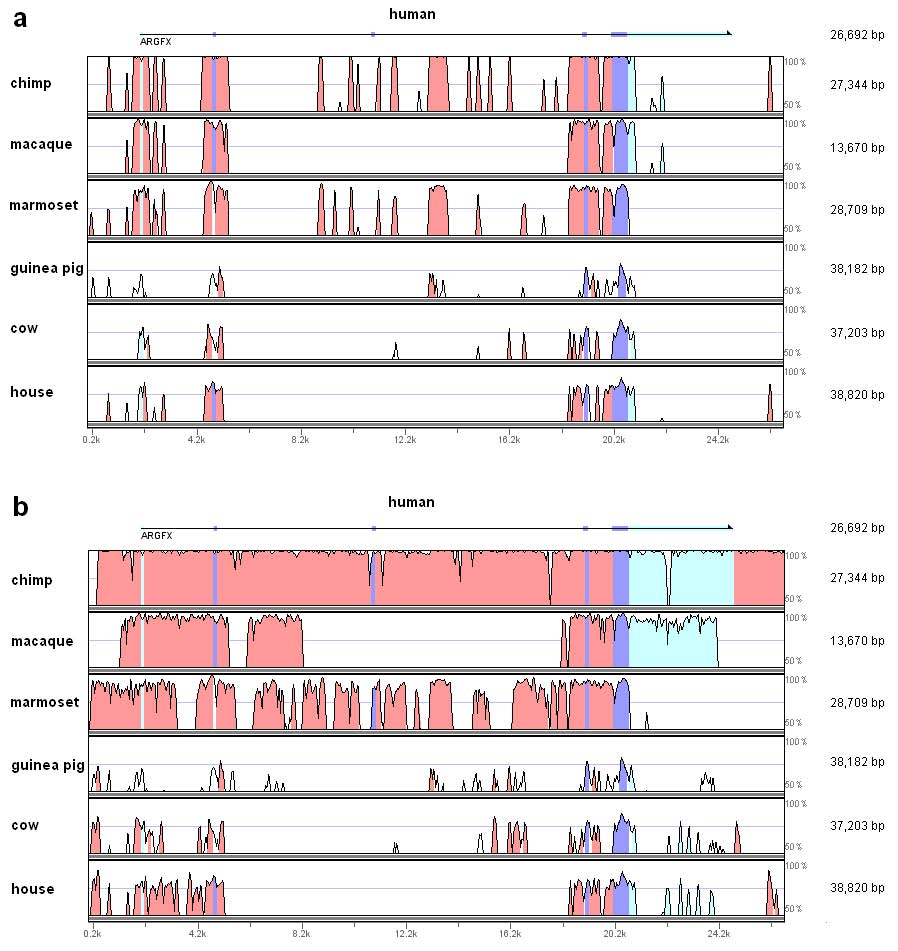
**Conservation of DNA sequence conservation between mammalian *ARGFX *genomic sequences**. Only species with a high genome assembly in this region were used. Sequences were aligned by the LAGAN program [17] in mVISTA. Length of the genomic region for each species is on the right. Macaque is missing a region between exon 2 and exon 4 accounting for higher similarity between human and marmoset than human and macaque in this region. The higher sequence similarity in and around exons is clearly visible, indicative of selective constraints since divergence of the species shown. Coloured peaks (purple, coding; pink, intergenic; light blue, UTR) indicate regions of at least 50 bp and 50% similarity. a. mVISTA plot using repeat-masked genomic sequences; b. mVISTA plot using sequence with no masking of repeats.

The second approach taken was to compare more widely divergent mammals and examine the extent of conservation in exonic and intronic sequence. If the gene has been functional during much of mammalian evolution, we predict that exonic sequence would be more highly conserved than intronic sequence. Multiple alignments revealed that *ARGFX *exons (and sequences immediately flanking exons) show much higher conservation than introns between divergent lineages (Figure 7). Furthermore, the level of conservation is not uniform between regions of the deduced protein, but is much higher within the homeodomain than the N-terminal and C-terminal regions (mean Poisson corrected distance: 0.280 vs 0.851 and 0.461). Among 45 possible pairwise comparisons between 10 species, all distances for homeodomains are much less than corresponding values for the N-terminal region and only 8 of them are slightly greater than corresponding values for the C-terminal region (Additional file 3). These results indicate that the coding sequence of the *ARGFX *homeobox locus, and in particular the homeobox, has been under purifying selection for much of mammalian evolution.

## Conclusion

In this study, we have performed a deep comparative phylogenetic and phylogenomic analysis on the recently described homeobox locus *ARGFX*. We present evidence that this gene originated by gene duplication from *Otx1, Otx2 *or *Crx *during early mammalian evolution. The most likely origin was on the stem lineage of the eutherian mammals, after their divergence from marsupials and monotremes, and before the radiation of living placental mammals. The gene diverged extensively in sequence from its progenitor homeobox gene and was then subject to purifying selection. This purifying selection was maintained through much of placental mammal radiation, even though the coding sequence of the locus is disrupted in most mammalian genomes analysed. Strangely, human is the only mammal studied to date in which the full open reading frame is retained, and we suggest that the *ARGFX *locus is still functional in human. Indeed, we detected a transcript in human testis that has the potential to encode the full deduced protein. The unusual pattern of conservation suggests either that there have been very many independent losses of function for this gene in mammalian radiation, or, that the gene encodes a functional RNA molecule. An alternative hypothesis is that the gene has 'flickered' between functional and non-functional states in the evolutionary history of mammals, particularly during the late Cretaceous period when many mammalian lineages diverged in a relatively short timespan.

## Methods

### cDNA cloning of human *ARGFX*

Human testis total RNA was obtained commercially from AMS Biotechnology Ltd. (Cat. No. R1234260-50), and used to synthesize cDNA using a SMART RACE kit (Clontech Laboratories Inc.). Based on open reading frame predictions made from genomic DNA, gene-specific primers were used to amplify and clone *ARGFX *cDNA covering the complete predicted open reading. Forward primers used were: H.sa-ARGFX-F1 (CACGTAGGACTGAAAATGGTTACTC in exon 1) and HsARGFX-51F (CGGAGAAGGCATAAAGAACG in exon 4). Reverse primer was HsARGFX-51Rm (AGGGTCTAAGGGCTGAGATGG in exon 5). Primer positions are shown in Figure 1. To examine whether the transcribed mRNA is translated, western blots were performed using human ARGFX polyclonal antibody (Abcam Inc., Cat. no. ab67562) and human testis total protein (AMS Biotechnology, Cat. no. P1234260).

### Identification of *ARGFX *sequences in other vertebrates

Initially, we focused attention on nineteen vertebrate genome sequences of high quality in Ensembl (release 56) accessible at http://www.ensembl.org/index.html, comprising ten mammals (chimpanzee, orangutan, rhesus macaque, mouse, rat, dog, cow, horse, opossum, platypus), two birds (chicken, zebra finch), one reptile (anole lizard), one amphibian (*Xenopus tropicalis*) and five teleost fish (*Tetraodon*, *Takifugu*, medaka, stickleback, zebrafish). Genomes were searched using tblastn [16] with human *ARGFX *deduced protein sequence as the query and an E-value cutoff of 1e^-5^. Many partial genome sequences were also searched. In cases where no match was detected, this can be due either to incomplete sequencing, complete absence or to partial degeneration. To distinguish between these possibilities, we identified the region of each genome assembly syntenic to the human *ARGFX *location and searched this for short degenerated sequences related to *ARGFX *using the LAGAN program [17] implemented in mVISTA http://genome.lbl.gov/vista/mvista/submit.shtml with a match criterion of 50% identity over 50bp. All sequences used in analysis are included in Additional file 1.

### Phylogenetic analysis

Nucleotide sequences and deduced protein sequences were aligned using CLUSTAL_X [18]. NJ (Neighbor-Joining) trees [19] were calculated using MEGA4 [20] using the Kimura 2-parameter distance estimation for nucleotide sequences and Poisson distance estimation for protein sequences [21]. ML (maximum likelihood) trees were constructed using PHYML [22] using the JTT model, estimated to be the most appropriate model by ProtTest [23]. The reliability of interior nodes in ML and NJ tree was assessed by bootstrapping with 100 replications [24]. Bayesian trees were constructed using MrBayes 3.1 with 1.2 million cycles and sampling 1/10 of trees after a 30,000 cycle burn-in [25]. Deduced protein sequences used for ARGFX trees were human ARGFX (without the region encoded by the *Alu *element), NP_001012677, and the thirteen most similar human proteins according to blastp: human CRX, NP_000545; human OTX2, NP_068374; human OTX1, NP_055377; human UNCX, NP_001073930; human ALX1, NP_008913; human PHOX2B, NP_003915; human PAX6, NP_001595; human DRGX, NP_001073989; human DMBX1, NP_671725; human ALX4, NP_068745; human PHOX2A, NP_005160; human VSX1, NP_055403; human ALX3, NP_006483. Numbers of synonymous (*dS*) and nonsynonymous (*dN*) per site were calculated using the modified Nei-Gojobori Jukes-Cantor method [26] implemented in MEGA4. Potential positive selected sites were determined using codeml program in PAML 4 package [27]. Alignments of genome sequences were undertaken using LAGAN [17] or Shuffle-LAGAN programs [28] implemented through mVISTA http://genome.lbl.gov/vista/mvista/submit.shtml.

## Abbreviations

*ARGFX*: (*Argnine-fifty homeobox*); *dS*: synonymous; *dN*: nonsynonymous; RACE: Rapid Amplification of cDNA Ends.

## Authors' contributions

PWHH designed the study. GL carried out database searching and performed experiments and data analyses. PWHH and GL drafted the manuscript. Both authors edited and approved the final manuscript.

## Supplementary Material

Additional file 1 ***ARGFX sequences used in this study *(MS Word format)**. Click here for file

Additional file 2 **Alignment of eleven ARGFX coding sequences showing mutations leading to frameshifts (in grey shade) or stop codons (in red shade)**. The homeobox is underlined. Click here for file

Additional file 3 **Pairwise poisson-distances between ten deduced ARGFX protein sequences**. Click here for file
