# Supplementary material for: The origin and evolution of ARGFX homeobox loci in mammalian radiation
Source: BMC Evol Biol. 2010 Jun 17;10:182. doi: 10.1186/1471-2148-10-182 (PMC2894831; doi:10.1186/1471-2148-10-182)
Supplement: Additional file 1 — ARGFX sequences used in this study (MS Word format). [file 1471-2148-10-182-S1.DOC]

**Additional file 1**

*ARGFX sequences used in this study*.

>Human-ARGFX_NM_001012659

ATGAGGAACAGAATGGCCCCAGAGAATCCCCAGCCAGACCCTTTCATCAATAGGAATTAT

TCCAACATGAAGGTGATACCACCACAGGATCCAGCTAGTCCCAGTTTCACTCTGTTATCC

AAGCTGGAGTGCAGTGGCACGGTCTCGGCTTACTGCAGCCTCAACCTCCCAGGTTCAACT

GATCCTCCCACCTCAGCCTCCCGAGTAGCTGCGACTACAGCAATACGGAGAAGGCATAAA

GAACGTACTTCTTTCACCCACCAACAGTATGAGGAGCTAGAAGCTCTGTTTAGCCAGACC

ATGTTCCCAGATAGAAATCTTCAGGAGAAACTAGCTTTGAGACTCGACCTACCGGAGTCA

ACAGTAAAGGTTTGGTTCAGGAACCGGCGATTCAAATTGAAGAAGCAGCAGCAGCAGCAA

TCAGCAAAGCAACGAAACCAGATCCTTCCATCCAAGAAGAATGTGCCCACCTCCCCCAGA

ACATCCCCCAGTCCTTATGCTTTTTCTCCTGTGATTTCAGATTTCTACAGCTCCCTTCCA

TCTCAGCCCTTAGACCCTTCCAATTGGGCATGGAACTCTACCTTCACTGAGAGTTCTACC

AGTGACTTCCAAATGCAAGATACTCAGTGGGAGAGGCTGGTGGCCTCGGTTCCTGCTTTG

TACTCTGATGCCTATGACATATTCCAAATCATAGAACTGTACAATCTTCCTGATGAGAAT

GAGATATCCAGCTCTTCTTTCCACTGTCTGTATCAGTATCTCTCACCCACAAAGTACCAG

GTAGGAGGACAGGGTTCCTCTCTCAGCATCTTTGCTGGTCCAGCTGTAGGCCTATCTCCT

GCACAAACCTGGCCCAATATGACAAGCCAAGCCTTTGAAGCCTACAGTCTAACAGATAGC

CTGGAATTCCAGAAAACCTCCAATATGGTAGACTTGGGATTTCTCTGA

>Human-ARGFXP1-NT_034772

TTCTTAGAAGAAATGTCTATCCACACATTTGCCCATTGTTTAATTGGGTTATTTGTTCTT

TTATTGTTGAGTTGGATACGGATGTTATTTAAAAATTTTTGGACACACTAAGGAAACTGC

ATTTCCAGAGAGAGACACACCATGCAGGACTGAAAATGGTTAGTCTAAGGGGATTCATGA

CAGATTTCAAAAACCATGATGAACAGAATGGCCCCAGAGAATTTCCAGCCAGACCCTTTC

ATCAACAGGAATGATTCCAACATGAAGGTGATACCACCACAGGATTCAGCTAGTCCCAGT

TTCAGTCTGTCATCCAAGCTGGAGTGCAGTGGCATGGTCTCCACTCACTGCAGCCTCAAC

CTCCCAGGTTCAAGTGATCTTCCCACCTCAGCCTCCCGAGTAGCTGGGACTACAGCAATA

TGGAGAAGGCATCAAGAACGTACTTCATTCACCCACCAACAGTATGAAGAGCTAGAAGCT

CTGTTTAGCCAGACTATGTTCCCAGATAGAAATCTTCAGGAGAAACTAGCTTTGAAACGC

AACCTACTGGAGTCAACAGGTAAAGGCTTGGTTCAGGAACTGGCAATTCAGATTGAAGCA

GCAGCAGCAGCAGCAGCAGCAGCAGCAATCAGCAAAGCAAGCAAACCAGATCCTTTCATC

CAAGAAGAATGTGCCCACCTCCCCTAGAACATCCCCAAGTCCTTATGCTTTTTCTCCTGT

GGTTTCAGATTTCTACAGCTCCCTTTCTCCTCAGCCCTTAGACCCTTCCAATTGGGCCTG

GAACTCCACCTTCACTGAGAGTCCCACAGGTGACTTCCAAATGCAAGATACTCAATGGGA

GAGGCTGGTGGCCTCAGTTCCTGCTTTGTACTCTGATGCCTGTGACATATCCCAAATCAT

AGAACTGTACAATCTTCCTGATGAGAATGAGATATCCAGCTCTTCTTTCCACTGTCTGTA

TCAGTATCTCTCACCCACAAGGTACCAGGTAGGAGGACAGGGTTCCTCTCTGAGCACCTT

TGCTGGTCCAGCTGTAGGCCTATCTCCCACACAAACCTGACCCAGTATGACAAGCCAAAG

CTTTGAAGCCTACAGTCTAACAGACAGCCTGGAATTCCAGAAGACCTCCAATATGGTAGA

CTTTGGATTTCTCTGACCAGAGTACTAATAAATATGGACCATTTAGAAAAGAGGTCTTCT

TGCCTCTTGTACATGACTTTTTTCTCCTTTGTCTCATTTTAACGCAAACATCTGGGGCTG

TGTCTCTGATTTCCATGGAAATGTTGCAAAAAGATTTTTCCAAGTAGAGCTGGGCACTAT

GCAATCAGCCCCACAATTCTTCCTGAGAAGCCTTCCTAGTCCCTTACATGGCCAATGAGA

CTCCCAAATTCCCTTCCCAAAATTATCCTGATTTTCTAAAAGTAGAACAGTGGTTTTGGG

AGTTATCCACTTTATTTTTAAAAAACTAGGTCTCTCTCTGTCACCCAGGCTGGAATGCAG

TGGAACAATCATGGCTCACCAGAGCCTTGACCTCCTGGGCTCAGGTGATTCTCCCACCTC

AGCCTCCTGAGTAGCTGGGACTACAGGTGCCCGCCACCACACTCGACTAATTTTTTGTTT

TTTTGTTTGTTTGTTTTTTGAGACAGAGTTTCACACTGTCACCCAGGCTGGAGTGCAGTG

GCGGGATCTCGGCTCACTGCAACCTCCACCTCCCAAGGTTCAGGCGATTCTCCTGCCTCA

GCCCCCTGAGCGGCTGGGATTACAGGTGCATGACACCATGCCCGGCTAATTTTTGTGTTT

TTGGTGGAGACAGGGTTTCACCGTGTTGCCCAGGCTGGTCTCGAACTCCTGACCTCCAGT

GATCCACCTGCCTTGACCTCCCAGGGTGTGGGATTGCAGGCATGAGCCACCCAGCCCGGC

CAATTTTTGTATTTTTTGTAAAGGCAAAATTTTGCCATGTTGCCCTGGCTGGTCTCGAAC

TCCTGGACTTAACATCATCCACCTGCCTCAGCCTCCCAAAGTGCTAGGAATACAGGAGTG

AACCATCGCACCCAGCTGGGTTATCCAGTTTAAAAGAATTTTTTTTTCAAGGAAACCAGA

GAAGCCATCATCCACTTTTCTGTCTGTATCATTCCGATTTTAGTATATGTGTTGCCGAAG

TGAGTATACACTATCATCCACTTTTAATGAGAGAAAGTTATAAACTTACATTTTGTTTCC

AATTTTTAGGGTTTTTTTTTTTTTTTTTTTTTTTTTTTTTTGAGACAGCACTCTGCCTCC

AGGCTGGAGTGTGCAGTGGCACCATCTTGGCTCACTGCAGCCTCTGCCTCCTGGTCTCTA

ACAATCCTCCCACCTCAGCCTTCTAAGTGGCTGGGACTACAGATGAGCGCCACCATGCCT

GGCTAATTTTTGTATTTTTAGTGGAGAAGGGGTTTCGCCATGTTGCCCCAGCTGAATTTG

GGATTTTTTTAAAGGGGACTTTCAGTCCAAATTTGAGGGGAAATTACTATCACCACTAGT

TTGAATATAGTACAATGAAAAAAATATATATATAGTACAATGGTCCAGACATAATAAGAG

GTGTAAAAACTAGCACAGGAAGAAATAAAAACAGATTTTTGAAGGTGAGATGAGAAACTC

ACAAAATTAACAAAATAAATAGCCAAACTTTTAGAATGACATTTTAGCACATTTTCCAGC

CACATGATCAATCTACAAAATTAATATTATTTCTGAACACCAGTAATAAACATAAGGTAA

GTACCTCTCAATAAAAAAATAGAAAAGTAGCTAGGAATTCACAAAGAAAATTAATACCTC

TGTGGAGAAAACTTCAAAACTTAAAAAAAGACATAGAAAATGTTTTGAATATGCTGGGTG

CAGTGGCTCACACCTGTGGTCCTGGCACTTTGGGAGTCCAAGGCAGGTGGATCATGGGGT

CAGGAGACCAAGACCATCCTGGCCAGAATGGTGAAACCCCGTCTCTACTAAAAATACCAA

ATAAATTAGCTGGGAGTGGTGGCACATGCCTGTGGTCCCAGCTACTCAGGAGGCTGAGGC

AGGAGAATCACAAGGATTGAAATTATGCAGTGTGTTCTCCAAACACGATGGAATGAAATT

AGAAATCAATAGTGGAAGAAAATTTGGGAAATTCACACATATGTGGATATTAAACAACAC

ATTCCTAAATAACCAATGGTTAAAGGGGAAATCACAGTCTGAGCAACATGGCAAAACCCT

GACTCTGCAGAAAATGGAAAATTAGCTGGGCATGGCAGCCAGAGCCCACGCTCCCAGCTA

CTCAAGAAGCTGAGGTGAGAGAATCTATCCCCTGAGCCACAGAGGTCGAGGCTGCAGTGA

GCAGTAACTGTGCCACTGCACTCCAGTTTAAGCAACACAGTGATACCATCTCAATAAATA

AATAAATAAATAAATAAATAAATAAATAAATAGAAGAAATCATAAGTAAAATTGGAAAAC

ACTTCTAACTGAAAAAAAATAAAAACAGAAGCACAACAAACCAAAACATGGGATGAAGCT

AAATCAATGCTTAGAAGGAAATTTGTAGCTTCAAATACTTATATATGTTTTTTAAAAAGG

TCTCAGATCAATAAGTTTTATACAGGAAGGAAGCTGGCATAGTTTTATTAATATTTGTCA

AATTAAACTTTAAGGCAAAACTTTTTTATTGAGATAGAGCCTTGCTCTGTCGCCCAGGCT

GGAGTGCAGTAGTGCAATCTTGGCTCACTGCAACCTTCGCCTCCAGGGTTCAAACAATTC

TCCTGCCTTAGCCTCCCAGGTGGCTGGGACTACAGGTGTGAGCCACCATGCCCGGCTAGT

TTTTGTATTTTTAGTAGAGACGGGGTTTTGCCATGTTGCTCAGGCTGGCCTCAAACTCCT

GAGCTCAAGTGATCCTCCCACCTCAGCCTCCCAAAGGGCTGAGATTGCAGGTGTGAGCCA

CCTTGCCTGGCCAAAACAGTTTTTTAGAAATAAAGTTTATTAGGCAACAAGAAAAGGAAC

AATTAACCTGAAATATATGATAACTCCAAACCTGTATATACTTAACAACATATACCCAAA

ATATATGAGGCAAATTTTAACAAAAATTGAAGTTCTTAAGAAAACTTCACCAATACATAG

TCATAATGTGGTATATTCATACAACACACTCAATAATTGATAATAACAGTCCAAAGAAAG

CAGAAAGAAGGAAAGCCTAGAGCAGAAATAAATGAAATAGAGAATAGAAAAGCAATAAAG

AAAAAAATTATGAAATCAAACCTGGTTCTTTGAAAAGATCAACAAAATGGCAAACATTTA

GCTAGATTGCCCACCACCCCCAAAAGACTCAAATTACTAGAATCAGAAACAAAAGTCAAG

ATTTTATTACCAATCTTATAGAAATAAAAATTATTAGAAAGAAATAATATGAATAATTGA

ATGCAAACAAGTTAGATAAATGAAATGGACAAATTCCTAGGAAGACACAGAATACCAAAT

CCGACTCCACAAGAAATAGATGTTGTGAATAGAGCTTGCTGTGGTATGAATGTGTCCCCC

ACAGCTCATGCGTTGGAAACTTAATCCCCAGAGCAACAGCACTGAGAGGTGGGACCTTTA

AGAGGTACTAGGCAGAGCCCTCATGAATGGATTAATGCCATTATTGTGGGAGTGGGTTAG

TTATCTAAGAAGTGGGTTCCCGATAAAAGGATGAGTTCAGCCCTCTTCTTCTCCAAACCT

TTATCCCCATCCCCACCCGTCCCTCTCTCTCTCTCTCTTTCTTTCTTTCTCTCTCTCTCT

CTCTTCCCTCCTCTTCTCCCTTCCTTCTGCCATGGGAGGATGAAATATTAAGGCCCTGGC

CAGTTGTGGCCCCTCCGTCTTCCATCTGAGGCTGGAAGACTTCCCGGCCTTCAGAACTGT

AAGAAGTAAATCTCTCTTCTTTAAAAAAAAAAAAAAAAAAAAAAATTGTTCACTCCCACT

GATCCTAGAAAAAATCAGCTCTCCAAGAAGTAGTAGATCATTGTTACTGGCTATCCGCCT

TAGCAAATAGCAGATGCCTCAGAA

>Human-ARGFXP2-NC_000017

AGAGAGAGACACACCGCGCAGGACTGAAAATGGTCTAAGGGGATTCATGACAGATTTCAG

AAACCATGATGAACAGAATGGCCCCAGAGAATCCCTAGCTAGACTCTTTCATCAACAGGA

ATGATTCCAACATGAAGGTGATACCACCACAGGATCCAGCTAGTCCCAGTTTCACTCTGT

CATCCAAACTGGAGTGCAGTGGTGCAGTCTTGGCTCACTGCAGCCTCAACCTCCCAGGTT

CAAGTGATCCTCCCACCTTAGCCTCCCAAGTATCTGGGACTACAGCAATATGGAGAAGGC

ATCAAGAATGTACTTCATTCACCCGCCAACAGTATAAAGAACTAGAAGCTCTGTTTAGCC

AGACCATGTTCCCAGACAGAAATCTTCAGGAGAAACTAGCTTTGAAACTCAACCTACCAG

AGTCAACAGTAAAGAACCCAGTGTCATGGTAAATTGGAGAGGATGTGAGATGATAACCTG

GCTGTTTTCACTCAGGAGAAGCCTGGAATGCCTTCTCCAAGAACAGACCACAATCTCCCC

TCTTCCCCCACTCCCAGGTTTGGTTTAGGAACCGGCGATTCAAATTGAAGCAGCAGCAGC

AGCAGCAGCAGCAATCAGCAAAGCAACCAAACCAGATACCTTCCATCCAAGAAGAATGTG

CCCACCTCCCCCAGAACATCCCCCAGTGCTTATGCTTTTTCTCCCGTGGTTTCAGATTTC

TACAGCTCCCTTCCACCTCAGCTCTTAGACCCTTCCAATTGGGCATGGAACTCTACCTTC

ACTGAGAGTCCCACAAGTGACTTCCAAATGCAAGATACTCAGTGGGAGAGGCTGGTGGCC

TCAGTTCCTGCTTTGTACTCTGATGCCTATGACATATCCCAAATCATAGAACTGTATAAT

CTTCCTGATGAGAATGAGATATCCAGCTCTTCTTTCCACTGTCTGTATCAGTATCTCTCA

CTCACAAAGTACCAGGTAGGAGGACAGGGTTCCTCTCTCAGCACCTTTGCTGGTCTAGCT

GTAGGCCTATCTCCCACACAAACCTGGCCCAGTATGATAAGGCAAGGCTTTAAAGACTAC

AGTCTAACAGACAGCCTGGAATTCCAGAAAACCTCCAATATGGTAGACTTTGGATTTCTC

TGACTAGAGTACTAATAAATATGGACCATTTAGAAAAGAGGTCTTCTTGCATCTTACACA

TGAAAAAAAAAAAAA

>Chimpanzee-ARGFX

ACGAGGAACAGAAGGGCCCCAGAGAATCCCCAGCCAGACTCTTTCATCAATAGGAATTAT

TCCAACATGAAGGTGATACCACCACAGGATCCAGCTAGTCCCAGTTTCACTCTGTTATCC

AAGCTGGAGTGCAGTGGCGCGGTCTCGGCTTACTGCAGCCTCAACCTCCCAGGTTCAACT

GATTCTCCCACCTCAGCCTCCCGAGTAGCTGGGAATACAGCAATACGGAGAAGGCATAAA

GAACGTACTTCTTTCACCCACCAACAGTATGAAGAGCTAGAAGCTCTGTTTAGCCAGACC

ATGTTCCCAGATAGAAATCTTCAGGAGAAACTAGCTTTGAGACTCGACCTACCGGAGTCA

ACAGTAAAGGTTTGGTTCAGGAACCGGCGATTCAAATTGAAGAAGCAGCAGCAGCAGCAA

TCAGCAAAGCAACGAAACCAGATCCTTCCATCCAAGAAGAATGTGCCCACCTCCCCCAGA

ACATCCCCCAGTCCTTATGCTTTTTCTCCTGTGATTTCAGATTTCTACAGCTCCCTTCCA

TCTCAGCCCTTAGACCCTTCCAATTGGGCATGGAACTCTACCTTCACTGAGAGCTCTACC

AGTGACTTCCAAATGCAAGATACTCAGTGGGAGAGGCTGGTGGCCTCGGTTCCTGCTTTG

TACTCTGATGCCTATGACATATTCCAAATCATAGAACTGTACAATCTTCCTGATGAGAAT

GAGATATCCAGCTCTTCTTTCCACTGTCTGTATCAGTATCTCTCACCCACAAAGTACCAG

GTAGGAGGACAGGGTTCCTCTCTCAGCATCTTTGCTGGTCCAGCTGTAGGCCTATCTCCT

GCACAAACCTGGCCCAATATGACAAGCCAAGGCTTTGAAGCCTACAGTCTAACAGATAGC

CTGGAATTCCAGAAAACCTCCAATATGGTAGACTTGGGATTTCTCTGA

>Chimpanzee-ARGFXP1

TTCTTAGAAGAAATGTCTATCCACACATTTGCCCATTGTTTAATTGGGTTATTTGTTCTT

TTATTGTTGAGTTGGATACGGATGTTATTTAAAAATTTTTGGACACACTAAGGAAACTGC

ATTTCCAGAGAGAGACACACCATGCAGGACTGAAAATGGTTAGTCTAAGGGGATTCATGA

CAGATTTCAAAAACCATGATGAACAGAATGGCCCCAGAGAATTTCCAGCCAGACCCTTTC

ATCAACAGGAATGATTCCAACATGAAGGTGATACCACCACAGGATcCAGCTAGTCCCAGT

TTCAGTCTGTCATCCAAGCTGGAGTaCAGTaGCATGGTCTCCACTCACTGCAGCCTCAAC

CTCCCAGGTTCAAGTGATCTTCCCACCTCAGCCTCCCGAGTAGCTGGGACTACAaCAATA

TGGAGAAGGCATCAAGAACGTACTTCATTCACCCACCAACAGTATGAAGAGCTAGAAGCT

CTGTTTAGCCAGACTATGTTCCCAGATAGAAATCTTCAGGAGAAACTAGCTTTGcAACtC

AACCTACTGGAGTCAACAGGTAAAGGCTTGGTTCAGGAACTGGCAATTCAGATTGAAGCA

GCAGCAGCAGCAGCAGCAGCAAtCAAACCAGATCCTTTCATCCAAGAAGAATGTGCCCAC

CTCCCCTAGAACATCCCCAAGTCCTTATGCTTTTTCTCCTGTGGTTTCAGATTTCTACAG

CTCCCTTTCTCCTCAGCCCTTAGACCCTTCCAATTcGGCCTGGAACTCCACCTTCACTGA

GAGTCCCACAGGTGACTTCCAAATGCAAGATACTCAATGGGAGAGGCTGGTGGCCTCAGT

TCCTGCTTTGTACTCTGATGCCTGTGACATATCCCAAATCATAGAACTGTACAATCTTCC

TGATGAGAATGAGATATCCAGCTCTTCTTTCCACTGTCTGTATCAGTATCTCTCACCCAC

AAGGTACCAGGTAGGAGGACAGGGTTCCTCTCTGAGCACCTTTGCTGGTCCAGCTGTAGG

CCTATCTCCCACACAAACCTGgCCCAGTATGACAAGCCAAAGCTTTGAAGCCTACAGTCT

AACAGACAGCCTGGAATTCCAGAAGACCTCCAATATGGTAGACTTTGGATTTCTCTGACC

AGAGTACTAATAAATATGGACCATTTAGAAAAGAGGTCTTCTTGCCTCTTGTACATGACT

TTTTTtTCCTTTGTCTCATTTTAACGCAAACATCTGGGGCTGTGTCTCTGATTTCCATGG

AAATGTTGCAAAAAGATTTcTCCAAGTAGAGCTGGGCACTATGCAATCAGCCCCACAATT

CTTCCTGAGAAGCCTTCCTAGTCCCTTACATGGCCAATGAGACTCCaAAATTCCCTTCCC

AAAATTATCCTGgATTTTCTAAAAGTAGAACAGTGGTTTTGGGAGTTATCCACTTTATTT

TTAAAAAACTAGGTCTCTCTCTGTCACCCAGGCTGGAATGCAGTGGAACAATCATGGCTC

ACCAGAGCCTTGACCTCCTGGGCTCAGGTGATTCTCCCACCTCAGCCTCCTGAGTAGCTG

GGACTACAGGTGCCCGCCACCACACTCGACTAATTTTTTGTTTTTTTGTTTGTTTGTTTT

TTGAGACAGAGTTTCACACTGTCACCCgGGCTGGAGTGCAGTGGCGGGATCTCGGCTCAC

TGCAACCTCCACCTCCCAAGGTTCAGGCGATTCTCCTGCCTCAGCCCCCTGAGCGGCTGG

GATTACAGGTGCATGACACCATGCCCGGCTAATTTTTGTGTTTTTGGTGGAGACAGGGTT

TCACCGTGTTGCCCAGGCTGGTCTCGAACTCCTGACCTCCAGTGATCCACCcaCCTTGAC

CTCCCAGGGTGTGGGATTGCAGGCgTGAGCCACCCcGCCCGGCCAATTTTTGTATTTTTT

GTAgAGGCAAAgTTTTGCCATGTTGCCCaGGCTGGTCTCGAACTCCTGGACTTAACgTCA

TCCACCTGCCTCAGCCTCCCAAAGTGCTAGGAATACAGGAGTGAACCATCGCACCCAGCT

GGGTTATCCAGTTTAAAAGAAaTTTTTTTTCAAGGAAACCAGAGAAGCCATCATCCACTT

TTCTGTCTGTATCATTCCaATTTTAGTATATGTGTTGCgGAAGTGAGTATACACTATCAT

CCACTTTTcATGAGAGgAAGTTATAAACTTACATTTTGTTTCCAATTTTTAGGtTTTTTg

TTTTTTgTTTTTTTTTTTGAGACAGCACTCTGCCTCCAGGCgGGAGTGTGCAGTGGCACC

ATCTTGGCTCACTGCAGCCTCTGCCTCCTGGTCTCTAACAATCCTCCCACCTCAGCCTTC

TAAGTGGCTGGGACTACAGATGAGCGCCACCATGCCTaGCTAATTTTcGTATTTTTAGTG

GAGAAGGGGTTTCGCCATGTTGCCCCAGCTGAATTTGGGATTTTTTaAAAGGGGACTTTC

AGTCCAAATTTGAGGGGAAATTACTATCACCACTAGTTTGAATATAGTACAATGAAAAAA

ATATATATATAGTACAATGGTCCAGACATAATAAGAGGTGTAAAAACTAGCACAGGAAGA

AATAAAAACAGATTTTTGAAGGTGAGATGAtAAACTCACAAAATTAACAAAATAAATAGt

CAACTTTTAGAATGACATTTTAGCACATTTTCCAGCCACATGATCAATCTACAAAATTAA

TATcATTTCTGAACACCAGTAATAAACATAAGGTAAGTACCTCTCAATAAAAAATAGAAA

AGTAcCTAGGAATTCACAAAGAAAATTAATACCTCTGTGGAGAAAACTTCAAAACTTAAA

AAAAGACATAGAAAATGTTTTGAATATGCTGGGcGCAGTGGCTCACACCTGTGGTCCTGG

CACTTTGGGAGcCCAAGGCAGGTGGATCATGGGGTCGGAGACCAAGACCATCCTGGCCAG

AATGGTGAAgCCCtGTCTCTACTAAAAATACCAAATAAATTAGCTGGGAGTGGTGGCACA

TGCCTGTGGTCCCAGCTACTCgGGAGGCTGAGGCAGGAGAATCgCAAGGATTGAAATTAT

GCAGTGTGTTCTCCAAACACGATGGAATGAAAcTAGAAATCAATAGTGGAAGAAAATTTG

GGAAATTCACACATATGTGGATATTAAACAACACATTCCTAAATAACCAATGGTTAAAGG

GGAAATCACAGTCTGAGCAACATGGCAAAACCCTGACTCTGCAGAAAATGGAAAATTAGC

TGGGCATGGCAGCCAGAGCCCAtGCTCCCAGCTACTCAAGAAGCTGAGGTGAGAGgATCT

ATCCCCTGAGCCACAGAGGTCGAGGCTGCAGTGAGCAGTAACTGTGCCACTGCACTCCAG

TTTgAGCAACACAGTGATACCATCTCAATAAATAAATAAATAAAAGAAATCATAAaTAAA

ATTGGAAAACACTTCTAACTGAAAAAAAATAAAAACAGAGCACAACAAACCAAAACATGG

GATGAAGCTAAATCAATGCTTAGAAGGAAATTTaTAGCTTCAAATACTTATATATtTTTT

TAAAAgGGTCTCAGATCAATAAGTTTTATACAGGAAaGAAGCTGGCATAGTTTTATTAAT

ATTTGTCAAATTAAACTTTAAGGCAAAACTTTTTATTGAGATAGAGCCTTGCTCTGTCaC

CCAGGCTGGAGTGCAGTAGTGCAATCTTGaCTCACTGCAACCTTCGCCTCCAGtGTTCAA

ACAATTCTaCTGCCTTAaCCTCCCAGGTGGCTGGGACTACAGGTGTGAGCCACCATGCCt

GGCTAGTTTTTGTATTTTTAGTAGAGACGGGGTTTTGCCATGTTGCTCAGGCTGGCCTCA

AACTCCTGAGCTCAAGTGATCCTCCCACCTCAGCCTCCCAAAGGGCTGAGATTGCAGGTG

TGAGCCACCTTGCCTGGCCAAAACAGTTTTTTAGAAATAAAGTaTATTAGaCAACAAGAA

AAGGAACAATTAACCTGAAATATATGATAACTCCAAACCTGTATATACTTAACAACATAT

ACCCAAAATATATGAGGCAAATTTTAACAAAAATTGAAGTTCTTAAGAAAACTTCACCAA

TACATAGTCATAATGTGGTATATTCATACAACACACTCAATAATTGATcATAACAGTCCA

AAGAAAGCAGAAAGAAGGAAAGCCTAGAGCAGAAATAAATGAAATAGAGAATAGAAAAGC

AATAAAGAAAAAAATTATGAAATCAAAagCTGGTTCTTTGAAAAGATCAACAAAATGGCA

AACATTTAGCTAGATTGCCCACCACtCCAAAAGACTCAAATTACTAGAATCAGAAACAAA

AGTCAAGATTTTATTACCAATCTTATAGAAATAAAAATTATTAGAAAGAAATAATATGAA

TAATTGAATGCAAACAAGTTAGATAActtaaATGAAATGGACAAATTCCTAGGAAGACAC

AGAATACCAAATCCaACTCCACAAGAAATAGATGTTGTGAATAGAGCTTGCTaTGGTATG

AATGTGTCCCCCACAGCTCATGCGTTGGgAACTTAATCCCCAGAGCAACAGCACTGAaAG

GTGGGACCTTTAAGAGGTACTAGGCAGAGCCCcCATGAATGGATTAATGCCATTATTGTG

GGAGTGGGTTAGTTATCTAAGAAGTaGGTTCCCGATAAAAGGATGAGTTCAGCCCTCTTC

TTCTCCAAACCTTTATCCCCATCCCCACCCGTCCCTCTCTCTCTCTCTTTCTTTCTCTCT

CTCTCTCTCTTCCCTCCTCcTCTCCCTTCCTTCTGCCATGGGAGGATGAAATATTAAGGC

CCTGGCCAGaTGTGGCCCCTCCGTCTTCCATCTGAGGCTGGAAGACTTCCCGGCCTTCAG

AACTGTAAGAAaTAAATCTCTCTTCTTTAAAAAAAAAAAAAAAAAAAATTGTTCACTCCC

ACTGATCCTAGAAgAAATCAGCTCTCCAAGAAGTAGTAGATCATTGTTACTtGCTATCCa

CCTTAGCAAATAGCAGATGCCTCAGAA

>Chimpanzee-ARGFXP2

AGAGAGAGACACACCGCGCAGGACTGAAAATGGTCTAAGGGGATTCATGACAGATTTCAG

AAACCATGATGAACAGAATGGCCCCAGAGAATCCCTAGCTAGACTCTTTCATCAACAGGA

ATGATTCCAACATGAAaGTGATACCACCACAGGATCCAGCTAGTCCCAGTTTCACTCTGT

CATCCAAACTGGAGTGCAGTGGTGCAGTCTTGGCTCACTGCAGCCTCAACCTCCCAGGTT

CAAGTGATCCTCCCACCTTAGCCTCCCAAGTATCTGGGACTACAGCAATATGGAGAAGGC

ATCAAGAATGTACTTCATTCACCCGCCAACAGTATAAAGAACTAGAAGCTCTGTTTAGCC

AGACCATGTTCCCAGACAGAAATCTTCAGGAGAAACTAGCTTTGAAACTCAACCTACCAG

AGTCAACAGTAAAGAACCCAGTGTCATGGTAAATTGGAGAGGATGTGAGATGATAACCTG

GCTGTTTTCACTCAGGAGAAGCCTGGAATGCCTTCTCCAAGAACAGACCACAATCTCCCC

TCTTCCCCCACTCCCAGGTTTGGTTTAGGAACCGGCGATTCAAATTGAAGCAtCAGCAGC

AGCAGCAGCAGCAATCAGCAAAGCAACCgAACCAGATACCTTCCATCCAAGAAGAATGTG

CCCACCTCCCCCAGAACATCCCCCAGTGCTTATGCTTTTTtTCCCGTGGTTTCAGATTTC

TACAGCTCCCTTCCACCTCAGCTCTTAGACCCTTCCAATTGGGCATGGAACTCTACCTTC

ACTGAGAGTCCCACAAGTGACTTCCAAATGCAAGATACTCAGTGGGAGAGGCTGGTGGCC

TCAGTTCCTGCTTTGTACTCTGATGCCTATGACATATCCCAAATCATAGAACTGTATAAT

CTTCCTGATGAGAATGAGATATCCAGCTCTTCTTTCCACTGTCTGTATCAGTATCTCTCA

CTCACAAAGTACCAGGTAGGAGGACAGGGTTCCTCTCTCAGCACCTTTGCTGGTCTAGCT

GTAGGCCTATCTCCCACACAAACCTGGCCCAGTATGATAAGGCAAGGCTTTAAAGACTAC

AGTCTAACAGACAGCCTGGAATTCCAGAAAACCTCCAATATGGTAGACTTTGGATTTCTC

TGACcAGAGTACTAATAAATATGGACCATTTAGAAAAGAGGTCTTCTTGCATCTTACACA

TGAAAAAAAAAAAAAAA

>Gorilla-ARGFX

ACGAGGAACAGAATGGCCCCAGAGAATCCCCAGCCAGACCCTTTCATCAATAGGAATTAT

TCCAACATCAAGGTGATACCACCACAGGACCCAGCTCGTCCCACAATACGGAGAAGGCAT

AAAGAACGTACTTCTTTCACCCGCCAACAGTGTGAAGAGCTAGAAGCTCTGTTTAGCCAG

ACCATGTTCCCAGATAGAAATCTTCAGGAGAAACTAGCTTTGAGACTCGACCTACCGGAG

TCAACAGTAAAGGTTTGGTTCAGGAACCGGCGATTCAAATTGAAGAAGCAGCAGCAGCAG

CAGCAGCAGCAATCAGCAAAGCAACGAAACCAGATCCTTCCATCCAAGAAGAATGTGCCC

ACCTCCCCCAGAACATCCCCCAGTCCTTATGCTTTTTCTCCTGTGATTTCAGATTTCTAC

AGCTCCCTTCCATCTCAGCCCTTAGACCCTTCCCATTGGGCATGGAACTCTACCTTCACT

GAGAGTTCTACCAGTGACTTCCAAATGCAAGATACTCAGTGGGAGAGGCTGGTGGCCTCG

GTTCCTGCTTTGTACTCTGATGCCTATGACATATTCCAAATCATAGAACTGTACAGTCTT

CCTGATGAGAATGAGACATCCAGCTCTTCTTTCCACTGTCTGTATCAGTATCTCTCACCC

ACAAAGTACCAGGTAGGAGGACAGGGTTCCTCTCTCAGCATCTTTGCTGGTCCAGCTGTA

GGCCTATTTCCTGCACAAACCTGGCCCAATATGACAAGCCAAGGCTTTGAAGCCTACAGT

CTAACAGATAGCCTGGAATTCCAGAAAACCTCCAATGTGGTAGACTTGGGATTTCTCTGA

>Gorilla-ARGFXP1

TTCTTAGAAGAAATGTCTATCCACACATTTGCCCATTGTTTAATTGTGTTATTTGTTCTT

TTATTGTTGAGTTGGATACGGATGTTGTTTAAAAATTTTTGGACACACTAAGGAAACTGC

ATTTCCAGAGAGAGACACACCATGCAGGACTGAAAATGGTTAGTCTAAGGGGATTCATGA

CAGATTTCAAAAACCATAATGAACAGAATGGCCCCAGAGAATTTCCAGCCAGACCCTTTC

ATCAACAGGAATGATTCCAACATGAAGGTGATACCACCACAGGATCCAGCTAGTCCCAGT

TTCAGTCTGTCATCCAAGCTGGAGTGCAGTGGCATGGTCTCCACTCACTGCAGCCTCAAC

CTCCCAGGTTCAAGTGATCTTCCCACCTCAGCCTCCTGAGTAGCTGGGACTACAACAATA

TGGAGAAGGCATCAAGAATGTACTTCATTCACCCACCAACAGTATGAAGAGCTAGAAGCT

CTGTTTAGCCAGACTATGTTCCTAGATAGAAATCTTCAGGAGAAACTAGCTTTGAAACTC

AACCTACTGGAGTCAACAGTAAAGGCTTGGTTCAGGAACTGGCAATTCAGACTGAAGCAG

CAGCAGCAGCAATCAGCAAAGCAAGCAAACCAGATCCTTTCTTCCAAAAAGAATGTGCCC

ACCTCCCCTAGAACATCCCCAAGTCCTTATGCTTTTTCTCGTGTGGTTTCACATTTCTAC

AGCTCCCTTTCTCCTCAGCCCTTAGACTCTTCCAATTGGGCCTGGAACTCCACCTTCACT

GAGAGTCCCACAAGTGACTTCCAAATGCAAGATACTCAATGGGAGAGGCTGGTGGCCTCA

GTTCCTGCTTTGTACTCTGATGCCTGTGACATATCCCAAATCATAGAACTGTACAATCTT

CCTGATGAGAATGAGATATCCAGCTCTTCTTCCCACTGTTTGTATCAGTATGTCTCACCC

ACAAGGTACCAGGTGGGAGGACAGGGTTCCTCTCTGAGCACCTTTGCTGGTCCAGCTGTA

GGCCTATCTCCCACACAAACCTGGCCCAGTATGATAAGCCAAAGCTTTGAAGCCTACAGT

CTAACAGACAGCCTGGAATTCCAGAAGACCTCCAATATGGTAGACTTTGGATTTCTCTGA

CCAGAGTACTAATAAATATGGACCATTTAGAAAAGAGGTCTTCTTGCCTCTTGTACATGA

CTTTTTTTTCCTTTGTCTCATTTTAACGCAAACATCTGGGGCTGTGTCTCTGATTTCCAT

GGAAATGTCGCAAAAAGATTTTTCCAAGTGGAGCTGGGCACTATGCATTCAGCCCCACAA

TTCTTCCTGAGAAGCCTTCCTAGTCCCTTACATGGCCAATGAGACTCCAAAATTCCCTTC

CCAAAATTATCCTGGATTTTCTAAAAGTAGAACAGTGGTTTTGGGAGTTATCCACTTTAT

TTTTTAAAAACTAGGTCTCTCTCTGTCACCCAGGCTGGAATGCAGTGGAACAATCATGGC

TCACCAGAGCCTTGACCTCCTGGGCTCAGGTGATTCTCCCACCTCAGCCTCCTGAGTAGC

TGGGACTACAGGTGCCCGCCACCACACTCGACTAATTTTTTGTTTTTTTGTTTGTTTGTT

TTTTGAGACAGAGTTTCACACTGTCACCTGGGCTGGAGTGAAGTGGCGGGATCTTGGCTC

ACTGCAACCTCCACCTCCCAAGGTTCAGGCGATTCTCCTGCCTCAGCCCCCTGAGCGGCT

GGGATTACAGGTGCATGACACTATGCCTGGCTAATTTTTGTGTTTTTGGTGGAGACAGGG

TTTCACCATGTTGCCCAGGCTGGTCAAAAAAAAAAAAAAAAAAAAAAAAAAAAAAAAAAA

AAAAAAAAAAAAAAAAAAAAAAAAAAAAAAAAAAAAAAAAAAAAAAAAAAAAAAAAAAAA

AAAAAAAAAAAAAAAAAAAAAAAAAAAAAAAAAAAAAAAAAAAAAAAAAAAAAAAAAAAA

AAAAAAAAAAAAAAAAAAAAAAAAAAAAAAAAAAAAAAAAAAAAAAAAAAAAAAAAAAAA

AAAAAAAAAAAAAAAAAAAAAAAAAAAAAAAAAAAAAAAAAAAAAAAAAAAAAAAAAAAA

ACAAAACCTAAAAATTGGAAACAAAATGTAAGTTTATAACTTTCTCTCATTAAAAGTGGA

TGATAGTGTATACTCACTTCCACAACACATATACTAAAATCGGAATGATACAGACAGAAA

AGTGGATGATGGCTTCTCTGGTTTCCTTGAAAAAAAAAATTCTTTTAAACTGGATAACCC

AGCTGGGTGCGATGTTCACTCCTGTATTCCTAGCACTTTGGGAGGCTGAGGCAGGTGGAT

GACGTTAAGTCCAGGAGTTCGAGACCAGCCTGGGCAACATGGCAAAACCCCATCTCTACT

AAAAATACAAAATTAGCCAGGGTGGTGGCGCATGCCTGTAATCCTAGCTACTAGGGAGGC

TGAGGCAGGAGAATCGCGGCTGAGGCAGGAGAATCGCAAGGATTGAAATTATGCAGTGTG

TTCTCCAAACACGATGGAATGAAATTAGAAATCAATAGTGGAAGAAAATTTGGGAAATTC

ACACATATGTGGATATTAAACAACACATTCCTAAATAACCAGTGGTTAAAGGGGAAATCA

CAGTCTGAGCAACATGGCAAAACCCTGACTCTGCAGAAAATGGAAAATTAGCTGGGCATG

GCAGCCAGAGCCCATGCTCCCAGCTACTCAAGAAGCTGAGGTGAGAGGATCTATCCCCTG

AGCCACAGAGGTCGAGGCTGCGGTGAGCAGTAACTGTGCCACTGCACTCCAGTTTGAGCA

ACACAGTGATACCATCTCAATAAATTAAATAAATAAATAAATAAATAGAAGAAATCATAA

GTAAAATTGGAAAACACTTCTAACTGAAAAAAAATAAAAACAGAAGCACAACAAACCAAA

ACATGGGATGAAGCTAAATCAATGCTTAGAAGGAAATTTATAGCTTCAAATACTTATATA

TATTTTTTAAAAAGGTCTCAGATCAATAAGTTTTATACAGGAAGGAAGCTGGCATAGTTT

TATTAATATTTGTCAAATTAAACTTTAAGGCAAAACTTTTTTATTGAGATAGAGCCTTGC

TCTGTCACCCAGGCTGGAGTGCAGTAGTGCAATCTTGGCTCACTGCAACCTTCGCCTCCA

GGGTTCAAACGATTCTCCTGCCTTAGCCTCCCAGGTGGCTGGGACTACAGGTGTGAGCCA

CCATGCCCGACTGGTTTTTGTATTTTTAGTAGAGACGGGGTTTTGCCATGTTGCTCAGGC

TGGCCTCAAACTCCTGAGCTCAAGTGATCCTCCCACCTCAGCCTCCCAAAGGGCTGAGAT

TGCAGGTGTGAGCCACCTTGCCTGGCCAAAACAGTTTTTTAGAAATAAAGTATATTAGGC

AACAAGAAAAGGAACAATTAACCTGAAAAATATGATAACTCCAAACCTGTATATACTTAA

CAACATATACCCAAAATATATGAGGCAAATTTTAACAAAAATTGAAGTTCTTAAGAAAAC

TTCACCAATACATAGTCATAATGTGGTATATTCATACAACACACTCAATAATTGATAATA

ACAGTCCAAAGAAAGCAGAAAGAAGGAAAGCCTAGAGCAGAAATAAATGAAATAGAGAAT

AGAAAAGCAATAAAGAAAAAAATTATGAAATCAAAACCTGGTTCTTTGAAAAGATCAACA

AAATGGCAAACATTTAGCTAGATTGCCCACCACCCCCAAAAGACTCAAATTACTAGAATC

AGAAACAAAAGTCAAGATTTTGTTACCAATCTTATAGAAATAAAAATTATTAGAAAGAAA

TAATATGAATAATTGAACGCAAACAAGTTAGATAACTTAAATGAAATGGACAAATTCCTA

GGAAGACACAGAATACCAAATCCGACTCCACAAGAAATAGATGTTGTGAATAGAGCTTGC

TGTGGTATGAATGTGTCCCCCACAGCTCATGCGTTGGAAACTTAATCCCCAGAGCAACAG

CACTGAGAGGTGGGACCTTTAAGAGGTACTAGGTAGAGCCCTCATGAATGGATTAATGCC

ATTATTGTGGGAGTGGGTTAGTTATCTAAGAAGTAGGTTCCCGATAAAAGGATGAGTTCA

GCCCTCTTCTTCTCCAAACCTTTATCCCCATCCCCACCCGTCCCTCTCTCTCTCTCTTTC

TTTCTCTCTCTCTCTCTCTCTCTTCCCTCCTCCTCTCCCTTCCTTCTGCCATGGGAGGAT

GAAATATTAAGGCCCTGGCCAGATGTGGCCCCTCCGTCTTCCATCTGAGGCTAGAAGACT

TCCCGGCCTTCAGAACTGTAAGAAATAAATCTCTCTTCTTTAAAAAAAAAAAAAAAAAAA

AAAATTGTTCACTCCCACTGATCCTAGAAAAAATCAGCTCTCCAAGAAGTAGTAGATCAT

TGTTACTTGCTATCCACCTTAGCAAATAGCAGATGCCTCAGAA

>Gorilla-ARGFXP2

AGAGAGAGACACACCGCGCAGGACTGAAAACGGTCTAAGGGGATTCACGACAGATTTCAG

AAACCATGATGAACAGAATGGCCCCAGAGAATCCCTAGCTCGACTCTTTCATCAACAGGA

ATGATTCCAACATGAAGGTGATACCACCACAGGATCCAGCTAGTCCTAGTTTCACTCTGT

CATCCAAACTGGAGTGCAGTGGTGCAGTCTTGGCTCACTGCAGCCTCAACCTCCCAGGTT

CAAGTGATCCTCCCACCTTAGCCTCCCAAGTATCTGGGACTACAGCAATATGGAGAAGGC

ATCAAGAATGTACTTCATTCACCCGCCAACAGTATAAAGAACTAGAAGCTCTGTTTAGCC

AGACCATGTTCCCAGACAGAAATCTTCAGGAGAAACTAGCTTTGAAACTCAACCTACCAG

AGTCAACAGTAAAGAACCCAGTGTCATGGTAAATTGGAGAGGATGTGAGATGATAACCTG

GCTGTTTTCACTCAGGAGAAGCCTGGAATGTTCTCCAAGAACAGACCACAATCTCCCCTC

TTCCCCCACTCCCAGGTTTGGTTTAGGAACCGGCGATTCAAATTGAAGCATCAGCAGCAG

CAAGCAGCAGCAATCAGCAAAGCAACCGAACCAGATACCTTCCATCCAAGAAGAATGTGC

CCACCTCCCCCAGAACATCCCCCAGTGCTTATGCTTTTTCTCCCGTGGTTTCAGATTTCT

ACAGCTCCCTTCCACCTCAGCTCTTAGACCCTTCCAATTGGGCATGGAACTCTACCTTCA

CTGAGAGTCCCACAAGTGACTTCCAAATGCAAGATACTCAGTGGGAGAGGCTGGTGGCCT

CAGTTCCTGCTTTGTACTCTGATGCCTATGACATATCCCAAATCATAGAACTGTATAATC

TTCCTGATGAGAATGAGATATCCAGCTCTTCTTTCCACTGTCTGTATCAGTATCTCTCAC

TCACAAAGTACCAGGTAGGAGGACAGGGTTCCTCTCTCAGCACCTTTGCTGGTCTAGCTG

TAGGCCTATCTCCCACACAAACCTGGCCCAGTATGATAAGGCAAGGCTTTAAAGACTACA

GTCTAACAGACAGCCTGGAATTCCAGAAAACCTCCAATATGGTAGACTTTGGATTTCTCT

GACCAGAGTACTAATAAATATGGACCATTTAGAAAAGAGGCCTTCTTGCATCTTACACAT

GAAAAAAAAAAA

>Orangatun-ARGFX

ATGATGAACAGAATGGCCCCAGAGAATCCCCAGCCAGACCCTTTCATCAAAAGGAATTAT

TCCAACATGAAGGTGATACCACCACAGGATCCAGCTAGTCCCAGTTTCACTATGTTATCC

CAGCTGGAGTGCAGTGGCACAGTCTCGGCTTACTGCAGCCTCAACCTCCCAAGTTCAACT

GATCCTCCCACCTCAGCCTCCCGAATAGCTGGGAATAGAGCAATACGGAGAAGGCATCAA

GAACGTACTTCTTTCACCCACCAACAGTATGAAGAGCTAGAAGCTCTGTTTAGCCAGACC

ATGTTCCCAGATAGAAATCTTCAGGAGAAACTAGCTTTGAGACTCGACCTACCGGAGTCA

ACAGTAAAGGTTTGGTTCAGGAACCGGCGATTCAAATTGAAGAAGCAGAAGCAGCAGCAG

CAGCAGCAGCAGCAGCAGCAGCGGCAATCAGCAAAGCAACGAAACCAGATCCTTCCATCC

AAGAAGAATGTGCCCACTTCCCCCAGAACATTCCCCAGTCCTTATGCTTTTTCTCCTGTA

ATTTCAGATTTCTACAGCTCCCTTCCACCTCAGCCCTTAGACCCTTCCAATTGGGCATGG

AACTCTACCTTCACTGAGAGTTCCACAAGTGACTTCCAAATGCAAGATACTCAGTGGGAG

AGGCTGGTGGCCTCGGTTCCTGCTTTGTACTCTGATGCCTATGACATATTCCAAATCATA

GAACTGTACAATCTTCCTGATGAGAATGAGATATCCAGCTCTTCTTTCCACTGTCTGTAT

CAGTATCTCTCACCCACAAAGTACCAGGTAGGAGGACAGGGTTCCTCTCTCAGCACCTTT

GCTGGTCCAGCTGTAGGCCTATCTCCCGCACAAACCTGGCCCAGTATGACAAGCCAAGAC

TTTGAAGCCTACAGTCTAACAGATAGCCTGGAATTCCAGAAAACCTCCAATATGGTAGAC

TTGGGATTTCTCTCA

>Orangatun-ARGFXP1

TTCTTAGAAGAAATGTCTATtCACACcTTTGCCCATTGTTTAATTGGGTTATTTGTTCTT

TTATTGTTGAGTTGGATACaGATGTTATTTAAAAATTTTTGGACACACTAAGGAAACTGC

ATTTCCAGAGAGAGACACACCATGCAGGACTGAAAATGGTTgGTCTAAGGGGATTCATGA

CAGATTTCAgAAACCATGATGAACAGAATGGCCCCAGAGAATTTCCAGCCAGACCCTTTC

ATCAACAGGAATGATTCCAACATGAAGGTGATAgCACCACAGGATcCAGCTAGTCCCAGT

TTCAGTCTGTCATCCAAGCTGGAGTGCAGTGGCATGGTCTCCACTCACTGCAGCCTCgAt

CTCCCAGGTTCAAGTGATCTTgCCACCTCAGCCTCCCGAaTAGCTGGGACTACAaCAATA

TGGAGAAGGCATCAAGAACGTACTTCATTCACCCACCAACAGTATGAAGAGCTAGAAGCT

CTGTTTAGCCAGACTAcaTTCCCAGATAGAAATCTTCAGGAGAAACTAGCTTTGAAACtC

AACCTACTGtAGTCAACAGTAAAaGCTTGGTTCAGGAACTGGCAATTCAGATTGAAGCAG

CAGCAGCAGCAGCAGCAGCAGgagCAGCAATCAGCAAAGCAAGCAAACCAGATCCTTTCA

TCCAAGAAGAATGTGCCCAtCTCCCCTAGAACATCCCCAAGTCCTTATGCTTTTTCTCCT

GTGGTTTCAGATTTCTACAGCTCCCTTcCTCCTCAGCCCTTAcACCCTTCCAATTGGaCC

TGGAACTCCACCTTCACTGAGAGTCCCACAaGTGACTTCCAAATGCAAGATACTCAATGG

GAGAGGCTGGTGGCCTCAGTTCCTGCTTTGTACTCTGATGCCTaTGACATATCCCAAATC

ATAGAACTGTACAATCTTCCTGATGAGAATGAGATATCCAGCTCTTCTTTCCACTGTCTG

TATCAGTATCTCTCACCCACAAGGTACCAGGTAGGAGGACAGGGTTCCTCTCTGAGCACC

TTTGtTGGTCCAGCTGTAGGCCTATCTCCCACACAAAaCTGgCCCAGTATGACAAGCCAA

gGCTTTGAAGCCTACAGTCTAACAGCCTGGAATTCCAGAAGACCTCCAATATGGTAGACT

TTGGATTTCTCTGACCAGAGTAtTAATAAATATGGACCATTTAGAAAAGAGGTCTTCTTG

CCTCTTGTACATGACTTTTTTttTCCTTTGTCTCATTTTAACGCAAACATCTGGGaCTGT

GTCTCTGATTTCCATGGAAATGTTGCAAAAAGAgTTTTCCAAGTAGAGCTGGGCACTATG

CAATCAGCCCCACAATTCTTCCTGAGAAGCCTTCCTAGTCtCTTACATGGCCAATGAGAC

TCCaAAATaCaCTTCCCAAAAcTATCCTgGATTTTCTAAAAGTAGgACAGTGGTTTTGGG

AGTTATCtACTTTATTTTTtAAAAACTAGGTCTCTCTCTGTCACCCAGGCTGGAATGCAG

TGGtACAATCATGGCTCACCAaAGCCTTGACCTCCTGGGCTCAGGTGATTCTCCCACCTC

AGCCTCCTGAGTAGCTGGGACTACAaGTGCCCGCCACCACACTCGgCTAATTTtttgTTT

GTTTTTTcaTTTGTTTGTTTTTTGAGACAGAGTcTCACACTGTCACCCAGGCTGGAGTGC

AaTGGCGGGATCTCaGCTCACTGCAACCTCCACCTCCCgAcGTTCAGGCGATTCTCCTGC

CTCAGCCCCCTGAGCGGCTGGGATTACAGGcGCATGcCACCATGCCCGGCTAATTTTTGT

GTTTTTGGTGGAGACAGGGTTTCACCaTGTTGCCCAGGCTGGTCTCGAACTCCTGACctc

cagtgatccacccgccttgacctcccagggtgtgggattgcaggtgtgagccaccnnnnn

nnnnnnnnnnnnnnnnnnnnnnnnnnnnnnnnnnnnnnnnnnnnnnnnnnnnnnnnnnnn

nnnnnnnnnnnnnnnnnnnnnnnnnnnnnnnnnnnnnnnnnnnnnnnnnnnnnnnnnnnn

nnnnnnnnnnnnnnnnnnnnnnnnnnnnnnnnnnnnnnnnnnnnnnnnnnnnnnnnnnnn

nnnnnnnnnnnnnnnnnnnnnnnnnnnnnnnnnnnnnnnnnnnnnngaactcctgacCTC

CAGTGATCCACCcGCCTTGACCTCCCAGGGTGTGGGATTGCAGGtgTGAGCCACCCAGCC

CaGCCAATTTTTGaATTTTTTGTAgAGGCAAAgTTTTGCCATGTTGCCCaGGCTGGTCTC

GAACTCCTGGACTTAACgTCATCCACCTGCCTCAGCCTCCCAAAGTGCTAGGAATACAaG

AtTGAACCATCaCACCCAGCTGGGTTATCCAGTTTAAAAGAATTTTTTTTTttCAAGGAA

ACCAGAGAAGCCATCATCCACTTTTCTcTCTGTATCATTCCGATTTTAGTATATGTGTTG

CgGAAGTGAGTATACACTATCATCCACTTTTAATGAAAGTTATaAAACTTACATTTTGTT

TCCAATTTTTTTTTTTTTTTTTTTTTTTTGAGACAGCACTCTGCCTCCAGGCTGGAGTGT

GCAGTGGCACCATCTTGGCTCACTGCAGCCTCTGCCTCCTGGgCTCTgACAATCCTCCCA

CCTCAGCCTTCTgAGTGGCTGGGACTACAGATGAGCGCCACCATGCCTaGCTAATTTTTG

TATTTTTAGTGGAGAAGGtGTTTCGCCATGTTGCCCagGCTGAATTTGGGgTTTTTTTAA

AGGGGACTTTCAGTCCAAATTTGAGGGGAAATTACTATCACCACTAGTTTtAATATAGTA

CAATGAAAAAtATATATATATAGTACAATGGTCCAGACATAATAAGAGGTGTAAAAACTA

GCACAGGAAGAAATAAAAACAGATTTTTGAAGGTGAGATGAtAAACTCgCAAAATTAACA

AAATAAATAGtCAAACTTTTAGAATGACATTTTAGCACATTTTCCAGCCACcTGATCAAT

CTACAAAATTAATATcATTTCTGAACACCAGTAATaaagAAACATAAGGTAAGTACCTCT

CAATAAAAAATAGAAAAGTAcCTAGGAATTCACgAAGAAAATTAATACCTCTGTGGAGAA

AACTTCAAAACTTAAAAAAGACATAGAAAATGTTTTGAATATGCTGGGTGCgGTGGCTCA

CACCTGTGGTCCTGGCACTTTGGGAGgCgAAGGCAGGTGGATCATGGGGTCAGGAaAtCA

AGACCATCCTGGCCAGAATGGTGAAgCCCCGTCTCTACTAAAAATACCAAATAAATTAGC

TGGGAGTGGTGGCACATGCCTGTGGTCCCAGCTACTCAGGAGGCTGAGGCAGGAGAATCg

CAAGGATTGAAATTATGCAGTGTGTTCTCCAAACACGATGGAATGAAATTAGAAATCAAT

AGTGGAAGAAAATTTGGGAAATTCACACATATGTGGATATTAAACAACACATTCCTAAAT

AACCAATGGTTAAAGaGGAAATCACAGTCTGAGCAACATGGCAAAACCCTGtCTCTGCAG

AAAAcGGAAAATTAGCTGGGCATGGCAGCCAGAGCCCAtGCTCCCAGCTACTCAAGAgGC

TGAGGTGAGAGgATCTATCCCCTGAGCCACAGAGGTCGAGGCTGCAGTGAGCAGTAACTG

TGCCACTGCACTCCAGcTTgAGCAACACAGTGATACCATCTCAATAAATAAATAAATAAA

TAAATAAATAAgTAGAAGAAATCATAAGTAAAATTGGAAAACACTTCTAACTGAAAAAAA

AAAAAcCAGAAGCACAACAAACCAAAACATGGGATGAAGCTAAATCAATaCTTAGAAGGA

AATTTaTAGCTTCAAATACTTATATATaTTTTTTAAAAAGGTCTCAGATCAATAAGTTTT

ATACAGGAAGGAAGCTGGCATAGTTTTATTAATATTTGcCAAATTAAACTTTAAGGCAAA

ACTTTTTTATTGAGATAGAGCCTTGCTCTGTCGCCCAGGCTGGAGTGCAGTAGTGCAATC

TcGGCTCACTGaAACCTTCGCCTCCAGGGTTCAAACgATTCTtCTGCCTTAGCCTCCCAG

GTGGCTGGGACTACAGGTGTGAaCCACCtcGCCCGGCTAGTTTTTGTATTTTTAGTAGAG

ACaGGGTTTTGCCATGTTGCTCAGGCTGGCCTCAAACTCCTGAGCTCAAGTGATCCTCCt

ACCTCAGCCTCCCAAAGGGCTGAGATTGCAGGTGTGAGCCACCTcGCCTGGCCAAAACAG

TTTTTTAGAAATAAAGTaTATTAGGCAACAAcAAAAGGAACAATTAACCTGAAATATATG

ATAACTCCgAACtTGTATATACTTAACgACATATACCCAAAATATATGAGGCAATTTTAA

CAAAAATTGAAGTTCTTAAGAAAACTTCACCAATACATAGTCATAATGTGGTATATTCAT

AtAACACACTCAATAATTGATAATAACAGTCCAAAGAAAGCAGAAAcAAGGAAAGCCTAG

AGCAGAAATAAATcAAATAGAGAATAGAAAAGCAATAgAGAgAAAAATTATGAAATCAAA

agCTGGTTCTTTGAAAAGATtAACAAAATGGCAAACATTTAGCTAGATTGCCCACCACCC

CCAAAAGACTCAAATTACTAGAATCAGAAACAAAAGTCAAGATTTTATTACCAATCTTAT

AGAAATAAAAATTATTAGAAAGAAATAATATGAAgAATTGAATGCcAACAAGTTAGATAA

cttaagTGAAATGGAtAAATTCCaAGGAAGACACAGAATACCAAATCCGACTCCACAAGA

AATAGATGTTGTGAATAGAGCTTGCTaTGGTATGAATGTGTCCtCCACAGCTCATGCaTT

GGAAACTTAATCCCCAaAGCAACAGCgCTGAGAGGTGGGACCTTTAAGAGGTACTAGGCA

GAGCCCcCATGAATGGATTAATGCCATTgTTGTGGGAGTGGGTTAGTTATCTAAGAAGTG

GGTTCCtGATAAAAGGATGAGTTtgGCCCTCTTCTTCTCCAAAtCTTTATCCCCATCCCC

ACCtGTCCCTCTCTCTCcCTCTCTCTTTCTctctctctctctcTCTCTCTCTCTCTCTCT

TCCCTCCTCcTCTCCCTTCCTTCTGCCATGGGAGGATGAAATATTAAGGCCCTGGCCAta

TGTGGCCCCTCCaTCTTCCATCTGAGGCTGGAAGACTTCCCaGCCTTCAGAACTGTAAGA

AaTAAATCTCTCTTCTTcAAAAAAAAAAAAAATTGTTCACTCCCACTGATCCTAGAAAAA

ATCAGCTCTCCAAGAAGTAGTAGATCATTGTTACTtGCTATCtGCCTTAGCAAAcAGCAG

ATGCtTCAGAA

>Orangatun-ARGFXP2

AGAGAGAGACACACCaCGCAGGACTGAAAATGGTCTAAGGGGATTCATGACAGATTTCAG

AAACCATGATGAACAGAATGGCCCCAGAGAATCCCcAGCTAGACcCTTTCATCAACAGGA

ATGATTCCAACATGAAGGTGATACCACCACAGGATCCAGCTAGTCCCAGTTTCACTCTGT

CATCCAAgCTGGAGTGCAGTGGTGCAGTCTTGGCTCACTGCAGCCTCAACCTCCCAGGTT

CAAGTGATCCTCCCACCTTAGCCTCCCAAGTATCTGGGACTACAGCAATATGGAGAAGGC

ATCAAGAAcGTACTTCATTCACCCGCCAACAGTATgAAGAgCTAGAAGCTCTGTTTAGCC

AGACCATGTTCCCAGACAGAAATCTTCAGGAGAAACTAGCTTTGAAACTCAACCTACCAG

AGTCAACAGTAAAGAACCCAGTGTCATGGTAAATTGGAGAGGAcGTGaAGATGATAACCT

GGCTGTTTTCACTCAGGAGAAGCCTGGAATGCCTTCTCCAAGAACAGACCACAATCTCCC

CTCTTCCCCCACTCCCAGGTTTGGTTTAGGAACCGGCGATTCAAATTGAAGCAtCAGCAG

CAGCAGCAATCAGCAAAGCAACCAAACCAGATACCTTCCAcCCAAGAAGAATGTGtCCAC

CTCCCCCAGAACATCCCCCAGTGCTTATGCTTTTTCTCCCaTGGTTTCAGATTTCTACAG

CTCCCTTgCACCTCAGCTCTTAGACCCTTCCAATTGGGCATGGAACTCTACCTTCACTGA

GAGTCCCACAAGTGACTTCCAAATcCAAGATACTCAGTGGGAGAGGCTGGTGGCCTCAaT

TCCTGCTTTGTACTCTGATGCCTATGACATATCCCAAATCATAGAACTGTATAATCTTCC

TGATGAGAATGAGATATCCAGCTCTTCTTTCCACcGTCTGTATCAGTATCTCTCACTCAC

AAAGTACCAGGTAGGAGGACAGGGTTCCTCTCTCAGCACCTTTGCTGGTCTAGCTGTAGG

CCTATCTCCCACACAAACCTGGCCCAGTATAAGGCAAGGCTTTAAAGACTACAGTCTAAC

AGACAGCCTGGAATTCCAGAAAACCTCCAATATGGTAGACTTTGGATTTCTCTGACaAGA

GTACTAATAAATATGGACCATTTAGAAAAcAGGTCTTGCATCTTAtACATGAAAAAAAAA

AtAA

>Macaque-ARGFX

ATGATGAACAGAATGGCCCCAGGGAATCGCCAGCCAGACCCTTTCATCAACAGGAAAGAT

CCCAACATGAAGATGATACAACTGCAGGATCCAGCTAGTCCCATTTTGCTCTTATTGCCC

AGGCTGGAGTGGAATGGCGCGATCTCGGATCACTGTAACCTACACTTCTTAGAATCAAGT

GATTCTCCTGCCTCAGCCTCCCAAATAGCTGGGATTACAGGCATGCCAACATGGAGAAGG

CATCAAGAACGTACTTCATTCTCCCGCCAACAGTATGAAGCGCTAGAAGCTCTGTTTAGC

CAGACCATGTTCCCAGATAGAAATCTTCAGGAGAAACTAGCTTTGAAACTCAACCTGCCG

GAGTCAACAGTAAAGGTTTGGTTCAGGAACCGGCGATTCAAATTGAAGAAGCAGCAGCAG

CAGCAGCAGCAGCAGCAGCAGCAGCAGCAGCAGCAATCAGCAAAGCAACGAAACCAGATC

CCTTCATCCAAGAAGAATGTGCCCACCTCCCTCAGAACGTCCCTCAATCCTTATGCTTTT

TCTCCTGTGATTTCAGATTTCTACAGCTCCCTTCCACCTCAGCCCTTAGACCCTTCCAAT

AGGGCATGGAACTCTACCTTCACTGAGAGTTCCACAAGTGACTTCCAAATGCAAGATACT

CAGTGGGAGAGGCTGGTGGCCTCAGTACCTGCTTTGTACTCTGATGCCTATGACATATTC

CAAATCATAGAACTGTACAATCTTCCTGATGAGAATGAGATATCCAGCTCTTCTTTCCAC

TGTCTGCATCAGTATCTCTCACCCACAAAGTACCAGATAGAAGGACAGGGTTCCTCTCTC

AGCACCTTTGCTGGTCCAGCTGTAGGCCTATCTCCCGCACAAACCTGGCCCAGTATGACA

AGCCAAGGCTGTCAAGCCTACAGTCaagcnnCAACAGATAGCCTGGAATTTCAGAAAACC

TCCAATATGGTAGACTTGGGACTAGAGTACTAA

>Macaque-ARGFXP1

TTCTTAGAAGAAATGTCTATTCACATCTTTGCCCATTGTTTAATTGGGTTATTTGTTCTT

TTATTGTTGAGTTGGATACGGCTGTTATTTTAAAACTTTTGGATACACTAAGGGAACTGC

ATTTCCAGCGAGAGACACACCACGCAGGACTGAAAATGGTTAGTCTAAGGGGATTCATGA

CAGATTTGAGAAACCATGATGAACAGAATTTCCAGCCAGAGAATTTCCACTCAGACCCAT

CATCAACAGGAATGATTCCAACATGAAGGTGAACCACCACAGGATCCAGTTAGTCCCAGT

TTCACTCTGTCATCCAAGCTGGAGTGCAGTGGCATGGTCTCCACTCACTGCAGCCTCAAC

CTCCCAGCTTCAAGTGATCTTCCCACCTCAGCCTCCCGAGTAGCTGGGACTACAGCAACA

TGGAGAAGGCATCAAAAACGTACTTCGTTCATCCACCAACAGTATGAAGAGCTAGAAGCT

CTGTTTAGCCAGACTGTGTTCCCAGACAGAAACCTTCAGGAGAAACTAGCTTTGAAACTC

AACCTACTGGAGTCAACAGTAAAGGCTTGGTTCAGGAACTGGCAATTCAGATTGAAGCAG

CAGCAATCAGCAAAGCAAGCAAACCAGATCCTTCCATTCAAGAAGAATGTGCCCACCTTC

CCCTGAACACCCCCCAGTCTTTATGCTTTTTCTCCTGTAGTTTCAGATTTCTACAGCTCC

CTTCCACCTCAGCCCTTAGACCCTTCCAACTGGGCCTGGAACTCTACCTTCACTGAAAGT

CCCACAAGTGACTTCCAAATGCAAGATACTCAATGGGAGAGGCTGGTGGCCCCAGTTCCT

GCTTTGTACTCCGATGCCTATGACATATCCCAAATCATAGAACTGTACAATCTTCCTGAT

GAGAATGAGATATCCAGCTCTTCTTTCCACTGTCTGTATCAGTATCTCTCACCCACAAGG

TACCAGGTAGGAGGACAGGATTTCTCTCTCAGCACTTTGCTGGTCCAGCTGTAGGCCTAT

CTCCCACACAAACCTGGCCCAGTATGACAAGCCAAAGCTTTGAAGCCTACAGTCTAACAG

ACAGCCTGGAATTCCAGAAAACCTCCAATACGGTAGACTTTGAATTTCTCTGACCAGAGT

ACTAATAAATATGGACCATTTTAAAAAGAGGTCTTCTTGCCTCTTGTACATAGCTATTGT

TTCCTTTGTTTCGTTTTAACCCAAACATCTGGGTCTGTGTCTCTGATTTCCATGGAAATG

TTGTGAAAAGAGATTTCCAAGTAGAGCTGGGCACTACGCAATCAGCCCCACAATTCTCCC

TGAGAAGCCTTCCTAGTCCCTTACATGGCCAATGAGACTCCAAAATTCCCCTCCCAAACT

ATCCTGGAGTTTCTAAAAGTAGGACAGTGGTTTTCGGGGTTATCCACTTTATTTTTTTAA

AACTAGGAATCACTCTGTCACCCAGGCTGGAATGCAGTGGCACAATCATGGCTCACCAAA

GCCTTGACCTCCTGGGCTCAGGTGATCCTCCCACCTCAGCCTCCTGAGTAGCTGGGACTA

CAAGTGCCCGCCACCACACTCGGCTAATTTTTTGTTTGTTTTTTGTTTTTTGAGACAGAG

TCTCGCACTGTCACCCAGGCTGGAGTGCAGTGGCAGAATCTTGCAGAATCTCGCTGCAAC

CTCCACCTCCCGAGGTTCAGGTGGTTCTCCTGCCTCAGCCCCCTTAGTGGCTGGGATTAC

AGGTGCATGCCACCATGCCCGGATAATATTTGTGTTTTTGGTGGAGACAGGGTTTCACCA

TGTTGCCCAGGCTGGTCTCGAACTCCTGACCTCCAATGATTCGCCCACTTTGACCTCCCA

TGGTGTGGGATTGCAGGCATGAGCCACCGAGCCCAGCCAATTTTTGTATTTTTCGTGGAG

GCAAAGTTTTGCATGTTGCCCAGGCTGGTCTCGAACTCCTGGAGTTAACATCATCCACCT

GCCTCAGCCTCCCAAAGTGCTAGGAATACAGGAGTGAGCCATCGCACCCAGCTGGGTTAT

CCAGTTCAAAAGAATTTTTTTTTTCAAGGTAACCAGAGAAGCCATCATCCACTTTTCTCT

CTGTATCATTCTGATTTTAGTATATGTGTTGTGGAAGTGAGTATACACTATCATCCACTT

TTAATGAGAGAAAGTTATAAACTTACCTTTTGTTTCCAATTTTTAGGGTTTTTTTGTTTG

TTTGTTTTTTGTTTTTTGTTTTGTTTTGTTTTGTTTTGTTTTTGTTTTTGTTTTGGAGAC

AGCACTCTGCTTCCAGGCTGGAGTGCAGTGGCACCATCTTGGCTCACTGCAGCCTCTGCC

TCCTGGGCTCAAACAATCCTCCCACCTCAGCCTTCTGAGTGTCTGGGACTACAAATGCGT

GCCACCATGCCTGGCTAATTTTTGTATTTTTAGTGGAGAAGTGGTTTCGCCATGTTGCCC

AGGCTGAATTTGGGGTTTTTTTAAAGGAGACTTTCAGTCCAAATTTGAGGGGAAATTACT

ATCACCACTAGTTTTAATATAGTATAATGAAAAAATATATATATAGTACAATGGTCCAGA

CATAATAAGAGGTGCAAAAACTAGCACAGGAAGAAATAAAAACAGATTTTTGAAGGTGAG

ATGATAAACTCGCAAAATTAACAAAATAAATAGTCAAACTTTTAGAATGACATTTTAGCA

CATTTTCCAGCCACGTGATCATTCTACAAAATTAATATCATTTCTGAACACCAGTAATAA

AGAAACATAAGGTAAGTACCTCTCAATAAAAAATGTAAAAGTACCTGGGAATTCACGAAG

AAAATTAATACCTCTGTGGAGAAAACTTCAAAAACTCAAAAAAAGACATAGAAAATGTTT

TGAATAGGCCGGGCACAGTGGCTTATGCCTGTGGTCCTGGCACTTTGGGAGGTCAAGGCG

GGTGGATCATGGGGTCAGGAGATCAAGACCATCCTGGCCAGCATGGTGAAGCCTCGTCTC

TACTAAAAGTACCAAATAAATTGGCTGGGCCTGGTGACGCATGCCTGTGGTCCCAGCTAC

TCGGGAGGCTAAGGCAGGAGAATCGCAAGGATTGAAATCACGCAGTGTGTTCTCCAAACA

CGATGGAATGAAATTAGAAATCAATAGTGGAAGAAAATTTGGGAAATTCACAAATATGTG

GATATTAAACAATACATTCCTAAATAACAAATGGTTAAATGGGAAATCACAGTCTGAGCA

ACATGGCAAAACTCTGTCTCTGCAGAAAATGGAAAATTGGCTGGACATGGCAGCCAGAGC

CCATGCTCCCAGCTACTCAGGAGGCTGAGGTGAGAGGATCCATACCCCGAGCCACAGAGG

TCGAGGCTGCAGTGAGCAGTGACTGTGCCACTGCACTCCAGGTTGAGCAACACAGTGATA

CCATCTCAATAAATAAATAAATAGAAGAAATCGTAAGTAAAATTGGAAAACACTTTTAAC

TGAAAAAAAACAAAAACAAAAACAGAAGCACAACAAACCAAAACATGGGATGAAGCTAAA

TCAATGCTAAGAATGAAATTTATAGCTTCAAATACTTACATATATATTTTTAAAAGGTCT

CAGATCAATAAGTTTTATACAGGAAGGAAGCTGGCATAGTTTTACTAATATTTGTCAAAT

TAAACTTTAAGGCAAAACTTTTTTATTGAGATGGAGCCTTGCTCTGTCGCCCAGGCTGGA

GTGCAGTAGTGCGATCTCGGCTCACTGCAACCTTTGCCTCCAGAGTTCAAACGATTCTGC

TGCCTTAGCCTCCCAAGTGGCTGGGACTACAGGTGTATGCCACCATGCCAGGCTAGTTTT

TGTATTTTTAGTAGAGATGGGGTTTTGCCATGTTGCTCAGGCTGGCCTCAAACTCCTGAG

CTCAAGTGATCCTCCTACCCCAGCCTCCCAAAGGGCTGAGATTGCAGGTGTGAGCCACCT

CGCCTGGCCAAAACAGTTTTTTAGAAATAAAGTATATTAGACAACAACAAAAGGAACAAT

TAACCTGAAATATATAACTCCGAACTTGTATATACTTAACATATACTCAAAATATATGAG

GCAAATTTTAACAGAAATTAAAGTTCTTAAGAAAACTTCACCAATACATAGTCATAATGT

GGCATATTCATACAACACTCTCAATAATTGATAATAACAGTCCAAAGGAAGCAGAAAGAA

GGAAAGCCTAGAGTAGAAATAAATGAAATAGAGAATAGAAAAGCAATAGAGAAAAAAATT

ATGAAATCAAAAGCTGGTTCTTTGAAAAGCTCAACAAAATGGCAAACATTTAGCTAGATT

GACCAACCCCCAAAAAGACTCAAATTACTAGAATCAGAAACAAAAGTCAAGATTTTATTA

CCAACCTTATAGAAATAAAAATTTTTAGAAAGAAATAATATGAAGAATTGAACACCAACA

AATTAGATAAATGAAATGGACAAATTCCTAGGAAGACACGGAATACCTCATCTGACTCCA

CAAGAAATAGATGCTGTGAATAGAGCTTGCTATGGTATGAATGTGTCCCCCACAGCTCAC

GCATTGGAAACTTAATCCCCCAAGCAACAGCGCAGAGAGGTGGGGCCTTTANNNNNNNNN

NNNNNNNNNNNNNNNNNNNNNNNNNNNNNNNNNNNNNNNNNNNNNNNNNNNNNNNNNNNN

NTCTCTCTCTCTCTCTCTCTCTCTCTCTCTCTCTCTCTCTCTCTCCCCTCCTCCTCTCCC

TTCCTTTTGCCATGAGAGGATGAAACATGAAGGCCCTGGCCAGATATGGCCCCTCCATCT

TCCATTTGAGGCTAGAAGACTTCCCAGCCTTCAGAACTGTAAGAAATAAACCTCTCTTCT

TTAAAAAAAAAAAAAAAAAATTGTTCACTTCCACTGATCCTAGAAAAAATCAGCTCTCCA

AGAAGGAGTAGATCATTGTTACTTGCTATCTGCCTTAGTAAATAGCAGATGCCTCAGAA

>Macaque-ARGFXP2

AGAGAGAGACACACCACGCAGGACTGAAAATGGTTCGTCTAAGGGGATTCATGACAGATT

TCAGAAACCATGATGAACAGAATGGCCCCAGAGAATCCCCAGCCAGACCCTTTCATCAAC

AGGAATGATTCCAACATGAAGGTGATACCACCACAGGATCCAGATAGTCCCAGTTTCACT

CTGTCATCTAAGCTGGAGTGCAGTGGTGCAGTCTTGGCTCACTGCAGCCTCAACCTCCCA

GGTTCAAGTGATCCTCCCACCTTAGCCTCCAAGAAGGTGGGACTACAGCAATATGGAGAA

AGCATCAAGAACATATTTAATTCACCCGCCAACAGTATGAAGAGCTTAGGAAGTTCTGTT

TAGCCAGACCATGTTCCTAGATAGAAATTTTCAGGAGAAACTAGCTTTGAAACTCAACCT

ACCAGAGTCAAAAGTAAAGAACACAGTGTCATGGTGAATTGGAGAGGACATGAAGATGAT

AACCTGGTTGTTTTCACTCAGGAGAAGCCTGGAATGCCTTCTCCAAGAACAGACCACAAT

CTCCTCTCTTCCTCCACTCCCGGGTTTGGTTTAGGAACCGGTGATTCAAACTGAAGCAGC

AGCAGCAATCAGCAAAGCAATTTTTTTATATACCTTCCATCCAAGAAGAATGTGCCCACC

TCCTTCAGAACATCCCCCAATCCTTATGCTTTTTCTCCTGTTGTTTCAGATTTCTACAGC

TCCCTTCCACCTCAGGCCTTAGACTCTTTCAACTGGGCATGGAACTCTACTTTCACTGAG

AGTCCNNNNNNNNNNNNNNNNNNNNNNNNNNNNNNNNNNNNNNNNNNNNNNNNNNNNNNN

>Marmoset-ARGFX

ATGATGAACAGAATGGCCCCAGAGAATCCCCAGCAAGACCCTTCCATCAACAGGAATGAT

TCCAGCATTAAGGTGATATCACCACAGGATCCCGCTAGATATCAGCAGGAAGGTTTCATT

CTGTCATCCCAGCATGAGTGTGGTGGCGCAGTCTCGGCTCACTGCAGCCTCAACCTCCCA

GGCTCAAGTGATCCTCCCGCCTCAGCCTCCCAAGCAACTGGGACTACAGCAACATGGAGA

AGGCATCAAGCCCGTACTTCATTCACCCGCGAACAGTATGAAGAGCTAGAAGCTCTGTTT

AGCGAGACCATGTTCCCAGATAGAAATCGTCAGGAGAAACTAGCTTTGAAACTCAACCTA

TCGGAGTCAACAGTAAAGGTTTGGTTCAGGAACCGGCGATTCAAACAGAAAAAGCAGCAG

CAGCAACAGCAGCAGCAGCAGCAGCAGCAATCAGCAATGCTACCAAACCAGATCCTTCTA

TCCGAGAAGGATGTGCCCACCTTCCTGAGAACATCTATCATTTCTTATGATTTTTATCCT

GTGGTTTCAGATTTCTACAGCTCCCTTCCACCTCAGTCCTTAGGCCCTTCCAATTGGGCA

TGGAACTCTACTTTCACTGAGAGTCCCACAAGTGATTTCCAAATGCAAGATACTCAGTGG

GAAAAGCTGGTAGCCTCAGTTCCTGCTTTGTACTCTGATGCCTATGACATATCCCAAGTC

ATAGAACTGTATGATCTTCCTGATGAGAATGAGATATCCAGCTCTTCTTTCCACTGTCTG

TATCAGTATCTCTCGCCAACAAAGCACCAGATAGGAGGACAGGGTTCCTCTCTCAGCATC

TTTGCTGGTCCAGCTGTAGGCCGATCTCCCATACAAACCTGGCCCAGTATGACAAGCCAA

GGCTTTGAAGCCTACAGTCCAACAGACAGTCTAGAATTCTAGAAAACCTCCAATATGGTA

GCCTTTGGATTACTCTGA

>Marmoset-ARGFXP1

GGAAACTGCATTTTCAGAGAGAGACACAGCAAGCAGGACTGAAAACGGTTTCATTCTGTC

ATCCAAGCATGAGTGTGGGGGCGCAGTCTCGGCTCACTGCAGCCTCAACCTCCCAGGCTC

AAGTGATCCTCCCACCTCAGCCTCCCAAGCAACTGGGACTACAGCAACATGGAGAAGGCA

TCAAGAACGTACTTCATTCACCCGCGAACAGTATGAAGAGCTAGAAGCTCTGTTTAGCGA

GACCAGGTTCCCAGATAGAAATCGTCAGGAGAAACTAGCTTTGCAACTCGACCTACCGGA

GTCAACAGTAAAGGTTTGGTTCAGGAACCGGCGATTCGAACAGAAAAAGCAGCAGCAGCA

GCAATCAACAATGCTACCAAACCAGATCCTTCTATCCAAGAAGGATGTGCCCACCTTCCT

AAGAACAGCTATCATTTCTTATGCTTTTTCTTCTGTGGTTTCGGATTTCTGCAGCTCCCT

TCCACCTCAGCCCTTAGGCCCTTCCAATTGGGCATGGAACTCTACCTTCACTGAGAGTCC

CACAAGTGATTTCCAAATGCAAGATACTCAGTGGAAGAAGCTGGTAGCCTCAGTTCCTGC

TTTGCACTCTGATGCCTATGACATATCCCAAGTCATAGAACTGTATGATCTTCCTGATGA

GAATGAGATATCCAGCTCTTCTTTCTACTGTCTGTATCAGTATCTCTTACCCACAAAGCA

CCAGGTAGGAGGACAGGGTTCCTCTCTCAGTATCTTTGCTGGTCCAGCTGTAGGTCTATC

TCCCATACAAACCTGGCCCAGTATGACAAGCCAAGGCTTTGAAGCCTACAGTCTAACAGA

CAGCCTAGAATTCTAGAAAACCTCCAATATGGTAGCCTTTGGATTTCTCTGA

>Marmoset-ARGFXP2

AGGACTGAAAACGGTTAGTCTAAGGGGATTCAGGACAGCAACATAGAGAAGGCATCAAGA

ATGTACTTCATTCACCCGCAAACAGTATGAAGAGCTAGAAGCTCTGTTTAGCGAGAGCAT

GTTCTGACATTTCCTGATCAATCGTCAGGAGAAACTAGCTTTGAAACTCGACCTACCAGA

GTCAATAGTAAAGGTTTGGCTCAGGAACCGGCGATTCAAACAGAAAAAGCAGCAGCAGCA

GCAGCAGCAGCAGCAATCAGCAACGCTACCAAGCCAGATCCTTCTATCAAAGAAGGATGT

GCCCATCTTCCTGAGAACATTTATCATTTCTTACACTTTTTCTCCTGTGGTTTCAGATTT

CTATAGCTCCCTTCCACCTCAGCCCTTAGGCCCTTCCAATTGGGCATGGAACTCTACCTT

CACTGAGAGTCCCACAAGTGATTTCCAAATGCAAGATACTCAGTGGGAGAAGCTGGTAGC

CTCAGTTCCTGCTTTGTACTCTGATGCCTATGACATATCCCAAGTCATAGAACTGTAGGA

TCTTCCTGATGAGAATGAGATATCCAGCTCTTCTTTCCACTGTCTGTATCAGTATCTCTC

ACCCACAAAGCACCAGGTAGGAGGACAGGGTTCCTCTCCCAGCATCTTTGCTGGTCCAGC

TGTAGGCCTATCTCCCGTACAAACCTGGCCCAGTATGACAAGCCAAGGCTTTGAAGCCTA

CAGTCTAACAGACAGCCTAGAATTCTAGAAAACCTCCAATGTGGTACTTTGGATT

>Marmoset-ARGFXP3

ATGATGAACAGAATGGCCCCAGAGAATCCCCAGACAGGCTCTTCCATCAACGGGAATGAT

TTCAGCATGAAGGTGATATCACCACAGGATCCCGCTAGATATCATCATGAAGGTGATACC

ACCGCAGCATCCAGAAATAGAAAAAATGAGGATGATATTCTCTGGGAGTCCCAGTTTCAT

TCTGTCATCCAAGCATGAGTGCAGTGGCTCGGTCTCGGCTCACTGCAGCCTCAATGTCTC

AGGCTCAAGTGATCCTCCCACCTCAGCCTCCCATGTAACTGGGACTACAGCAACATGGAG

AAGACATCAAGAATGTACTTCATTCACCTGTGAACAGTATGAAGAGCAAGAAGCTCTGTT

TAGTGAGACCATGTTCCCAGACAGAAACTCTCGGGAGAAACTAGCTTTGAAACTTGACCT

ACTGGAGTCAACAATAAAGGTTTGGTTCGGAAACCAGCGATTCAAACAGAAAAAGCAGCA

GCAGCAATCAGCAATGCAATCAAACCAGATCCTTCTATCCAAGAAGGATGTGCCTACCTT

CCTGAGAACATCTATCATTTCTTATGCTTTTTCTCCTGTGGTTTCAGATTTCTACAGCTC

CCTTCCACCTCAGCCCTTAGACCCTTCCAATTGGACTTGGAACTCTACCTTCACTGAGAG

TCCCACAAGTGATTTCCAAATGCAAGATACTCAGTGGGAGAAGCTGGTAGCCTCAGTTCC

TGCTTTGTACTCTGATGCCTATGATATAACCCAAGTCATAGAACTATACAATCTTCCTGA

TGAGAATGAGGTATCCAGGTCTTTTTTCCACTTTCTGTATTAGTATCTCTCACCCACAAA

GCACCAGGTAGGAGGACAGGGTTCCTCTCTTAGCATCTTTGCTGGTCCAGCTGTAGGCCT

ATCTCCTGTACAAACCTGGCCCAGTATGACAAGCCAAGGCTTTAAAGCCTACAGTCTAAC

AGACAGACTATAATTATATAAAACCTCCAATATGGTAGCCTTTGGATTTCTCTGA

>Marmoset-ARGFXP4

AGCAGCAGCAGCAGCAATAAGCAATGCAACCAAACCAAATCCTTCTATTCAAGAAGAGTG

TGCCCACCTTCCTGAGAACATCCAATCATTTCTTATGTTTTTTCTCCTGTGGTTTCAGAT

TTCTATAGCTCTCTTCCTCCTCAGCCCTTAGACCCTACCGATTGCACATGGAACTCTACC

TTCACTGAGAGTCCCACAAGTGATTTCCAAATGCAAGATACTCAGTGGGAGAAGCTGGTG

GCCTCAGTTCCGGCTTTGTACTCTGATGATTATGACATATCCCAAGTCATAGAACTGTAC

AATCTTCCTGATGAGAATGAGATATCTAGCTCTTCTTTCCACTGTCTGTTTCAGTATCTC

TCACCCACAAAGTACCAGGTAGGAGAACTGGGTTTCTCTCTCAGCATTTTTCTGGTTCAG

CTGCAGGCCTATCTCTCACACAAACCTGGCCCAGTATGACAAGCCAAGGCTTTGAAGCCT

ACAGTCCAACAGACAGCCTGGAATTCCAGAAAACCTCCAAATGGTAG

>Tarsier-ARGFXP1

CAGAATGACCCCAGAGAATCTCCAGTCAGACTCTTTTATCAACATAAATGATTTCAGCAT

GAACTTGATGCCATCAAGAAAATACCTCATTCACCCATAAACAGCATGCAGAGTTAAAGG

CTGTTTAACCAGACCATGTTTCCAGACAAAAATCTCCAGAAGGAACTAGCTTTGAAACTC

AACTTAGTGGAGTCAACAGTAAAGGTTTGGCTCAGGAATAGGTGATGCAAATTGAAGAAG

CAGCAGCAACAGTAATCACCAAAGCAATCAAACCAGTTCCGTCCAGTAAAGAGGAATATT

CCTGTTTCACCCAGAACAGCCACTAATCCTTATTTCTTTTTTCCTGTGGTTTCAGATTTC

TACATCTCCCTTCTACCTCAGTCCTTAGACCCTTTCAATTGAGCATGGGACTCTACCTTC

ATTGAGTGTCTCATAAGTGATTTTCAAATGCAAAATTCTCTGTTGGAGAAGCTGGTGGCT

TCAGTTTCTCTTTTATCCTCTGATGCCTATGACATAGACCAAATCATAAAACTACACAGT

CTTCCTGATGACAGTGAAGTATCCAGCTCTTCCTTCTACTGTCTGTATCAGTATCTCTTA

CCCACAGGGCACGAGTTAGTAAGACAAGGTTCCTCTCTTAGCACCCTTGCTGGTCCAGCT

GTAGGTCTATCTCCTGGGCAAACCTGATCCTGTATGACAAACCAAGGCCTTGAAGCCTAC

AATCTAAGAGACAACCTGGAATTCCAGAA

>Mouse Lemur-ARGFX

ATGAAGAGAACAACCCCAGAGAGTCCCCAGCCAGACCCTTCTATCAACATGAATGATTCC

AACATGAACTTGATGTTACCTCAGGATCCAGCTAGGCCCnnnnnnnnnnnnnnnnnnnnn

nnnnnnnnnnnnnnnnnnnnnnnnnnnnnnnnnnnnnnnnnnnnnnnnnnnnnnnnnnnn

nnnnnnnnnnnnnnnnnnnnnnnnnnnnnnnnnnnnnnnnnnnnnnnnnnnnnnnnnnnn

nnnnnnnnnnnnnnnnnnnnnnnnnnnnnnnnnnnnnnnnnnnnnnnnnnnnnnnnnnnn

nnnnnnnnnnnnnnnnnnnnnnnnnnnnnnnnnnnnnnnnnnnnnnnnnnnnnnnnnnnn

nnnnnnnnnnnnnnnnnnnnnnnnGTTTGGTTCAGGAACAGACGATTCAAATGGAAGAAG

CAGAAGCAGCAGCAGCAGCAGCAGCAGCAGCAATCATCAAAGCAACCAAACCAGATTCTT

TCGACAAATATGCCCACTTCATCCAGAATATTCACCCATCCTTACTCTTTATTTCCTGTG

GTTTCAGGTTTCTGTAGCTACGTTGCACCTCAGTCTTTGGATCCTTCTAATTGGGCATGT

GGGTGTGCCTTCACTGAGAGTCCCACAAATGATTTCCAAATGCAGGATCTTCAGTTGGAG

AGGCTGGTGGCCTCTGTTCCTGCTTTGTTCCCTGATTCTTATGACATAGGCCAAATCATG

GAAGTGTACAGTTTTCCTGATGAAGATGAGATATCCTGTTCCTTCCACTGTCTATATCAG

TATCTCTCACCCACAAGTTCCCAGCTAGGAGAATAGGGTTCCTCTATCAGCACCCTTGCT

GTCACAGCTGTAGCTCTATCTCCTAGGCAAGCCTGGTCCAGTAGGACGAGCCAAAGCTTT

GAATATTAAAGTCTAAGAGAAAGCCTTGAATTCCAGAATACTTCCAATATGGTAAACTTT

GGATTACTCTGA

>Mouse Lemur-ARGFXP1

ACCACTTGACCAACAGTCACCCTTTGAGCCAGGGGAGCGCACATTGACAGCATGTTCTCC

CCCCAGGAGGATGAATTCAGGAAAGATACTGTGCAGTCGGCAGCAAAAAATCCTATCGGC

TGTGATATGTGGAGTGTGATTAAGAAGCAGGCCTTCCACCTGAGATTTCAGAACCTCCGA

TGAAGAGAACAACCCCAGAGAGTCCCCAGCCAGACCCTTCTATCAACATGAATGATTCCA

ACATGAACTTGATGTTACCTCAGGATCCAGCTAGGCCCACAACATCAAAGAAACACCAGG

AACGTATCATATTCACTCAAGAACAGTATAAAAAGTTGGAGGCTCTGTTTGGCCAGACCA

TGTTCCCAAATAAAAATACCCAGAAGGAACTAGCTTTGGAACTTAACCTACCAGAGATAA

CAGTGAAGGTTTGGTTCAGGAACAGACGATTCAAATGGAAGAAGCAGCAGCAGCAACAGC

AGCAGCAGCAACAGCAATCATCAAAGCAACCAAACCAGATTCTTCCGACGAATATGCCCA

CTTCATCCAGAATATTCACCCATCCTTACTCTTTATTTCCTGTGGTTTCAGGTTTCTGTA

GCTACGTTGCACCTCGGTCTTTGGATCCTTCTAATTGGGCATGTGGGTGTACCTTCACTG

AGAGTCCCACAAATGATTTCCAAATGCAGGATCTTCAGTTGGAGAGGCTGGTGGCCTCTG

TTCCTGCTTTGTTCCCTGATTCTTATGACATAGGCCAAATCATGGAAGTGTACAGTTTTC

CTGATGAAGATGAGATATCCTGTTCCTTCCACTGTCTATATCAGTATCTCTCACCCACAA

GTTCCCAGCTAGGAGAATAGGGTTCCTCTATCAGCACCCTTGCTGTCACAGCTGTAGCTC

TATCTCCTAGGCAAGCCTGGTCCAGTAGGACGAGCCAAAGCTTTGAATATTAAAGTCTAA

GAGAAAGCCTTGAATTCCAGAATACTTCCAATATGGTAGACTTTGGATTACTCTGATCAA

AGTACTAATGAATACCAGACCATTTAGAAGAGAGACCTTCTTGCCTCTTGTATATGACTC

TTTTCTTTTCTATGATTTTTAACCCAAATGTCTGGGTCTTTGTCTCTTTGATTTCCATGG

AAATGTTGTAATAACTAGAGTTTTCCACACATAGCTCTGCATTACAAAATCAGCCTCATG

ATCCTCTCTGGGGAGAAGTCTTTCTAGTCCTTTAGCCAATGAGACTCCAAACTTCTCTCT

TCCCAGAGCTGTCTTGGACTTTTTAAAAGAGGGACAGTGGCTTTTGGGTTCATCCACTGT

TGATAACAGAAGGTTATAAACTTACATTTTACTTCCAGTCTTTATGTCTGGGGGTTTTAA

AAAGGGACTTTCAGTACAAATTTGAGGAAGAATCATCACCACCATTAGTTTTAACATAAT

TCAACAGTCCAGACATAGTAAGAGGTATAAAAACAGGCATAGGAGAGGGTAAAAGAGATT

TTTGAAGATGAGATGATTATTTAGGAAAACTAACAAAATCAAACTTTTAGAATGACATTT

CAGCACATTTTCAAGCTACATAATCAACACAATTAATAGCATTTCTCAATACCAGTAATA

AAGACAATTAATAAAATAAGTACCTCTTAATAAAAAATTT

>Mouse Lemur-ARGFXP2

ACCACTTGACCAACAGTCACCCTTTGAGCCAGGGGAGCACACGTTGACAGCATGTTCTCC

CCCCAGGAGGATGAATTCAGGAAAGATACTGTGCAGTCTGCAGCAAAAAATCCTATCGGC

TGTAATATGTGGAGTGTGATTAAGAAGCAGGCCTTCCACCTGAGATTTCAGAACCTCCGA

TGAAGAGAACAACCCCAGAGAGTCCCCAGCCAGACCCTTCTATCAACATGAATGATTCCA

ACATGAACTTGATGTTACCTCAGGATCCAGCTAGGTCCACAACATCAAAGAAACACCAGG

AACGTACCATATTCACTCAAGAACAGTATAAAAAGTTGGAGGCTCTGTTTGGCCAGACCA

TGTTCCCAAATAAAAATACCCAGAAGGAACTAGCTTTGGAACTCAACCTACCAGAGATAA

CAGTGAAGGTTTGGTTCAGGAACAGACGATTCAAATGGAAGAAGCAGCAGCAGCAGCAGC

AGCAGCAACAGCAGCAAAGGCAATCATCAAAGCAACCAAACCAGATTCTTTCGAAAAATA

TGCCCACTTCATCCAGAATATTCACCAATCCTTACTCTTTATTTCCTGTGGTTTCAGGTT

TCTGTAGCTACATTGCACCTTAGTCTTTGGATCCTTCTAATTGGGCATGTGGGTGTACCT

TCACTGAGAGTCCCACAAGTGATTTCCAAATGCAGGATCTTCAGTTGGAGAGGCTGGTGG

CCTCTGTTCCTGCTTTGTTCCCTGATTCTTACGACAGGCCAAATCATGGACATGTACAGT

TTTCCTGATGAAGATGAGATATCCTGTTCCTTCCACTGTCTATATCAGTATCTCTCATCC

ACAAGGTCCCAGCTAGGAGAATAGGGTTCCTCTATCAGCACCCTTGCTGTCACAGCTGTA

GCTCTATCTCTTAGGCAAGCCTGGTCCAGTAGGACAAGCCAAAGCTTTGAATATTAAAGT

CTAAGAGAAAACCTTGAATTCCAGAATACTTCCAATATGGTACACTTTGGATTACTCTGA

TCAAAGTACTAATGAATACCAGACCAGTTAGAAGAGAGATCTTCTTGCCTCTTGTATATG

AATCTTTTCTTTTCTATGATTTTTAACCCAAATGTTTGGGTCTTTGTCTCTTTGATTTCC

ATGGAAATGTTGTAATAACTAGAGTTTTCCACATATAGCTCTGCATTACAAAATCAGCCT

CATGATCCTCTCTGGGGAGAAGTCTTCCTAGTCCTTTAGCCAATGAGACTCCAAACTTCT

CTCTTCCCAGAGCTGTCTTAGACTTTTTAAAAGAGGGACAGTGGCTTTTGGGTTCATCCA

CTGTTGATAACAGAAGGTTATAAACTTACATTTTACTTGCAGTCTTTATGTCTGGGGGTT

TTAAAAAGGGACTTTCAGTCCAAATTTGAGGAAAAATCATCACCACCATTAGTTTTAACA

TAATTCAACAGTCCAGACATAGTAAGAGGTATAAAAACAGGCATAGGAGAGGGTAAAAGA

GATTTTTGAAGATGAGATGATTATTTAGGAAAACTAACAAAATCAAACTTTTAGAATGAC

ATTTCAGCACATTTTCAAGCTACATAATCAACACATAAATAGCATTTCTCAACACAGTAA

TAAAGAAAATTAATAAAATAAGTACCTCTTAATAAAAAATTT

>Mouse Lemur-ARGFXP3

CAGGCCTTCCACCTGAGATTTCAGAACCTCCGATGAAGAGAACAACCCCAGAGAGTCCCC

AGCCAGACCTTCTATCAACATGAATGATTCCAACATGAACTTGATGTTACCTCAGGATCC

AGCTAGGCCCACAACATCAAAGAAACACCAGGAACGTACCATATTCACTCAAGAACAGTA

TAAAAAGTTGGAGGCTCTGTTTGGCCAGACCATGTTCCCAAATAAAAATACCCAGAAGGA

ACTAGCTTTGGAACTCAACCTACCAGAGATAACAGTGAAGGTTTGGTTCAGGAACAGACG

ATTCAAATGGAAGCAGCGGCAGCAGCAGCAGCAGCAACAGCAGCAACAGCAGTCATCAAA

GCAACCAAACCAGATTCTTTCGACAAATATGCCCACTTCATCTAGAATATTCACCGATCC

TTACTCTTTATTTCCTGTGGTTTCAGGTTTCTGTAGCTACGTTGCACCTCAGTCTTTGGA

TCCTTCTAATTGGGCATGTGGGTGTACCTTCACTGAGAGTCCCACAAGTGATTTCCAAAT

GCAGGATCTTCAGTTGGAGAGGCTGGTGGCCTCTGTTCCTGCTTTGTTCCCTGATTCTTA

CTACATAGGCCAAATCATGGAAGTGTACAGTTTTCCTGATGAAAATGAGATATCCTGTTA

CTTCCACTGTCTATATCAGTATCTCTCACCCACAAGGTCCCAGCTAGGAGAATAGGGTTC

CTCTATCAGCACCCTTGCTGTCACAGCTGTAGCTCTATCTCCTAGGCAAGCCTGGTCTAG

TAGGACAAGCCAAAGCTTTGAATATTAAAGTCTAAGAGAAAACCTTGAATTCCAGAATAC

TTCCAATATGGTAGACTTTGGATTACTCTGATCAAAGTACTAATGAATACCAGACCAGTT

AGAAGAGAGATCTTCTTGCCTCTTGTATATGACTCTTTTCTTTTCTATGATTTTTAACCC

AAATGTCTGGGTCTTTATCTCTTTGATTTCCATGGAAATGTTGTAATAACTAGAATTTTC

CACATATAGCTCTGCATTACAAAATCAGCCTCATGATCCTCTCTGGGGAGAAGTCTTCCT

AGTCCTTTAGCCAATGAGACTCCAAACTTCTCTCTTCCCAGAGCTGTCTTGGACTTTTTA

AAAGAGGGACAGTGGCTTTTGGGTTCATCCACTGTTGATAACAGAAGGTTATAAACTTAC

ATTTTACTTCCAGTCTTTATGTCTGGGGTTTTAAAAAGGGACTTTCAGTCCAAATTTGAG

GAAAAATCATCACCACCATTAGTTTTAACATAATTCAACAGTCCAGACATAGTAAGAGGT

ATAAAAACAGGCATAGGAGAGGGTAAAAGAGATTTTTGAAGATGAGATGATTATTTAGGA

AAACTAACAAAATCAAACTTTTAGAATGACATTTCAGCACATTTTCAAGCTACATAATCA

ACACAATTAATAGCATTTCTCAATACCAGTAATAAAGAAAATTAATAAAATAAGTACCTC

TTAATAAAAAATTTAAGGTATTTAGGAATTAACAAAGAAAATTAAGACTTCTGTGGAGAA

AACTTTAACACTTAAAAAAGTGACATAGAAAATGGTTTTGATAACAGGCATTTGAGGACA

TAAAATAATAAATGTATTTTTATTTAAAAAAA

>Mouse Lemur-ARGFXP4

GATACTAAAGAGACTGCATTTCCAGAGAGACAGACCAAGCAGGATTGAAAATGGGCACTC

CAAGGGAATTCCTGACAGGCTCTGTTGACACCACTTGACCAACAGTCACCCTTTGAGCCA

GGGGAGCGCACGTTGACAGCATGTTCTCCCCCCAGGAGGATGAATTCAGGAAAGATACTG

TGCAGTCTGCAGCAAAAAATCCTATCGGCTGTGATATGTGGAGTGTGATTAAGAAGCAGG

CCTTCCACCTGAGATTTCAGAACCTCCGATGAAGAGAACAACCCCAGAGAGTCCCCAGCC

AGACCCTTCTATCAACATGAATGATTCCAACATGAACTTGATGTTACCTCAGGATCCAGC

TAGGCCCACAACATCAAAGAAACACCAGGAACGTACCATATTCACTCAAGAACAGTATAA

AAAGTTGGAGGCTCTGTTTGGCCAGACCACGTTCCCAAATAAAAATACCCAGAAGGATCT

AGCTTTGGAACTCAACCTACCAGAGATAACAGTGAAGGTTTGGTTCAGGAACAGACGATT

CAAATGGAAGCAGCAGCAGCAGCAGCAACAGCAGCAACAGCAATCATCAAAGCAACCAAA

CCAGATTCTTTCGACAAATATGCCCACTTCATCCAGAATATTCACCAATCCTTACTCTTT

ATTTCCTGTGGTTTCAGGTTTCTGTAGCTACGTTGCACCTCAGTCTTTGGATCCTTCTAA

TTGGGCATGTAGGTGTACCTTCACTGAGAGTCCCACAAGTGATTTCCAAATGCAGGATCT

TCAGTTGGAGAGGCTGGTGGCCTCTGTTCCTGCTTTGTTCCCTGATTCTTACGACATAGG

TCAAATCATGGAAGTGTACAGTTTTCCTGATGAAGATGAGATATCCTGTTCCTTCCACTG

TCTATATCAGTATCTCTCACCCACAAGGTCCCAGCTAGGAGAATAGGGTTCCTCTATCAG

CACCCTTGCTGTCACAGCTGTAGCTCTATCTCCTAGGCAAGCCTGGTCCAGTAGGACAAG

CCAAAGCTTTGAATATTAAAGTCTAAGAGAAAACCTTGAATTCCAGAATACTTCCAATAT

GGTAGACTTTGGATTACTCTGATCAAAGTACTAATGAATACCAGACCAGTTAGAAGAGAG

ATCTTCTTGCCTCTTGTATATGACTCTTTTCTTTTCTATGATTTTTAACCCAAATGTCTG

GGTCTTTGTCTCTTTGATTTCCATGGAAATGTTGTAATACCTAGAGTTTTCCACATATAG

CTCTGCATTACAAAATCAGCCTCATGATCCTCTCTGGGGAGAAGTCTTCCTAGTCCTTTA

GCCAATGAGACTCCAAACTTCTCTCTTCCCAGAGCTGTCTTGGACTTTTTAAAAGAGGGA

CAGTGGCTTTTGGGTTCATCCACTGTTGATAACAGAAGGTTATAAACTTACATTTTACTT

CCAGTCTTTATGTCTGGGGGTTTTAAAAAGGGACTTTCAGTCCAAATTTGAGGAAAAATC

ATCACCACCATTAGTTTTAACATAATTCAACAGTCCAGACATAGTAAGAGGTATAAAAAC

AGGCATAGGAGAGGGTAAAAGAGATTTTTGAAGATGAGATGATTATTTAGGAAAACTAAC

AAAATCAAACTTTTAGAATGACATTTCAGCACATTTTCAAGCTACATAATCAACACAATA

GCATTTCTCAACATCAGTAATAAAGAAAATTAATAAAATAAGTACCTCTTAATAAAAAAT

TTAAGGTATTTAGGAATTAACAAAGAAAACTAAGACTTCTGTGGAGAAAACTTTAACACT

TAAAAAATGACATAGAAAATGGTTTTGATAACAGGCATTTGAGGACATAAAATAATAAAT

GTATTTTTATTTAAAAAAA

>Mouse Lemur-ARGFXP5

ACCACTTGACCAACAGTCACCCTTTGAGCCAGGGGAGCACACGTTGACAGCATGTTCTCC

CCCCAGGAGGATGAATTCAGGAAAGATACTGTGCAGTCTGCAGCAAAAAATCCTATCGGC

TGTAATATGTGGAGTGTGATTAAGAAGCAGGCCTTCCACCTGAGATTTCAGAACCTCCGA

TGAAGAGAACAACCCCAGAGAGTCCCCAGCCAGACCCTTCTATCAACATGAATGATTCCA

ACATGAACTTGATGTTACCTCAGGATCCAGCTAGGTCCACAACATCAAAGAAACACCAGG

AACGTACCATATTCACTCAAGAACAGTATAAAAAGTTGGAGGCTCTGTTTGGCCAGACCA

TGTTCCCAAATAAAAATACCCAGAAGGAACTAGCTTTGGAACTCAACCTACCAGAGATAA

CAGTGAAGGTTTGGTTCAGGAACAGACGATTCAAATGGAAGAAGCAGCAGCAGCAGCAGC

AGCAGCAACAGCAGCAAAGGCAATCATCAAAGCAACCAAACCAGATTCTTTCGAAAAATA

TGCCCACTTCATCCAGAATATTCACCAATCCTTACTCTTTATTTCCTGTGGTTTCAGGTT

TCTGTAGCTACATTGCACCTTAGTCTTTGGATCCTTCTAATTGGGCATGTGGGTGTACCT

TCACTGAGAGTCCCACAAGTGATTTCCAAATGCAGGATCTTCAGTTGGAGAGGCTGGTGG

CCTCTGTTCCTGCTTTGTTCCCTGATTCTTACGACAGGCCAAATCATGGACATGTACAGT

TTTCCTGATGAAGATGAGATATCCTGTTCCTTCCACTGTCTATATCAGTATCTCTCATCC

ACAAGGTCCCAGCTAGGAGAATAGGGTTCCTCTATCAGCACCCTTGCTGTCACAGCTGTA

GCTCTATCTCTTAGGCAAGCCTGGTCCAGTAGGACAAGCCAAAGCTTTGAATATTAAAGT

CTAAGAGAAAACCTTGAATTCCAGAATACTTCCAATATGGTACACTTTGGATTACTCTGA

TCAAAGTACTAATGAATACCAGACCAGTTAGAAGAGAGATCTTCTTGCCTCTTGTATATG

AATCTTTTCTTTTCTATGATTTTTAACCCAAATGTTTGGGTCTTTGTCTCTTTGATTTCC

ATGGAAATGTTGTAATAACTAGAGTTTTCCACATATAGCTCTGCATTACAAAATCAGCCT

CATGATCCTCTCTGGGGAGAAGTCTTCCTAGTCCTTTAGCCAATGAGACTCCAAACTTCT

CTCTTCCCAGAGCTGTCTTAGACTTTTTAAAAGAGGGACAGTGGCTTTTGGGTTCATCCA

CTGTTGATAACAGAAGGTTATAAACTTACATTTTACTTGCAGTCTTTATGTCTGGGGGTT

TTAAAAAGGGACTTTCAGTCCAAATTTGAGGAAAAATCATCACCACCATTAGTTTTAACA

TAATTCAACAGTCCAGACATAGTAAGAGGTATAAAAACAGGCATAGGAGAGGGTAAAAGA

GATTTTTGAAGATGAGATGATTATTTAGGAAAACTAACAAAATCAAACTTTTAGAATGAC

ATTTCAGCACATTTTCAAGCTACATAATCAACACATAAATAGCATTTCTCAACACAGTAA

TAAAGAAAATTAATAAAATAAGTACCTCTTAATAAAAAATTT

>Mouse Lemur-ARGFXP6

GATAGATACTAAAGAGACTGCATTTCCAGAGAGACAGACCAAGCAGGATTGAAAATGGGC

ACTCCAAGGGAATTCCTGACAGGCTCTGTTGACACCACTTGACCAACAGTCACCCTTTGA

GCCAGGGGAGCGCACGTTGACAGCATGTTCTCCCCCCAGGAAGATGAATTCAGGAAAGAT

ACTGTGCAGTCTGCAGCAAAAAATCCTATCGGCTATGATATGTGGAGTGTGATTAAGAAA

CAGGCCTTCCACCTGAGATTTCAGAACCTCTGATGAAGAGAACAACCCCAGAGAGTCCCC

AGCCAGACCCTTCTATCAACATGAATGATTCCAACATGAACTTGATGTTACCTCAGGATC

CAGCTAGGCCCACAACATCAAAGAAACACCAGGAATGTATCATATTCACTCAAGAACAGC

ATAAAAAGTTGGAGGCTCTGTTTGGCCAGACCATGTTCCCAAATAAAAATACCCAGAAGG

AACTAGCTTTGGAACTCAACCTACCAGAGATAACAGTGAAGGTTTGGTTCAGGAACAGAT

GATTCAAATGGAAGAAGCAGCAGCAGCAGCAGCAACAGCAGCAGCAACAGCAGCAACAGC

AATCATCAAAGCAACCAAACCAGATTCTTTCGACAAATATGCCCACTTCATCCAGAATAT

TCACCCATCCTTACTCTTTATTTCCTGTGGTTTCAGGTTTCTGTAGCTACGTTGCACCTC

AGTCTTTGGATCCTTCTAATTGGGCATGTGGGTATACCTGCACTGAGAGTCCCACAAGTG

ATTTCCAAATGCAGGATCTTCAGTTGGAGAGGCTGGTGGCCTCTGTTCTTGCTTTGTTCC

CTGATTCTTATTACATAGGCCAAATCATGGAAGTGTACAGTTTTCCTGATGAAGATGAGA

TATCCTGTTCCTTCCACTGTCTATATCAGTATCTCTCACCCACAAGGTCCCAGCTAGGAG

AATAGGGTTCCTCTATCAGCACCCTTGCTGTCACAGCTGTAGCTCTATCTCCTAGGCAAG

CCTGGTCCAGTAGGATGAGCCAAAGCTTTGAATATTAAAGTCTAAGAGAAAGCCTTGAAT

TCCAGAATACTTCCAATATGGTAGACTTTGGATTACTCTGATCAAAGTGCTAATGAATAC

CAGACCAGTTAGAAGAGAGATCTTCTTGCCTCTTGTATATGATTCTTTTCTATGATTTTT

AACCCAATGTCTGGGTCTTTGTCTCTTTGATTTCCATGGAAATGCTGTAATTACTAGAGT

TTTCCACCTATAGCTCTGCATTACAAAATCAGCCTCATGATCCTCTCTAGGGAGAAGTCT

TCCTAGCCCTTTAGCCAATGAGACTCCAAACTTCTCTCTTCCCAGAGCTGTCTAGGACTT

TATAAAAGAGGGACAGTGGCTTTTGGGTTCATCCACTGTTGATAACAGAAGGTTATAAAC

TTACATTTTACTTCCAGTCTTTATGTCTGGGGGTTTTAAAAAGGGACTTTCAGTCCAAAT

TTGAGGAAAAATTATCACCACCATTGGTTTTAACATAATTCAACAGTCCAGACATAGTAA

GAGGTATAAAAACAGGCATAGGAGAGGGTAAAAGAGATTTTTGAAGATGAGATGATTATT

TAGGAAAACTAACAAAATCAAACTTTTAGAATGACATTTCAGCACATTTTCAAGCTACAT

AATCAACACAATTAATAGCATTTCTCAATACCAGCAATAAAGAAAATTAATAAAATAAGT

ACCTCTTAATAAAAAATTTAAGGTATTTAGGAATTAACAAAGAAAATTAAGACTTCTGTG

GAGAAAACTTTAACACTTAAAAAAATGACATAGAAAATGGTTTTGATAACAGGCATTTGA

GGACATAAAATAATAAATGTATTTTTATTTAAAAAAA

>Bushbaby-ARGFX

ACAGCAGCGCCAAAGAAGCACCAAGAACGCACCTCATTCACTCCTAAACAGCAGGAAGAG

TTGGAAGCTCTGTTTGGTCAAACCATGTTCCCAGATAAAAATCTCCAGAAGGAGCTAGCT

TTGAAACTCAACCTACCGGAGTCAACAGTGAAGGTTTGGTTCAGGAACAGACGATTCAAA

TTGAAGAAGCAGCAGCAGCAGCAACAACAACAGCAGCAACAGCCATCATCAAAGCCACCA

CACCAGATTCTTCCAACCAAGAATATGTCTACTTCACCCAGAATGGCCACCAATCCTTAC

TCTTCTTTGCCTGCAGTTCCAGATTTTTACAGCTCTCTTTCACATCAGCCCTTTGATCAT

GCCAACCAGGCATGGGGCTCTACCTTCACTGAGAGTCCCACAAGTAATTTCCAAATGGAG

GATATTCAGGAGGAGAACCTGGAGGCCTCCATTCCTGCTTTGTTCTCTGAAGCTTATGAC

ATAGACCAAGTCATAGAAATGTACAGTTTGCCTGATGAGGACGAGACATCTGCCTGTTCC

TTCCAGTGTCTATATCAGTATCTCACACCCACAAGGTCCCAGAGGACAGGGCTCCTATGT

CAGTCCCCTAGGCAAACCTGTGTGACAAACCAAGGTTTTGGAGACTACAGTCTAAGAGAC

AGCCTGGAGTTCCAAGAACACCACCAAAATGAAAGGCATTGAATCTTTCTGA

>Bushbaby-ARGFXP1

TTTCAGATTTCTACAGCTCCCTTCCACCTCTGCCTTTGATCCTTTCACTTAGGCATGGCA

CTCCACCTTCACTGCAAGTCCCACAAGTGATATTTCCAAATGCAAGATCTTCAACTGAAC

AGGCTGGTGGCTTCAGCTTCTGCTTTGTACTCTGATGCTCAGGACATGGGACAAATCATG

GAAATGTATAGTTTTCCTGATGAGAATGAGATATCCATTTCTTCCTTCCACTGTTTATAT

TGGTATCTCTACCCTGTAAGATCCTAGGGGAGGAAGACNNNNNNNNNNNNNNNNNNNNNN

NNNNNNNNNNNNNN

>Tree Shrew-ARGFX

CCGATGAACAGAATGGCCCCAGAGAATCTTCAGGAAGACTCTTCTCTCAACATGAATGAT

TTGCACATAAATTTGATGCCACAGGATCCAGCAAGTCCCAGTGCGTGGAAGAAACATCAA

GAACGTACCATATTCACCCAAAAGCAGCATGAAGAGTTGGAGGCGCTGTTTAGCCAGAAT

ATGTTTCCAGATAAAATTCTCCAAAAAGAACTAGCTTTGAAACTCAACCTGCATGAGTCA

ACAGTAAAGGTTTGGTTCAGAAACAGGAGGTTCAAACTGAAGAAGCAGCAGCAACAACAG

CAGTCACAAAAGCAACCAAACCACATCCTTCCAGCCATGAGGATTATGCCCACCTCACCC

AGAACATGCACAAACCCTCATTTTTTTCCCCCTGTGGTTTCAGATTTCTATAGTTCCCCT

CCATCTCAGCCCTTAGACCCTTCAAATGGGGCATGGGGCTCTGTCTTCACTGAGAGTCCC

ACAAGTGGTTTCCCAATGGAAGATCCTTTGGTGGCCTCAGTTCCTGCTTTGTACTCTGAT

GTCTATGACATTGCCCAAATCATAGACCTGTACAGTTTTCCCGATGAGGATGAGACGTCC

AGCTCTTCCTTCCACTGTCTGTATCAGTATCTCTCACCCACAGGGTCCCAACAAGGAGAA

CAGGGTTCCTCTCTTnnCAGCCCCTTTGCCCATCCAGCTATAAAGCTACCTCCAGGGCCA

ACCTGATCCAGTGTGATGGGCCAAAATTTTGCATCTTGCAACCnAAGAGACAGTCTAGAA

TTCCAGAGCACCTCCAGTATGGTAGACTTGGnATTTCTCTGA

>Tree Shrew-ARGFXP1

AGAAACTGCATATCTGGAGAGACACACATCAAGCAGGATTGACAATGGACTCTCCAAAGG

GATTCATAATGGGAACTCAAGAAACCCTGGTGAACAGAATGGCCCCAGAAAATCTCCAGG

AAGACTTTTCTTCAACATGGATGATGATTTCCACATAAATTTGATTCCAGGATCCAGCAA

GTCCCAGAACATGGAAGAAACATCAAAAATGTATTATATTCACCCACGAACAGTACAAAG

AATTGGAAGCACTGTTTAGCCAGAATATGTTTCGAGATAAAATTCTCCAAAAGGAGCTAT

TTTTTTCTTTTCAAAAGGAGCTATTTTTAAAACTCAACCTATAGGAGTCAACAGTCAAGG

TTTGGTTCAAAAACAGGAGGTTCATGGGGCTGGCAGGTGGTGCAATGGTTAAGGGTGCTG

AACTCCTACATGGCTGACCACGGTTCAAGTCCTGGACCTGGTGGGTTGCTTTCTCACTTT

CCATCTCTCTTGCTCTCTCTCACTCTGTCCCTCTCTGGTCAAATTTAAATAAATAAACAA

ATAAAATAATTACTTAAAAAATAATAAAAAGAAACAGGGAGGTTCAAACTGAGGAAGCAG

CAGCAACAATAGCTGTCACAAAGCCACCAAAAATACATCCTTTCAGTCATGAAGATTATG

CCCACCTCACCAAGAACATGCACAATCTGACCTTTCCCTCGGAGGTTTCAGATGTCTAAG

CTCCCCTCANNNNNNNNNNNNNNNNNNNNNNNNNNNNNNNNNNNNNNNNNNNNNNNNNNN

NNNNNNNNNNNNNNNNNNNNNNNNNNNNNNNNNNNNNNNNNNNNNNNNNTCCAGCTACAG

ATCTCTAGGGCCAACCTGATCCATTGAGACAGGCCAGGCATACAAGAGACAGTCTAGAAT

TCCAGAACACCTCCAGTATGGTAGACTTGGATTTCTCAGGTCAAAAATAATAATAAATAG

ATCATTTAGAAAAGAAAAGAAAAAAAAAA

>Tree Shrew-ARGFXP2

CACAAGAGAGACTGCATTTCCGGGTAGAGACACATCAAGCAGGATTGGCAGTGAACACTC

CAAGGGGGTTCATAATGGAAACCTGATGAACAGAATGGCTTCAGGGAATCTCCAGCCAGA

TTCTTCTCTCAACGTAAATGATTTCCACATGAATTCGATACTGCAGGATCCAACAAGTCC

CAGAACATGGAAGAAACATCAAGAACATACCATATTCACCCACAAACATCATGAAGAGGT

GGAGGCACTGTTTAGCCAGACTGTGTTTCCAGATAAAATTCTCCAAAAGGAACTAGCTTT

GAAACTCAAACTATGGGAGTCAACAGTAAAGTTTGGTTCAGGAGCAGGAGGCTCAAATGG

AGGATGCTGCAACAACAGCAATCACAAAAGCAACCAAACCTTATCCTACCAGCCAAGAAG

ATCATGCCCACCTCACCCAAAACATGAACGAATCCTGATCTCTTTTCCCCTCTGTTCCGA

TTTCTATAGCCCCCCCTCTACTCAGCCTTAGGCCCTTCCAATGGCATAGGACTCTGTCTT

CAATGAGAGTCTCACAGGCGATTTCCTGATGAAGAGCCTTTGGGGCCTCAGTTTCTGCTT

TGTACTCTGATGTCTATGACATCACCATGTCATAGAACTATACAATTTTCCTGATGAGGA

TGAGATTTTCAGTTCTTCCTTCTACTATCTATATCAATATTTCTCACCCACAAGGTCCCA

GCCAGGAGAACAGGGTTCCTCTCTCAGCACCTTTCCCCATCTCCAGGGCCAGCCCGATAC

AGTATGGCAGGCCAAGACTTTGCATCCTGCAGTCCAAGAGGCAGTCTAGAATTCCAGAAC

ACCTCCAGTATGGTGAACAGGATTTCACCACACCACCCTGAG

>Tree Shrew-ARGFXP3

ACCTGCATCTCCTGGAGAAAGAGTGACATCAGCCAGGATTGACAACCGGATCTTCCCAAT

GGGATTCATAATAGGACCTCAAGAAATCCCTGATGAACCAGAATGGGCCCAGACAATCTC

CAAAAGACACTTATCTCAACATGAATGATTTGCACATAAATTTGATCCCACAAGATCCAG

CAAGTCCCAGACCATGTAAGAGACATCAAGAACGTACTATATTCACTCAAAAGCAGCATT

AAGAGTTTAGCCAGAATGTGTTTCCAGATAAAATTCTCCAAAAGGAACTAGCTTTGAAAC

TCAACTACATGACTCAACAGTAAAGGTTTGGTTCAGAAACAGGAGGTTCAAACTGAAGAA

GCAGCAGCAACAACAGCAGTCACAAAAGCAACTAAACCACATCCTTTCAGCTATAAGGAT

TGTGCCCACCTCACCTAGAACATGCACAAACCCTAATTTTTTTTCCTCGTGATTTCAGAT

TTCTATAACTCCCCTGCCCTCCACCTCAGCTCGTAGGCCCTTCAAACAGGGCATGGGGCT

CTGTCTTCGCCAAGAGTCTCAAGATCCTTTGGAGGCCTCAGTTCCAGCTTTGTACTCTGA

TGTCTATGACATTGCCCAAATTATAGACCTGTATACAGTTTTCCTCGTTAAGATGAGACA

TTCAGCTCTTCCTTCCACTGTCTATGTCAGTATCACTCACCCATAATGTCCCAGCTAGGA

GAACAGGGTTCCTTCTCTTCAGCACCTTTGCCTATCCAGCTATAAATCCATCTCCAGGGC

CAACCTGACCCAGTGTGACAGTTCAAGACTTTGTATCTTGCATCCAAGAGAAACTCAACT

CCAGCACACCTTTGGTATGGTAGACTTGTATTTCTCTGATCAAAATACTAATAAATAGAC

>Guinea Pig-ARGFX

ATCTTGAACAGAAGGACTCCAGAAAATCCTGGGCCAGACTTTGTTTTCTTCGATCAGAAT

TTCTCCAGATGGAACTCGTTGGCACCACCGTGTCCTGGTGGACTCAATGCCAGGAGGAAG

CAACAAGGTCGCACCTCATTCACCCGCCAGCAGCAGAAGGAGCTGGAGGCTCTGTTCAGC

CAGACCATGTTCCCAGCGAAGAATGTCCGGGAAGAACTGGCTGGAAGGCTCAACCTGGAG

GAGAAAACCGTAAAGATCTGGTTCCGGAACAGGCGTTTCAAGCTGAGGAAGCAGCAGAAG

CAGGAGGAGCAGTCACTGCAGCAGTCAAAGCAGACGCCTCCACCTGTGAACAGTGTGCCT

TCCTCACTCAGAGCATCCGCCAAAGCACACTACCTCACTTCTCTAGCTTCAGATTTTGAT

GGCTCCCTATCAAGGCGGCACACAAACCCTTGTGACTCATCGGAGGATGAGAGTCCCACA

ACTAATTTCCAAATGCGTGATTTTCAGTTAGACAAGCTGGAGGCCTCTGTTCGTGCTGTG

TGTTCCGATGCCTTTGACATAAGCCAAATCATAGACCTATACAGTTTTCCTGATGAGTGT

GAGGACTCTGACTGTTTTGGCTGTCTGTATCAGTATCTTTCACCTACAACGTCCCAGCCA

GAAGAAAATGATGTCTCTCTCTGCTCCCTTTATGGTCCGGTTATTTGTCCACCTCCTGGG

CAAACCTGCATACCCAGTGTGAGGAGCCAACCCTGTGCTGCCTGGGGTCCCCAGTAGCCT

GGnnCTCCAGAACCCCTCCAGTAnGGCAGACTGTATTTCTAATGGA

>Rabbit-ARGFX

ACTACAGGAATGTGTAAGAAGCATCAACGTACCTCATTCACCCACCAACAGCTTATAGAG

TTGGAGGCTGTATTTAGCCAGACCATGTTTCCAGATAAAAAACTCAAGAGCGAACTAGCT

ATGAAGTTCAACCTACCAGAGTCATCAGTAAAGGTTTGGTTCAGAAACAGGCGATTCAAA

TGGAGGAAGCAGCGACGGCAGTCACTAAAGCAACTCAACCAGGTCCTTCAAGCTGGGAAG

AATATGCCCACCCTCCCCGGAACATCCACTGATCCTTACTCCTTTTTTCCAATGGTTTCA

GAGTCCCATAGCTCTCTTCCACCTCAGCCCTTAAACCCTTGCAATCAGGAATGGGATTCT

CCCTTTGCAGAGAATCCCACAAATGATTTCCAAATGCAGGATCTTCATTTGGAGAGGCTA

GTGGCCTCGGTTCCTGCTTTGTACCCAGGTGCCTGGGACATAGCAGAAATCATATACCTG

TATAGTTTTCCTGATGAGGATGAGCTATCCAGATTTGCCTTCCACAGTCTATTTCACTAT

CTCTCACCCACAAGGCCCCATCTAGAACAATAAGGTTCCACCTCTCTCAGAAGCTGGGCT

GGGGAAGCTGTAGGTTTATCTCCTGGGCCACACTGGACCAATACAACAAGGCAAGGCTTT

GCAGCCTGCAGTCACAGAGGCAGACTAGCAGCTCAGAGCTTTTCCAGTACTGCAGACTTC

GCCTTTCTC

>Pika-ARGFX

ACAGGAATGCTGAAGAAGCGTCAAGAACGTACCATATTCACCCATCAACAGTACAGAGTT

GGAGGCTCTCTTTACACTGACCATGTTTCCAGATAGAAGCCAACTCCAGGAACTAGCCTT

GCAACTCAACCTGCCAGAGCAAAAAAATAAAGGTGTGATTCCGAAACAGGCGATTCAAAT

TTAAGCAGCAGCAGCTATCAATCCAAGTTCTTGCAGCTGAGATGAATCCACCCATGTACC

CTGGAATCTCCATGGATCCTTGCTCTTTGTTTCCCATTGTTTCAGATTTCTATGGCACTC

TTCTACCTCAGCTCTTAAGCCCTTCCTGTGATGAGTGGGATTCCATCTTCACAGATGATC

CCACAGGTGATTTCCAAATGCATGAA

>Squirrel-ARGFX

CTATAGGGAAGAGGCATCATTCACCCTCGGACAGCATGAAGAGTTAGAGGCTCTATTGAG

CCAGACCATGCTCCAAAATAATAACCTCCAGAGGGAGCCAGCTTTGAAACAGCCTACAGG

AGAAACCAGGAAAGTTTTGGTTCAGAAACAGGAGGTTCAAATCGGGGAAGAAGCAGCAGC

AATAACAGTAACCACTAACCACCAAACCAGATCCTTGCAGCTGAGAAGAATGTGCCCACC

TCCCCCAGTACTTTGTCAATCCTTACTCTTTCTCTCCTGTGGCTCCAGGATTCTAGAACC

CCCTTCTACCTCAGCCTATAAGCCCTTCCACTTGGTCAGGGCACTATCTTCATGGAGTCC

CACAAGAGAGTCTCAAATGTAAGACCCTTAGTTGGAGTGGCTGGTGGACTCAGTTCCTGC

CTTGAATTCTGATGCCTGTGACAGCCCAAATCCTAGAACTGTAGTTTTCCTGACAATGGT

GAGATATCCAGCTCTTCCATCCACAGCCTGGATCAGTATCTTTCACCTACGCGGTTCCAG

TTACAAGGACAGGGTTGCTCTTTCAGGACATTTGCTGGCCCAGCTGTTGTTCTATCTCCC

AGGCAAACCTGGTCGAGTAGGACAAGCCAAGTCTTCGCAGCCTGCAGTCTATGAGACAGC

CCAGGGTTCCAGAACCCCTCCAGTGGGCTGGAACTTTGCATTTCTCTGA

>Cat-ARGFXP1

ATCTGGTTCAGGAACCAACAATTCAAATTGAGGAAGCAGCAGCAGCAACTACTAAAGCAA

CCAAACCAGACTCTTCCATCCAAGAATGTGCACATCTCAACCACAAAGTCAATCAGTCTT

CATTATTTTTTTTCCTGCAGCTTCAGATTTCTGTAGCTTCTTTCCACATCAGCCCTTAGG

CCCTTTCAACTGGGCATGGGACTCTCATCACTGAGATATCCAAATGTAAAATCCTTAGTT

GGAGAAGCTAGTGGCCTCAGTGTCTTGCTTTGTACTCTGATACCTATGACATAGCCTAAA

TCATGAAAGTATACAGTTTTCCTGATGAGGGTGAGACATCCTGCCATTCTTTTCATTGTC

TATGTAAGTATCCCTCACCAACAAGATCCCAGCTAGAAGAAAAGAGTTCCTCTCTCCACA

TCTGTGCTGGTCCAGCTGTAGGTCTATCTCCTGGACAAATCAGGTCGAGTATGACAAGCT

GGGGCTTTGCAGTCTAAGAGATTGCCTGGAATTC

>Dolphin-ARGFX

ACCTCATTCACACACACACACACACACACACACACACACACACACACACAGCATGAAGAG

TTGGAGGCTCTGTTTAGCTGCAATATGTTTCCAGATAAAAACCTCCAGAGAGAACTTGCT

TTAAAACTCAACCTACCAGCGTCACCAATAAAGATTTGGTTCAGGAATCGGCAGTTCAAA

AAGAGGAAGCAGCAGTGGCAACAAGAGCAGCAATCACTGAAGCCACCGAACCAGGTCCTT

CCAGCCAAGAATGTGCCCACAGCATCAACCAGTCCTCATTCTTTTCTCCCTGCAGTTTCA

GATTCTTATAGCTCCCACTCACCTCAGCCCTTAGACCCTTTCCATTGGGCAAGGGACTCT

ATAATCACTGAGATTGCTACAAGTGATGTCCAAATGCAAGATCCTCAGTTGGAGAGGCTA

GTGGCCTCAGTTCCTGCTTTGTACTCTGATGCCTATGACATAGCACAAATCATGGAACTG

TACAGTTTTCCTGATGAGGATGAGATAGCCAGCTCTTCTTTCCATTCTCTGTATCATTAT

CTCTCACCGACAAAGCCCAGTTAGAATAGAGTTCCTTTCTTAGCCTCTTTGCTGATCCAG

CTGTAGGCTTCTCTCCTGGGCAAACCTGCTTCAGTATGACAAGCTGGAGCTTTGCAGCCT

ACAGTCTAAGAAACAGCCTGGAATTCCAGAACCCCTTCAGTATGGCACACTTTGGA

>Dolphin-ARGFXP1

ACCTCATTCACACACACATAGCATGAAGAGTTGAAGGTTCTGTTTAGCTGTAACATGTTT

CCAGATAAAAACCTCCAGAGAGAACTTGCTTTAAAACTCAGCCTACCAGAATGAACAGTA

AAGATTTGGTTCAGGAATTGGCGGTTCAAAAAGAGGAAGCAGCAACAGCGACAAGAGCAG

CAGTCGGTCCTTCCAGCCAAGAATGTGCCCACAGCATCAACCAGTCCTCATTCTTTTCTC

CCTGCAGTTTCAGATTCTTATAGCTCCCACTTACCTCAGCCCTTAGACCCTTTCCATTGG

GCAGGGGACTATCATCACTGAGATTGCTACAAGTGATGTCCAAATGCAAGATCCTCAATT

GGAGAGGCTAGTGGCCTCAGTTCCTGCTTTGTACTCTGATGCCTATGACATAGCACAAAT

CATGGAACTGTACAGTTTTCCTGATGAGGATGAGATAGCCAGCTCTTCTTTCCACTCTCT

GTATTAGTATCTCTCACCGACAAGGCCCAGTTAGAATAGAGTTCCTCTCTTAGCATCTTT

GCTGATCCAGCTGTAGGTTTATCTCCTGGGCAAACCTGCTTCAGTATGACAAACTGGAGC

TTTGCAGCCTACAGTCTGAGAGACAGCCTGGAATTCCAGAACCCCTCCAGTATGGTACAC

TTTGGA

>Cow-ARGFX

CTGATGAACAAAAGAGTCCAAGAGAATCCCCTGCCAGACCCTATGATTCTAGCACGAACT

TCCAGGATGATnnnnnnnnnnTCAAAGATACCAGCTGGTGCCACAAAATGGAAGAAGAGT

CATGAACGCACCTCATTCACCCACACACAGTACAAGGAGTTGGAGGCTCTGTTTAGCTGC

AACATGTTTCCAGATAAAAACCTCCAGAGAGAACTTGCTTTAAAACTCAATCTACCAGAG

TCAACAGTAAAGATTTGGTTCAGGAACCGGCGGTTCAAAATGAAGAAGCAGCAGCGGGAG

CAAGAGCAGCAATCACTAAAGCCACCAAGCCAGGTCCTTCCAGCCAAGGATGTGCCCACA

GTATCAACCAGCCCTCATTCTTTTCTCCTTGCAATTTCAGATTCCTATAACTCCCTCTCA

CCTCAGCCCTTAGACACTTTCCCCTGGGCAGGGGACTCTATGATCATGGAGATTCCTACA

AGTGATGTCCAAACGCAAGATCCTCAACTGGAGAGGCTAGTGGCCTCAGTTCCTGCTTTG

TACTCTGATGCATTTGACATCACCCAAATCATGGAACTGTACAGTGTTCCTGATGAGGAT

GACATCACCAACTCTTCCTTCTATTCTCTATATCAGTATCTCTCACCGACAAGGCnnCCA

GTTAGAATAGAGTnnTCTTCTCTTATAGTCTTTGCTGATCCAGCTGTAGGCTTATTTCCT

GGGCAGACCTCCTTCAGTGTGACAAGCTGGAGCTTTGCAGTCTACAGTCCACAGGACAGC

CTGGAATTCCAGAACCCCTCCATTACAGTGCACTTTGGATTTCTCTGA

>Cow-ARGFXP1

AACAAAATAGTCCAAAAGAATCCCCAGCTAGACCCTTTTGTCTACATGGATGATTCCAGA

ATGAACTTCCAGGATGATTCACAGATACCAGCTGGTCCTACAAAATGGAAGAAGTCTCAT

GGATGCACCTCATTCACCCTCACCCACACACAGTATGAGGAGTTGGAGGCTCTGTTTAGA

TGCAACATGTTTCCAGGTAAAATCACCCAGTGAGAACTTGCTTTAAAACTCAACCTACTG

GAGTCAACAGTAAAGATTTGCTTCGAGAATCGGCGGATCAAAATGATGAAGCAGCAGCAA

TAACAAAATCAGCAATCACAAAAGCCACAAAAGTCTTGGTCCTACCAGCCAAGACTGCAC

CCACAGCATCAGCCAGTCCTCATTCTTTTCTCCCTGGAGTTTCAGATTCCTGTAGCTCCC

TCTCACGTCAGCCTTTAGACCCTTTCCATTGAGTAGAGGACTCTGTCATTCCTGAGATGC

CTATAAGTGCTGTCCAAATGCAAGATCCTTAACTGGAGAGGCTAGTGGCCTCCATTCCTG

CT

>Horse-ARGFX

CTGATGAATGGAATGGCCCCAGATAATCCCCAGCCAGACCTTTTTATCAACTTGGATGAT

TCCAGCATGAACTTGATACCACAGGATCCAGCTGATCCCACAACATAGAAGTAGCACCAA

GAATGCACCTCATTCACTCACAGACAGCATGAAGAGTTGGAGGCTCTGTTTAGCCnnACC

ATGTTTCCAGATAAAAATCTCCAGAAGGAATTGGATTTGAAACTCAACCTACCGGGGTCT

ACAGTAAAGACTTGGTTCAGGAACGGGCAATTCAAACTGAGGAAGCAGCAGCAGCAACAA

CAGCAATCACTAAAGCAACAAAACCAGATCCTTCCAGCCAAGACGGTGCCCAACTCACCC

ACAGCATCAAGCAGTCCTTGTTCTTTTGTTTnnCCTGCAGTTTCCGATTTCTGTAGCTCC

TTCCnnACCTCAGCCCTTAGGCCCTTCCCTTGGGCCTGGGACTCGATCATCACTGAGAGT

CCCACAAGAGATGTCCAAATGCAGGATCTTCAGTTGGAGAGACTAGTGGCCTCAGTTCCT

GCCTTGTACTCTGATGCCTATGACATAGCCCAAATCATGAAACTGTATAGTTTTCCTGAT

GAAGATGAGATATCCAGCTCTTCTTTTCAGTATCTGTATTGGTATCTCTCGCCCACAAGG

CCCCAGCTGGAAGAACAGAGTTCCTCTCTTAGCATCTTTGCTGGTCTAGGAGTAGGTCTA

TCCCCTGGGCAAACCTGGTCCAGTATGACAAGCCAGCATTTTGCAATCAACAGTCTAAGA

GGGGTTCTAGAATTCCAGAACCCTTCCAGTATGGTGGACTTTGGAGTTCTTTGA

>Megabat-ARGFX

ATGAACAGAGGGGCTTCTGGGACTCCCCAGACTCACTCTTTTTTACACATGAGTAATTAC

AGCATTGACATGATACAAGAAAAAGTTGnnATCACAGCGACAATGTGGAAGAAGCACCCA

AAATGCACCTTGTTCATGCATACACAGCATGAAGAGTTGGAGGCTCTGTTTAGCCACACC

ATGTTTCCAGATAAAAATCTTCAGAAGGAATTTGCTTTGAAACTCAACCTACCAGAGTCA

ACAATAAAATCTACTTGGTTCAGGAACCAGCAAGTCAAATTGAGGAAGCAGCAGAAGCAG

CGGCAGCAGCAGTGGCAGCAGCAGCGGCAACAGCTATCACTAAAGCAACCAAACCAGATT

CTTCCAGCCCAGAATGTGCCCTCCTCACCCACAACATCAACCAGTCTATATTCTTTTTTT

CCTATAGTTTCAGATTTCTATAGCTCCCTTCCACCTCAGCCCTTAGGCCCTCCCAACTGG

GCGTGGGACTCTATCACTACTGAGAGTCCCACAAGTGATGTCCAAATGCAAGGTCCTCAG

CTGGAGAGGCTAGTGGCCTCAGTTCCTACTTTGTATTCTGATGCCTATGACATAACCCAA

ATCATGGAACGGTACAGTTTACCTGATGAGGATGAGGTATCCCACTCTTTCTACAGTCTA

TATCAGTATCTCTCACCCACAGGCCACnGGCTAAAAGAACAAGGTTCTTTTAGCATCTTT

GCTGGTCCAACTGTAGGTCGATCTCCTGGTAAATCTCATCCAnGTATGACAAGCTGAGGC

CTTGCAGCCTATAGTCTAAGAGACAGCCTGGAATTCCAGAACCTCTCCAATATGATGGGC

TTTGGATTCCCCTGA

>Megabat-ARGFXP1

AGCAAGTGTTTCCTGCCCATCCAATCTAGTAATGAAATAATTACAATTACAATCAATATT

TACTGATCACTCTGTGCCATCTATCAAATCCATAACAATGATAACGGTGGTCAAAACAAC

AAAAATATATTTATTGAGTCTTTATTACATGCTCAGCACTGTTCAAAGTTCATCACGTAC

TTGTATCATTTAATCCTTACAATATAATGTACCCATGATCCCAAAACAAGCAGGAAAAAT

TGTACCCCGTTGATATCAACTTACAGGCTTTGTCATACAAATATATGATATTAAAGTGAA

TAATTTAATAGTTTTTTTAAATGTCTGAAAATGGGGGGAGGGTAGAAATGTCCTTTATCA

ATTACTCGTGTCCCTGGAACTTTAATGTTTTCTTCATTAAAATAGCACAACTATTGACAC

TAAATAAACTGCATTTTCAGAGAGACACATCCAGCAGGCTTGAAATGGGCACTCCAAGGG

GATCAAGACAGACCTACCAATGCAGCTGTCTGGTGCCTACACAAACAGAGGAGCTCCTGG

GACTCCCTACATGAGTGCATGAGTGATTTTTATCCACATGAATGATTATAGCATTGACGT

GATACAACAAGACAAATTTGATCACAGCTTTTCATCCCAGATGTCAGCGTCCCCGATGAA

CAGAATGGCTCCAGACAATCCCCAGCCAGACCCTTTTATCCACATGGATGATTCCAGCAT

GAACTTCATACCACAGAATCAAGCCGGTCCCACAATATGGAAGAGGTACCAAAAACTCAC

CTCATTTATGCACACACAGTATGAAGAGCTGGAGGCTCTGTTTAGCCACACCACATTTCC

AGATAAAAATCTCCAGAAGGAACTGACACTGAAACTCAACCTACTGGATTTGAGTCAACA

ATAAAGACTTAGTTCAGGAACCAGCAAGTCAAACTGAGGAAGCAGCAGCAACAACAGCTA

ACACTAAAGCAAACAAACTAGATTCTTCCAGCCAAGAATGTGCCCGCCTCACCCACAACA

TCAAGTAGTCCTTATCCTTTTTTTCCTGTAGTTTCAGATTTCTATAGCTCCCTTCCACCT

CATCTCTTAGGCCCTTTCAATTGGGCATGGGAGTCTACCCTCACTGAGAGTCCTATAAGT

GATGTCTAAATGCAAGGTCCTCATTTGGAGAGGTTAGTGGCCTCGGTTCCTGCTTTGTAT

TCTGATGCCTATGACACAGCCCAAATCATGGAACTGGATAGTTATCCTGATATAGATATC

CTGTCTATATCAGTGTCTCTCATCCACAAGCCCCAGCTAGAAGAACATGGTTCTACTTTC

AGCATCTTTGCTGGTCCAGCTGTATGTCTATCTCTGGCAAACCTCGTCCAGTATGACAAG

CTGAGGTCTTGCAGCCTAAAGTCCAAGAGACATCCTGGAATTCCAGAACTCCTCCAATAT

GATGGACTTTATTTCTCTGATCAAAGTAGTAATAAATACCAGATTGTACAGAAAAAAAAT

CA

>Megabat-ARGFXP2

GAAGCACCCAAAATGCACCTTGTTCATGCATACACAGCATGAAGAGTTGGAGGCTCTGTT

TAGCCACACCATGTTTCCAGATAAAAATCTTCAGAAGGAATTTGCTTTGAAACTCAACCT

ACCAGAGTCAACAATAAAGATCTGTCTGATTCCCTGAGGTGTGCTTGATACCTCTATCCA

AACTATGTTATACTATTCAGCCTGGAAATTCTCTCCATAACACAATATTTTATTGAGTAC

CTACTGTGTGCTAGTACTGAACTAGGCACATTGCATATGTTGGCCTCACTATTATCCAGT

TTAAAAGAAGTGAAAAACAGGTTCAAAGTGAAGTGACTGGGCCACAGAGTTAGTAAGTAG

TATAACTGGAATTAGAGATCCAAAGGTCTTGTACTATAGAGCTCAAATAGGGGAGGGACT

CTCATGCTGACATTCCTCACTCTAAACAGATATACAGTCCATTAGACTGTCACTCTGGTT

CTTCTCAAGCAAATATGTGTAATCTGCTTATTCACAACACCACCAACAAACAAATAACTC

CCCCAACTTCTGAGTCAGCTAAGGCTCAACTTTTATTTTTATTTATTTTTATTTTTTTAG

AGTATTCCTCACTATTGAGGCCTCCTAACTAACGCTTAAAAATTCCTTTCGTTCAAATTC

CTCACCTTCTTCTAGGTCCCTGATCTAGTTTCTCTTCTCCAGAGTTCAGAACCCTTTCTC

TGAACTCTCCAAACAACCTCTTAACATACCCCCTTTTCTTCTTTCTTCTCTGGGGTCTCA

GTCCTGACCCTTCATTGTCCCTTCTATTGACTCTACTCATGGCCACCTCTGCTATCTCAG

AACTCAGTGACATGGTGACTTAAAGAGGATATGGGGCTTGTAATCTGGCTTTTTTTACCC

AGGAGACGCCTCAGACTTCTCCACTACAGACCAATCTCCCCTCTTTTCCTAGACTTGGTT

CAGGAACCAGCAAGTCAAATTGAGGAAGCAGCAGAAGCAGCGGCAGCAGCAGTGGCAGCA

GCAGCGGCAACAGCTATCACTAAAGCAACCAAACCAGATTCTTCCAGCCCAGAATGTGCC

CTCCTCACCCACAACATCAACCAGTCTATATTCTTTTTTTCCTATAGTTTCAGATTTCTA

TAGCTCCCTTCCACCTCAGCCCTTAGGCCCTCCCAACTGGGCGTGGGACTCTATCACTAC

TGAGAGTCCCACAAGTGATGTCCAAATGCAAGGTCCTCAGCTGGAGAGGCTAGTGGCCTC

AGTTCCTACTTTGTATTCTGATGCCTATGACATAACCCAAATCATGGAACGGTACAGTTT

ACCTGATGAGGATGAGGTATCCCACTCTTTCTACAGTCTATATCAGTATCTCTCACCCAC

AGGCCACGGCTAAAAGAACAAGGTTCTTTTAGCATCTTTGCTGGTCCAACTGTAGGTCGA

TCTCCTGGTAAATCTCATCCAGTATGACAAGCTGAGGCCTTGCAGCCTATAGTCTAAGAG

ACAGCCTGGAATTCCAGAACCTCTCCAATATGATGGGCTTTGGATTCCCCTGATCAAAGT

ACTAATGAATATCAGCTAGTACATAAAAAAGACT

>Alpaca-ARGFX

CAGATTTGGTTCAGGAATCGGCGGTTCAAAATGAGGAAGCAGCAGCAGCAGCAACAGAAA

TCACTGAAGCCACCAAACCAGATCCTTCCAGCCAAGAACGTGTCAACAGCATCAACAAAT

CCTCATTTTTTCCCCCCAACAGTTTCAGATTTCTGTAGCTCCTTCTCACCTCAGCCCTTA

GGCCCTTTCAGTTGGGTGGGGGACTCTATCCTCACTGAGAGTCCTACAAGTGATGTCCAA

ATCCAAGATCCTCAGTTGGAGAGGCTGGTGGCCTCAGTTCCTGCTTTGTACTCTGATGCC

TATGACATAGCCCAAATCATGGAAATGTACAGTTTTCCTGATGAAGATGAGATAGCCAGC

TATTCTTTCCACTCTCTCTATCAGTATCTCTCACCAACAGGCCCAGTTAGAATAGAGTTC

CTCCCTTAGCATCTTTGCTGGTCCAGCTGTAGGTTACCTCCTGGGCAAACCCAGTTTAGT

ANNNNNNNNN

>Alpaca-ARGFXP1

AATAAAATGGCCCAAGAGAACTCCCAGCCAGATCCTTTTATCTATATGGATGATTCCAGC

ATGACTCTCAGGATGATTCACATACCAAGCTGGTCCCAANNNNNNNNNNNNNNNNNNNNN

NNNNNNNNNNNNNNNNNNNNNNNNNNNNNNNNNNNNNNNNNNNNNNNNNNNNNNNNNNNN

NNNNNNNNNNNNNNNNNNNNNNNNNNNNNNNNNNNNNNNNNNNNNNNNNNNNNNNNNNNN

NNNNNNNNNNNNNNNNNNNNNNNNNNNNNNNNNNNNNNNNNNNNNNNNNNNNNNNNNNNN

NNNNNNNNNNNNNNNNNNNNNNNNNNNNNNNNNNNNNNNNNNNNNNNNNNNNNNNNNNNN

NNNNNNNNNNNNNNNNNNNNNNNNNNNNNNNNNNNNNNNNNNNNNNNNAGAACTCCCAGC

CAGATCCTTTTATCTATATGGATGATTCCCTCATGAACTCTCAGGATGATTCACATACCA

GCTGGTCCCAAGAAATGGAAGAAGCACCAAGAATGCACCTCATTCACTGAAACACAGCAC

AAAGAGTTGGAGGCTCTGTTTAGCTGCAACATGTTTCCAGATAAAAATCTCCAGAGAAAA

CTTGCTTTAAAACTCAACCTACCAGACTCAACCCAGTAAAGACTGGGTTCAGGAATCAGT

GGTTCAAAATGAGGAAACAGCAGCAGCAGCAACAAAAATCGCTGAAGCCACCAAACCAGA

TCCTTCCAGCCAAGAATGTGCCAACAGCATCAACAAGTCCTCATTTTTCCCCCTATGGTT

TCAGATTCCTGTAGCTCCCTCTCACCTCAGCCCTTAGGCCTTTTCAATTGGGCAGGGGAC

TCTATCCTCACTGAGAGTCCTACCAGTGATGTCCAAATCCAAGATCCTCAGTTGGAGAGA

CCAGTGGCCTCCGTTCCTGCTTTATACTCTGATGCCTATGATACAGCCCAAGTGGAAATG

TACAGCTTTCCTGATGAAGATGAGATAGCCAGCTCTTCTTTCCACTCTCTATATCAGTAT

CTCTCACCAACAAGGCCCAGTTAGAATAGAGTTCCTCTCTTAGAATCTTTGCTGGTCCAG

CTGTACATTTATCTCCTGGGCAAACCTGGTTCAGTATGACAAGCTGGAGCTTTGCAGCCA

GTCTACAGCCTGAGAGAACCTAGAAT

>Alpaca-ARGFXP2

GGAACTTATGACAGATGTCAGCACCCTTGATGAACAAAATGGCCCAAGAGAACCTCCAGG

CAGATCCTTTTATCTATGTGGATGATTCCAGCATGAATTCCCAGGATGATTCACAGAAAC

CTGCTGTTCAAAATGAGGAAGCAGCAGCAGCAGCAACAATCACTGAAGCCACCAAACCAG

ATCCTTTTAGCCAAGAAAGTGCCAACAGCATCGAGTCCTCATTTTTTCCCCTCCCCAGTT

TCAGATTTCTGTAGTTCCCTTTTACCTCAGCCTTTAGGCCCTTTCAATTGGGCAGGGGAC

TCTGTCCTCACTGAGAGTCCTACAAGTGATGTCCAAATCCAAGATCCCCAATTGGAGAAG

CTAGTGGCCTCAGTTCCTGGTTTGTACTCTGATGCCTATGACATAACCCAAATCATGGAA

GTGTACAGTTTTCCTGATGAAAATGAGATAGCCAGCTCTTCCTTCCACTCTCTGTATCAG

TCTCTCTCACCAACAAGGCCAAGTTAGAATAGAGTTCCTCTCTTAGCATTTCTTCTGGTC

CAGCTGTAGGTTTATCTCCTTGGCAAACCTGGTTCAGTATGACAAGCTGCAGCCTTTGCA

GCCTACAGCCTAAGAGAGCCTAGAATTCCAGAACCCCTCCAGTAGTATGGACTTTGGATT

TCTCTGATCAAAGCACTAATCAACATCAGACAGTATAGAAAAAGGATCTTGCACCTTGTA

>Alpaca-ARGFXP3

NNNNNNNNNNNNNNNNNNNNNNNNNNNNNNNNNNNNNNNNNNNNNNNNNNNNNNGCATCA

AGTCCTCATTTATTCCCCCCAACAGTTTCAGATTTCTGTAGCTCCTTCTCACCTCAGCCC

TTAGGCCCTTTCAGTTGGGTGGGGGACTCTATCCTCACTGAGAGTCCTACAAGTGATGTC

CAAATCCAAGATCCTCAGTTGGAGAGGCTGGTGGCCTCAGTTCCTGCTTTGTACTCTGAT

GCCTATGACATAGCCCAAATCATGGAAATGTACAGTTTTCCTGATGAAGATGAGATAGCC

AGCTATTCTTTCCACTCTCTCTGTCAGTATCTCTCACCAACAAGGCCCAGTTAGAATAGA

GTTCCTCCCGTAGCATCTTTGCTGGTCCAGCTGTAGGTTTACCTCCTGGGCAAACCCAGT

TTAGTACAACAAACTGGAGCTTTGCAGCCTTCATCTTAAGAGAGAGCCCAGAATTCCAGA

ACCCCTCGAGTAGTGTGGTGGACTCTGGATTTCTCTTATCAAAGTACTAATAAATGGCAG

>Alpaca-ARGFXP4

TCCAGCATGAACTCCTGGGATGATTCACAGATACCAGCTGTTCCTAAGAAGTGGAAGAAG

CATCAAGAATGCACCTCATTCACCCAAACACAATGCAACGAGTTGGAGGCCCTGTTTAAC

TGCAACATGTTTCCAGAGAAAAACATCTCCAGAGAAAACCTGCTTTAAAACTTAACCTAC

TGAGTGCAACAGTAAAGACTGGGTTCAGGGACTGGCAGTTCAAAACAAGAAGCAGCAGCA

ACAGAAATCACTGAAGCCATCAGACCAGATCCTTCCAACCAAGAACGTGCCAACAGCATA

AGCAAACCCTCGTCGCCCCCACCCCCCCTGCCGGTTTCGGATTTCTGTAGCTCCCTCTCA

CTTCAGCCCTTAGGCCCTTTCAGTTGGGCAGGGGACTCTATCCTCACTAGGGTTCCTACA

AGTGATGTCCAAATTCAAGACCCTCAGTTGGAAAGGCTAGTGGCCTCCGTCTCTGCTTTG

TACTCTGACGCCTATGACATAGCCCAAATCACAGAAATGTAGTTTTCCTGATGAGGCAGA

GATAGCCAGCTCTTCCTTTCACTCTCAGCATCAGTGTCACTCACCAGAAAGGCCCTGTTG

GAATAGAATTCCTCCCTTAGCATCTTTGCTGGTCCAGCTGTAGGTTTATCTCCTGGGGTG

>Alpaca-ARGFXP5

CTATAATTAAGAAGCTGATTTTGACACTAAAGGAATTGCATTTCCAGAGAAACACATCAA

GAAGTCTTGAAAATAGGCAATCCAAGGGAATTTATGACAGGCTTTCTTTGCCAGATGTCA

GCACCCCTGATGAACAAAATGGCCCAAGAGAACCCCCAGCCAGATCCTTTTATCTGTGTG

GATGATTCCAGCATGAACTCCCAGGATGATTCACAGTTACTGGCTGGTCCCAAGAAATGG

AAGAAGCATCAAGAACGCACCTCATTTACCCAAACACAGCACAAAGAGTTGGAGGCTCTG

TTTAGCTGCAACATGTTTCCAGATAAAAATCTCCAGAGAAAACTTGCTTTAAAACTCAAC

CTACCAGACTCAACAGTAAAGATTTGGTTCAGGAATAGGCAGTTCAAAATGAGGAGCAGC

>Shrew-ARGFX

CTGATGGAGAGGATGGGCCCAGAGCCGTTAGACTCTTTAACCAACATGAATGAGTCTAGC

ACACCCTTGACGGCACAAACCACAACTGAGCCTGAAGTAAGAAAGATGCGCCACAAACGC

ACTTCATTCACCAAAGAACAGCATGAAGAGTTGGAGTCTCTGTTTAGCCATACCATGTTT

CTGGATAAAAATCTTCAGAAGAAAATGGCTTTGAAACTCAAGCTACCAGAGTCAACAGTA

AAGAATTGGTTCAAGAACCGGCGACTCAAGTGGAGAAAACAGAATCAGCAAGAGCAACCA

CTAAAGATGTCAAAAAAGAAACTTTTAAACAACAAACTGCCCAACTTACCCCAACAACCT

ACGAATGCTCATACTTCTTACCCTGCTGTTTCAGATTTCTGCAGCTCCTCTCACACTCAT

CCCTTAGGCCCTTCCAACTGCACACAGGACTCCATCATGACTCTGAGTCTACCAAGTGAC

ATCCAGATGCCCGATACTCAGTTGGAAAGTCTAGAGGCCACAGTTCCTGCTTTGTTTCCT

GATTCCTATGACATAACCCAAATCATGGAACTGTATAGTTTTCCTGATGAGGAGCTAACC

AGTTCTTCATTTGAATGTCTTTATCAGTATCTCTCACCCCAAAGCCCTAGATAGAAGAAC

AGGCTTTCTCTTTTAGCTCCCTTTTTTGTCCATCTGCTGGACATACCTAGAGTTTTTTCA

TGGAGAAAAATACAAGAATTCACCGCCAATACAGTGTTTCTCAAAGTGGGTGATACTAAC

CCCCTGTCTCCAAGTGCTGGAACATCTAGGATGAGTGGTAGCATTAGGTGCAATTGA

>Elephant-ARGFX

GAACGTACTGTATTCACCCACAAGCAGCATGAAGAGTTGGAGGCTCTGTTTAACCAGACC

ATGTTCCCAGATAAAAATCTCCGCATGGAACTGGCTTTGAAGTTCAATGTACAGGAGTCA

AAAGTGAAGGTTTGGTTCAGGAACCAGCGATTTTAAAATGAAGAAGCAGCAGCAACATCA

GCACTCACTGAAGAAACCAAGGCAGATTATTCCAGCCAAGAAGAATGTGTCCACCTTATT

GAGGGCATCCAGCAGTCCTTATTCTTTTTATCCTGTGGTTTCAGATGGCTATAGCTCCCT

TCCACCCCGGCCCTTAGGCACTTTCAACTGGACACAAGACTCTGTCTTTACCTGGAATTC

CACAAGTGATGTCCAAATGCAAGATCCACAGTTGGAGAGGCTGGTAGCTTCAGTTCCTGC

TTTATACTCTGATGTTTATGACATAACGCAAATCATGGAACTGTATAGTTTTCCTGACAA

GGATGTGGCATCCAGCTCTTCCTTCAGCTGTCTGTATCAGTATCTCTCACCCACAAGGCC

CCAGCTGGAAAGATGGGGTCCTGCTCTCAGCATGTTTGCTGATCCAGCAGTAGGTCTATC

TCCTGGGGAAACCTGGTCCAATATGACAAATGGAGGCTTTATAGCCGACAGTCTGAGAGA

CAGTCTGGAATTCCAGAATCCCTCTAGTATGGTGCATTCTAGATTT

>Tenrec-ARGFX

AGACAGCGTTTTATGTTCACCCCAAAACAGCAAGAAGTGTTGGAGGCTATGTTTGAACAG

AACCGTGTTCCAAATAAAGAGACCCGGATGCAAGTGGCTTTGCAACTCAATGTGGAGGTC

GAAAAAGTGAAGGTTTGGTTCAGGAACAAGCGCTTTAAAATGAAGCAGATGTCATCACAG

TCAAGGTCACCACAGCACAACCAAAGAATATTTGTGCCAAGAAGAATGTGCCTACTTCAC

CAAGGCCACCCCCAAGTCCTTATTCCTTTCCTGTGACTTCAGCTTTCCAGAACTTTTTTC

CATCTCAACCATTTCTCTCCCCACTGAGTAATTCTAACTGGGCTCAGAACTCTGCCTTTA

CTTGTAATGATGCAGGTAATGACCAAATGCATGATCCACAGTTGGAGAGCCTAGTAGCTT

CAGTTACTGCTTTGTACTCTAGTGACATAGCTCAACTCCTAGAACTATATAGTTTTCCTG

ATGAGGATCCACTATCTAGTTCTTCCTTCAACTGTCTGTATCATTATCTTGGCCCCAATA

GG

>Armadillo-ARGFXP1

NNNNNNNNNGAATCCCCAGCCCTGATGCCTTCTGTCAACAGGGAATGATGCCAACATGAA

TTTGATACCACAGAATCCAGCTGAATATCATACCCCTGATGAACAGAATGTCTCTCGAGA

TTGCCCGGCAAGACTCTTTTATCATTATGGGAGTTTAAGCATTTACTTGATAGCTTAGGA

TCAAGCTGATTCCTCAATGTGGAGGAAACATTATGATCATACCTCATCTCCCACAAACAG

CATGAAGAGTTGGAGGCCCTATTTAGCTAGACCATGTTTCTAGATAAAAATATCTGTAAG

AAACTAACTTTGAAACTCAACATATGGAGTCAAAATTGAGGAAGCAACAGTGATCACTAA

AGAAATCAAGCCAAATCCTTCCAGCCAAGGAGAATGCGCTCACCTCACCCAGAGTAAGCA

TGAATCTTTATTCTTTTGTTCGCTGTGGTTTCACATTCCTACAGCTCCCTTCCACATCAG

CCATTAAGTGTATCCATATGGGTATGGGACTCTGTCTTCTGTGGGAATTCCACCAGTGTT

AGGCAAATGCAAGTTCCACATTTGGAAGGGCTTATGGCCTCAGTTCCTTCTTTGTACTCT

GATGCTTATGGCATATCCCAAATTATGGAATTGTAGTATTCCTGATGAGAATGAGATATA

TAGTTCTTCCTTCAACTATCTGTATCAGTATCTCTTACCCACAAGACCTCAGCTGGAATG

ACAGGATAATACTCTCAACAATTTTGCTGGTCCAGTTGTAAGTCTCTCTCCACAGTAAAA

CTGGTCCAGTATGACAAGCCAGTGCTTTGATGCCTACCATGTAAGATACAGCCTGGAATT

CCAGTACCCTGCCAGTATGGTGGAATATGAATTTCCATATTAGAGTACTAATAAATACCA

GGCTTTTCACAAGTAGGGTCTTCCTACATCTTGTGAAAAAAAAGTAGGACCTTGTAACAC

CCTGGCTTGCCTGTCCCAATTCCCCTTACCACTTGGCACAGAGCCAGTCCACAATCACAG

TGTGACTTATTCCTAGGGATGTGGATGATTCATCACAGTATCTACCATCATAAACCCAAA

TGTATTAATTACATTAAATATAAATGGACCAAATGCTTCAATTAAAAAACAAAGATTGTC

AGACTATATTAAAAAATCACAATCTAGATATATTTACACACGACACATAAAAAATATAAA

GGTGCACATAGTTTGAAAGTTAAAAAATTAGGAAAAGGCTTGTGAAAATTGTAAATATAG

GGATTTGAGAGAGGGAAAATGGCATTAAATATATATTATATGTGTATATGTCTGTGTATT

CATTTAAGTTTCACATTACACAAACTGTGCCTCTTTTTAAAGTCTGAATCAGCCCTTGCT

GGCATTAAATGTAATATATTTTAATTAGTTTCTCCTTGTTTCAGTTTTTCAATGTTTGCC

TAAATAATTGCCTTGCATTTGCACAAATTATGTCAATATCCACACATAGTTATTCTGGTC

CTAATCATTTCTGTTTTGAAATACAGAGTCTAAACTGATTGTCTCTTTCCCTCTGCATTA

CTGCATGGATTATCATTTTAAGTGGGCTGAATCAAACCAAAGCATATAAAAAGATTGCTG

ATTTTTAAAATTGGGGTATATAAATATGAAACTGCATTATCTCAAAACCACATTAGCAAA

AACATTTACAATATCCTATGTGTAAGCTTTGAGATTTCCTTCAAAATCCTCAGTCAGTTT

CATCTTTGTGAACTAAATATTATTATTAAAAGACACAGAAGATACAAAAGAGGCTCTACA

GATATTTTTTTTCTTAAGTTTAACAGTGTGGGCTCTTTATAATAATAGATTTTCTTCCCT

TTGAAGTTGGCAGTGCTGCTTTTAACTTTTTCGTAGTTCTTTTTTTCTTATTTTCAATCA

TTTAAATTTTATATTAAATGTAATTACTATCATAAAGCAATCATATAAAAAGAATAATCT

ACTTTAGAGGAGGTATGTCAGTTAACAGATAATTCCTGGTCATCTACTATGTACCAGTCA

TTATCTACCTGCTGGAGATAACCAGAATCTCTGCCTTAAGGAGATAATATATACTCTTGT

AGTGTAATACATATTATATGACAAAAACAAACCTAAATAAAT

>Armadillo-ARGFXP2

NNNNNNNNNNNNNNNNNNNNNNNNNNNNNNTAGGTATATAATGTAACTAGATTTATTGTT

AACTTTTACCAATCAATTGATGGATAAAGCACAGAAGAGCTGGTTCTGGCCGGAGGCTAG

AACGTAAATGTTATCCTTGACCATGTGAAAATAAAACAGTGTAGGTGACAAACAGAAGGC

TATGGAAGGGTGGGTAGAGAAGAGGGCAGGGAAGTTAGTATCTTCAGCTTATAAAATGGG

AGTCGTGATATTGCTGAAAATAGATATAACAAGAAATAGACGTTTACATGATTTACCTTT

ACAAAGACAACAAACAGATATATTAAAATAATAATTTAACATCAAACGTGAGAAGAGGGG

ATGTAAGTAATGAGCTAAGCCTCATCTGTCAGGTCAAGGAATCAATGGAAGGCATCAATT

TGATAAATCAAGAGATAGAGGAATAAATATACATTATTTAGAACACTTATTTAGAACAAT

CGTTCCCAACTCTGACTGAACATGACTATAAGCTTGGAGACTTAAAAAAAAAAAAAGCCA

CCAGAACACATGAAACTGCATTTCCAGAAAGAGATAGATCAAGCAGGACTGAAAATGGAC

ATTTCAAAAGGATCTGTGACAGCCTGCCGGTGCCCAGATGGACAGAGCTGCCCCCGGGAA

TCCCCGGTCCCACCCCTTTACCCAAATGGGTGATTCCAGCATGAACTTGATCCCATACCC

TAATGAACAGAAGGTCCCCTGTCACTCACAGCCCTTTTATCAATATGGATGCATCAGGCA

TGTACTTGGTACCTCAGGATCGAGCCAATGCGCCAACGTGGAGGAAGCCTAATGATCATA

CCTCATTCTCCCACAAACAGCAGAAGAACTGGAAGCTCTACTCAGGCAGCCCATGTTTCC

AGATAAAAATATCCAGAAGGAACAGGCCTTGAAACCCAACATGGAGGAATCAAGAGTGAA

GTTCTCGCTCAGGAATCAACGATTCAAGCAGCAGCAGTCACTAAAGAAATCAAGCCAGAT

CCTTCCAGCCAAGGAGAATGTGCTCCCTTCACCCAGAGAGAGCACCAATCCTTACTCTTT

TTTCCTGTGGTTTCACATTCCTACAGCCCCCTCCCACCTCAGCTATGAGGCACTTCCAAT

TGGGCATGCGACACTGCGTTCCCTGGGAATCCCATAAACAACATTCAAATTCAAGTTCCA

CGGTGGAAAGGCTTGTGGCCACAGTTCCTGTTTTGTACTCTGATGACTATGACGTATCCC

AAATCATGGAATTGGAGTTTTCCTGATGAGAATGAGATAGTCAGCTCTTTTTCAGCTGTC

CTATACCAGTATCTCTCACCCATAAAACCTCGGTTACAAGGACAGGGTGACACTCTCAGC

AACTTTGCTAGTCCAGCGTGAACCTGGTCCAGCATGGCAACCCAAGGCTTTGATGCCTAC

TGTGTAAGACACAGCCTGGAATTTCAGTGCCTCTCCAGTATGGCGGACTGTGAATTTCTA

TATCAGAGGGCTGATAAATACCAGGCCCTACAGAAGTAGGGTCTTCCCACATCTTGGAGA

AAAAAAAAAATTAGTTGGTATGGGAATGAAACCACACATAGGTATCATATAAAAAACTCC

CAAGGTGATTTTAACATGCAGCCAGGGTTGAGAATCACGGACCTAGAAAAACAGAAAACA

AAGGCAGGAACAGATCAGTGAGAAGAGAGAGAGGAGGTATAAAAAGGAAAGGCTAGACAC

TGTGACTTTGAATTCATTCATTCATTCATTCGTTCGTTCGTGTTTTTCAACAAATGTTTA

TGAGAGAGCCTTCTATATGCCAGGCCCTTTTTCATCATAAACTCCCCATTACTAGTTGAT

TTGTGTTCACAGAGATGAGTTGCTTCAATTAAAAAAAAAAAATTAATGCAACAAAAATAA

AGCTTCACTTACTAAATCCACACAAATACATTGGGCAACTAGTTGGAACCACTTCTGCCT

GAGTGGAAAGGGCATCACCTGCACAAGATAATAACCATGGGGAAAGAACACCTTTCACAA

AGGGCAGCCTGGCTACAAGCTGATGAGGAGCAGATTAAAGGAGTGTCTGAGGGCTCTGGG

GAGTACCCACTCCACTTTCACCACAGCTGGGTTCACAAGCAAAGGACTCAAAGCTCCAGG

ACCCTAAAAAAGAGTGAAAAGTGGGAGGGAGAAGACAGGGTGGAACAAAGGGGCCCCAGA

GGGGAAGTCCGTAAGAGTAAGTGAAAGAAATGGCCCCTTGATCAAAAGTGCAGCCCTACT

ACCTGTCATTGCAGTTCTCTGTTGTTTTCAAGAGTATCTTGAAACGTAACAGATATTAAT

GAGATTCTCGCCAACAAGCTACTTTGAAATAACACCTTAATGGTTAGTAGACACCCCAGC

ANNNNNNNNNNNNNNNNNNNNNNNNNNNNNNNNNNNNNNNNNNNNNNNNNNNNNNNNNNN

>Armadillo-ARGFXP3

NNNNNNNNNNNNNNNNNNNNNNNNNNNNNNNNCACTTTCTCCTCTAGGATTCACCAGGAT

TCGATCTTGGGGAGCTCTGATATGGAGAGAGGTTCCCTATCAATAGTGCCACCTCAGTTC

CTGGTCTCCGTGGCACTTCACCTTGACTCTCCCCTTCATCTCTCTTTCATTGCATCCACA

TCTTGCTTTGTGACTCACTTGTGTGGGCAGTGGCTCACCACATGGGCACTCAGCTTGCTG

CGTGGGCACTGGTTTGCCACGCAGGCACGCTTCTTCTTCTTCTTTTACACCAGGAGACCC

CAGGGATTGAACCCAGGTCTTCCCATATGGTAGACAGAGGCCCTATTACTTGAGCCACAT

CCGCTTCCGAAAGCCCTAATCTTGATTTAATCAAGTAAACATAAAGCCTTTGGATTTAGA

TGAATCAAAAGGCATTGTGCCCAGAGGAACAGACCAGTTTATAAACAGTATCAATACCTC

TTTTTGCAATTCATAAATAATATCAAACTGCCACATCTTTCTAACTTATTTGTATTTAAA

TTTTATATAGATGAAGTCAGACAATATGTAGTACCTTTTGTCTAGCAGGACAGTGCAAAC

AGATGCACAAGAAAGTGGATATCCCAGTCCGACTGGTTCAGGTAGCAGAGGATGGGAGAT

AAGGCTGGAAAATTAGGTTGGGACCACATGACAGAAGGACTTAAACACCTGGCTAAGCAA

TTTGCAATTTATTATATAAGAATTGAGATTTGTTAAAGGTCTATAGCCTTGGGAAGCAAT

TGAGCTCCCGCCTACCACATGGGAGGTCCCAGATTCAGCTCCTGGTACCTCTGAGGAATG

GAGCTGATTCCTCAACATGAAGGAAGCATTATGATTGTACCTCTTTCTCTCACAAACAGC

ATGAAGAGTTGGAGGCTCTATTTGGCCAGATCACATTTCAAGACAAAAACATCCAGAAGG

AACTGGCTTTATAACTCAACAGGCAGGAATCAAGAGTGATGGTTGGTTCTGGAACTGGGG

ATTCAAACTGAGGAAGCAGTGGCAACAATAGCAGCAATCACTAAGGAAATCAAGCCAGAT

CCTTCCAGCCAAGGAGAGCAAGCATCGGTCCTTTTTTTCCTCCCAGTGGTTTCTCATTCC

TACAGCTCCCTTTCATCTCAACCATTAGGTACTTCCAGTTGGGTGTGGGACTCTGTCTTC

CCTGAGAATCCCATCAGTGACATCCAAATGCAAGATCCACAGTTGGAAAGGCTCGTGGCC

TCAGTTCCTGCTTTGCACTCATGCAAAACATGGAATTGCACAGGCTTCCTGATAAGAAAG

CTCATCCTTCAGCTATCTATACCAGTATCTCTCACTGGCAAGACAGCAGAGAGGACAGAG

TGACACTCTTGGCAACTTAGCTGGTCCAGCTGTAGGTCTATCTTCAGAGCAAATCTGGTC

AGCACAACATGCTAAGGCTTTGATGCCTGCCGTGTAACATACAGCCTGGAATTCCAGTTC

CGCTCCAGTACGGTGATTTCTATATCAGAGTATTAATAAATACCAGGGTCTTCCCATCTC

TTAAAAAAATAGGTGGTGAGATCATTTGCTGAGGCAGGAAGACAGTGAGGGAGGAACCGT

GGAGCTTGGAAATGTAGTTTTGGATTTGGTCACATGGGGTGAGTGTGTGGACTTAGGTAA

TTAGAGGGTTGCAGACAGAATGCTTTCACCATCTTGGTGTAAATGATCCAAATCTCTGAA

TTTCTTTCTTTTTTCTTTTTGTACACTTAGCTGCCTGAATTATTACATACTTACCACATT

TCATTTTTTCTGGGTAAGAATAAGAGATAACCACTGTGAGATCATTTTTATCAAGAATGT

TGATTCTTTTCTTTCACAGAAACCAAAAGGGATCATTTATTTATTATGAGGTATTTGCTA

GCTTCACTCAAAAGATATGAGGTTTTTACATTGTTGGTTTTGAGATTATCTCTATTTTTA

TACACTAAAGCAGCTAGTGTTCCTGACCGCATCTTGGGCAAATGCCAGGTTGGGGGATTA

GGCTAACCAACTCTAATCAGGCTTCCTGAACTTAATCAGTGAATGGGATTAGCTGTTCAT

TTGATAGTTTATCTGAGCCATAAGANN

>Armadillo-ARGFXP4

NACTCACAATTCTTTTTGACATAAAAGCAATCATTTAGTTCTGTTTTTTTAAAGAAAGAC

ACACAGGTTTCATTAGTGTGTTTAAAAATCAAGTGCAGACTGTTGGCCATGAGGACACAC

TGAAGAAACTGCATTTCCAGAGAGAGACACATCAAGCAGAATCAAAAATGAACACTTCAG

AGGAATTCATGATAGACCTACTAATGGAGCTTGCTGGTGGCCAGATGGACAGAGTGGTCC

CTGAGAATCCCCAGCCCCGATCTTTAATCCAAATGGGTGATCCAACATGAACATAACACA

AGACCAAGTGGATCACAGCCTTCCATCCCAGATTTCAGGATTTCCAATGAACAGAATGGC

CCCAGGGAATCCCCAGCCTCACCCTTTTGTCAGTATGGGTGATTCCAACATCAACTTGAT

ACCACAGAATCCAGCTGATCCCACATCTGTGATGAACAGTATGTCCCTTGAGACTCCTCA

GTCAGAACCTTTTATCAATGTGGGTTATTCAAGCACATGTTTGGTACCTCAGGATCAAGC

CAATTCCTTAGGAAGAGTTGAAGGCTTTATTCAGGGAGACCATGTTTCCAGGTAAAAATA

TCCGGAAGGAACTGGCTTTGAAACTCAACATAGATGAATTGAGAGTGAAGGTTTGGTTCA

AGAACCAGTGATCCAAGCTGAAGAAGCAGCAGCAACAATGACAGCAATCACTAATGAACC

CGAGCCAGATCCTTCAAGCCAAGGAGAATGTACTCTCCTCACCCAGAGCAAGCACCAATC

CTTATCCTTTTTTTCCCGTGGTGTCACATTCCTACAGTTCTCTTCCACCTCAGCCATTAG

GCACTTCCAATTGGGCATGGGACTCTGTCTTCCCTGAGAATCCACATATAGTATCCACAG

TTGGAAAATCTTGTGGCCACAGTTCCTGCTTTGTTCCCTGATGATTATGACATATCCCAA

ATCGTGAAATTGTACAATTTTCCTGATGATGAGACTTCCAGCTCTTCCTTCAGCTGTCTA

CATCAATATCTCTCACCCAAAAGACCTCAGCCAGAAGAACAGGGTAACACTCTCAGCAGC

TTTGCTGGTCCAGCTGTAGGTCTGTTTCCAGAGCAAACCTGGTCCAGTGTGACAAGCCAG

GGCTTTGATGCCTACCGTGTAAGATATACCATGGAATTCTAGTACTCCTCCAGTGTGGTG

TACTATGGATACACATATATCAGAGTACTAATAAATACCAGGCCTTACAGAAGTAGGGTC

TTCCCACCTCCTACACATACCTCTGTTCTTTCCTTTGACTACTTTTTTTTTTTTTTAAAG

ATTTATTTATTTATTTAATTTCCCCCCCTCCCCTGGTTGTCCGTTCTTGGTGTCTATTTG

CTGCGTCTTGTTTCTTTGTCCGCTTCTGTTGTGGTCAGCGGCACGGGAAGTGTGGGCGGC

GCCATTCCTGGGCAGGCTGCTCTTTCTTTTCACGCTGGGCGGCTTTCCTCACGGGCGCAC

TCCTTGCGTGTGGGGCTCCCGCACGCGGGGGACACCCTTGCGTGGCACGGCACTCCTTGC

GCGCATCAGCACTGCGCATGGCCAGCTCCATACGGGTCAAGGAGGCCCGGGGTTTGAACC

GCGGACCTCCCATGTGGTAGACGGACGCCCTAACCACTGGGCCAAAGTCCGTTTCCCTGA

CTACTTTTTAAACTCAGATATCTGGGTAAGTGTCCCTGTGATTTCCGTGAAAATCTTGTA

AAAACTAGAAATTTTCTAAGTATAGCTTAGCACCACACAATCAGCCCGCACAGCTCTCCC

TGGGGAAGGCCTTCCTGGTCCCTCAAGTGGCCAGTGAGACTCTGAAATGTCCTCTTCCAA

AATAATATCTTATACTTCCCTGAAAAAAAAAATAAAGTGCACTGTACTGTACTATGTAAG

CAGCTTTTAAAATGTGGGCTTAAGATGCTCTCCCCTAAGCTAAGAACTTCTCCTTTTATC

TTGTAAGACTAGCATAGCTGGCAAGGCTGATTTGTGCTAGTGAGAAATCTCACTAGACCC

TCAGTCCTCCTGAGGAGATCAACCAAGAN
