# Supplementary material for: The origin and evolution of ARGFX homeobox loci in mammalian radiation
Source: BMC Evol Biol. 2010 Jun 17;10:182. doi: 10.1186/1471-2148-10-182 (PMC2894831; doi:10.1186/1471-2148-10-182)
Supplement: Additional file 2 — Alignment of eleven ARGFX coding sequences showing mutations leading to frameshifts (in grey shade) or stop codons (in red shade). The homeobox is underlined. [file 1471-2148-10-182-S2.PDF]

```
Human-ARGFX        ATG AGG AAC AGA ATG GCC CCA GAG AAT CCC CAG CCA GAC --- CCT TTC ATC AAT AGG AAT TAT TCC AAC ATG AAG GTG ATA CCA CCA CAG  [  90]
Chimp-ARGFX        .C. ... ... ... .G. ... ... ... ... ... ... ... ... --- T.. ... ... ... ... ... ... ... ... ... ... ... ... ... ... ...  [  90]
Orangutan-ARGFX    ... .T. ... ... ... ... ... ... ... ... ... ... ... --- ... ... ... ..A ... ... ... ... ... ... ... ... ... ... ... ...  [  90]
Macaque-ARGFX      ... .T. ... ... ... ... ... .G. ... .G. ... ... ... --- ... ... ... ..C ... ..A G.. C.. ... ... ... A.. ... .A. .TG ...  [  90]
Marmoset-ARGFX     ... .T. ... ... ... ... ... ... ... ... ... .A. ... --- ... .C. ... ..C ... ... G.. ... .G. ..T ... ... ... T.. ... ...  [  90]
Mouse_Lemur-ARGFX  --- .T. ..G ... .CA A.. ... ... .G. ... ... ... ... --- ... .CT ... ..C .T. ... G.. ... ... ... ..C T.. ..G TT. ..T ...  [  90]
Tree_shrew-ARGFX   CC. .T. ... ... ... ... ... ... ... .TT ... GA. ... --- T.. .CT C.. ..C .T. ... G.. .TG C.. ..A ..T T.. ..G ... --- ...  [  90]
Guinea_Pig-ARGFX   ..C TT. ... ... .G. A.T ... ..A ... ..T GG. ... ... TTT GT. ... T.. G.. CA. ... .TC ... .GA TG. ..C TC. T.G G.. ... .C.  [  90]
Cow-ARGFX          C.. .T. ... .A. .GA .T. .A. ... ... ... .T. ... ... --- ... A.G ..T CTA GCA CGA AC. ... .GG ... .T- --- --- --- T.. A..  [  90]
Horse-ARGFX        C.. .T. ..T G.. ... ... ... ..T ... ... ... ... ... --- .T. ..T ... ..C TT. G.. G.. ... .G. ... ..C T.. ... ... .AG G.T  [  90]
Megabat-ARGFX      --- .T. ... ... GG. ..T T.T .G. .C. ... ... A.T C.. --- T.. ..T T.A C.C .T. .G. A.. .A. .G. ..T G.C A.. ... .A. GA. A.A  [  90]

Human-ARGFX        GAT CCA GCT AGT --- --- --- CCC AGT TTC ACT CTG TTA TCC AAG CTG GAG TGC AGT GGC ACG GTC TCG GCT TAC TGC AGC CTC AAC CTC  [ 180]
Chimp-ARGFX        ... ... ... ... --- --- --- ... ... ... ... ... ... ... ... ... ... ... ... ... G.. ... ... ... ... ... ... ... ... ...  [ 180]
Orangutan-ARGFX    ... ... ... ... --- --- --- ... ... ... ... A.. ... ... C.. ... ... ... ... ... ..A ... ... ... ... ... ... ... ... ...  [ 180]
Macaque-ARGFX      ... ... ... ... --- --- --- ... .T. ..G CTC T.A ..G C.. .G. ... ... ..G .A. ... G.. A.. ... .A. C.. ..T .A. ..A C.. T..  [ 180]
Marmoset-ARGFX     ... ..C ... ..A TAT CAG CAG GAA G.. ... .T. ... .C. ... C.. .AT ... ..T G.. ... G.A ... ... ... C.. ... ... ... ... ...  [ 180]
Mouse_Lemur-ARGFX  ... ... ... ..G --- --- --- ... nnn nnn nnn nnn nnn nnn nnn nnn nnn nnn nnn nnn nnn nnn nnn nnn nnn nnn nnn nnn nnn nnn  [ 180]
Tree_shrew-ARGFX   ... ... ..A ... --- --- --- ... --- --- --- --- --- --- --- --- --- --- --- --- --- --- --- --- --- --- --- --- --- ---  [ 180]
Guinea_Pig-ARGFX   TG. ..T .G. G.A --- --- --- .T. --- --- --- --- --- --- --- --- --- --- --- --- --- --- --- --- --- --- --- --- --- ---  [ 180]
Cow-ARGFX          ATA ... ... G.. --- --- --- G.. --- --- --- --- --- --- --- --- --- --- --- --- --- --- --- --- --- --- --- --- --- ---  [ 180]
Horse-ARGFX        CCA --- ... GA. --- --- --- ... --- --- --- --- --- --- --- --- --- --- --- --- --- --- --- --- --- --- --- --- --- ---  [ 180]
Megabat-ARGFX      .T. G-- ATC .CA --- --- --- G.G --- --- --- --- --- --- --- --- --- --- --- --- --- --- --- --- --- --- --- --- --- ---  [ 180]
```

```
Human-ARGFX        CCA GGT TCA ACT GAT CCT CCC ACC TCA GCC TCC CGA GTA GCT GCG ACT ACA GCA ATA CGG AGA AGG CAT AAA GAA CGT ACT TCT TTC ACC  [ 270]
Chimp-ARGFX        ... ... ... ... ... T.. ... ... ... ... ... ... ... ... .G. .A. ... ... ... ... ... ... ... ... ... ... ... ... ... ...  [ 270]
Orangutan-ARGFX    ... A.. ... ... ... ... ... ... ... ... ... ... A.. ... .G. .A. .G. ... ... ... ... ... ... C.. ... ... ... ... ... ...  [ 270]
Macaque-ARGFX      TT. .AA ... .G. ... T.. ..T G.. ... ... ... .A. A.. ... .G. .T. ... ... .C. T.. ... ... ... C.. ... ... ... ..A ... T..  [ 270]
Marmoset-ARGFX     ... ..C ... .G. ... ... ... G.. ... ... ... .A. .C. A.. .G. ... ... ... .C. T.. ... ... ... C.. .CC ... ... ..A ... ...  [ 270]
Mouse_Lemur-ARGFX  nnn nnn nnn nnn nnn nnn nnn nnn nnn nnn nnn nnn nnn nnn nnn nnn nnn nnn nnn nnn nnn nnn nnn nnn nnn nnn nnn nnn nnn nnn  [ 270]
Tree_shrew-ARGFX   --- --- --- --- --- --- --- --- --- --- --- --- --- --- --- --- --- AGT GCG T.. .AG .AA ... C.. ... ... ..C ATA ... ...  [ 270]
Guinea_Pig-ARGFX   --- --- --- --- --- --- --- --- --- --- --- --- --- --- --- --- --- AAT GCC A.. ..G .A. ..A C.. .GT ..C ..C ..A ... ...  [ 270]
Cow-ARGFX          --- --- --- --- --- --- --- --- --- --- --- --- --- --- --- --- --- A.. .A. T.. .AG .A. AG. C.T ... ..C ..C ..A ... ...  [ 270]
Horse-ARGFX        --- --- --- --- --- --- --- --- --- --- --- --- --- --- --- --- --- A.. .C. TA. .AG TA. ..C C.. ... T.C ..C ..A ... ..T  [ 270]
Megabat-ARGFX      --- --- --- --- --- --- --- --- --- --- --- --- --- --- --- --- --- A.. ..G T.. .AG .A. ..C CC. A.. T.C ..C .TG ... .TG  [ 270]

Human-ARGFX        CAC CAA CAG TAT GAG GAG CTA GAA GCT CTG TTT AGC CAG ACC ATG TTC CCA GAT AGA AAT CTT CAG GAG AAA CTA GCT TTG AGA CTC GAC  [ 360]
Chimp-ARGFX        ... ... ... ... ..A ... ... ... ... ... ... ... ... ... ... ... ... ... ... ... ... ... ... ... ... ... ... ... ... ...  [ 360]
Orangutan-ARGFX    ... ... ... ... ..A ... ... ... ... ... ... ... ... ... ... ... ... ... ... ... ... ... ... ... ... ... ... ... ... ...  [ 360]
Macaque-ARGFX      .G. ... ... ... ..A .C. ... ... ... ... ... ... ... ... ... ... ... ... ... ... ... ... ... ... ... ... ... .A. ... A..  [ 360]
Marmoset-ARGFX     .G. G.. ... ... ..A ... ... ... ... ... ... ... G.. ... ... ... ... ... ... ... .G. ... ... ... ... ... ... .A. ... A..  [ 360]
Mouse_Lemur-ARGFX  nnn nnn nnn nnn nnn nnn nnn nnn nnn nnn nnn nnn nnn nnn nnn nnn nnn nnn nnn nnn nnn nnn nnn nnn nnn nnn nnn nnn nnn nnn  [ 360]
Tree_shrew-ARGFX   ..A A.G ... C.. ..A ... T.G ..G ..G ... ... ... ... .AT ... ..T ... ... .A. .T. ..C ..A A.A G.. ... ... ... .A. ... A..  [ 360]
Guinea_Pig-ARGFX   .G. ..G ... C.G A.. ... ..G ..G ... ... ..C ... ... ... ... ... ... .CG .AG ... G.C .G. ..A G.. ..G ... GGA ..G ... A..  [ 360]
Cow-ARGFX          ... AC. ... ..C A.. ... T.G ..G ... ... ... ... TGC .A. ... ..T ... ... .A. ..C ..C ... AGA G.. ..T ... ..A .A. ... A.T  [ 360]
Horse-ARGFX        ... AG. ... C.. ..A ... T.G ..G ... ... ... ... .-- ... ... ..T ... ... .A. ... ..C ... A.. G.. T.G .A. ... .A. ... A..  [ 360]
Megabat-ARGFX      ..T AC. ... C.. ..A ... T.G ..G ... ... ... ... ..C ... ... ..T ... ... .A. ... ... ... A.. G.. T.T ... ... .A. ... A..  [ 360]
```

```
Human-ARGFX        CTA CCG GAG TCA ACA GTA AAG --- GTT TGG TTC AGG AAC CGG CGA TTC AAA TTG AAG AAG CAG CAG CAG CAG CAA --- --- --- --- ---  [ 450]
Chimp-ARGFX        ... ... ... ... ... ... ... --- ... ... ... ... ... ... ... ... ... ... ... ... ... ... ... ... ... --- --- --- --- ---  [ 450]
Orangutan-ARGFX    ... ... ... ... ... ... ... --- ... ... ... ... ... ... ... ... ... ... ... ... ... A.. ... ... ..G CAG CAG CAG CAG CAG  [ 450]
Macaque-ARGFX      ..G ... ... ... ... ... ... --- ... ... ... ... ... ... ... ... ... ... ... ... ... ... ... ... ..G CAG CAG CAG CAG CAG  [ 450]
Marmoset-ARGFX     ... T.. ... ... ... ... ... --- ... ... ... ... ... ... ... ... ... CA. ..A ... ... ... ... ..A ..G CAG CAG CAG CAG CAG  [ 450]
Mouse_Lemur-ARGFX  nnn nnn nnn nnn nnn nnn nnn --- ... ... ... ... ... A.A ... ... ... .G. ... ... ... A.. ... ... ..G CAG CAG CAG CAG CAG  [ 450]
Tree_shrew-ARGFX   ..G .AT ... ... ... ... ... --- ... ... ... ..A ... A.. A.G ... ... C.. ... ... ... ... ..A ..A ..G CAG --- --- --- ---  [ 450]
Guinea_Pig-ARGFX   ..G GA. ... AA. ..C ... ... --- A.C ... ... C.. ... A.. ..T ... ..G C.. .G. ... ... ... A.. ... G.G GAG CAG --- --- ---  [ 450]
Cow-ARGFX          ... ..A ... ... ... ... ... --- A.. ... ... ... ... ... ..G ... ... A.. ... ... ... ... .G. G.. ... GAG CAG CAA --- ---  [ 450]
Horse-ARGFX        ... ... .G. ..T ... ... ... --- AC. ... ... ... ... G.. .A. ... ... C.. .G. ... ... ... ... ..A ... CAG CAA --- --- ---  [ 450]
Megabat-ARGFX      ... ..A ... ... ... A.. ..A TCT AC. ... ... ... ... .A. .A. G.. ... ... .G. ... ... ... A.. ... .GG CAG CAG CAG TGG CAG  [ 450]

Human-ARGFX        --- --- --- --- --- --- TCA GCA AAG CAA CGA AAC CAG ATC CTT CCA TCC AAG AAG AAT GTG CCC ACC TCC CCC AGA ACA TCC CCC AGT  [ 540]
Chimp-ARGFX        --- --- --- --- --- --- ... ... ... ... ... ... ... ... ... ... ... ... ... ... ... ... ... ... ... ... ... ... ... ...  [ 540]
Orangutan-ARGFX    CAG CAG CGG CAA --- --- ... ... ... ... ... ... ... ... ... ... ... ... ... ... ... ... ..T ... ... ... ... .T. ... ...  [ 540]
Macaque-ARGFX      CAG CAG CAG CAG CAA --- ... ... ... ... ... ... ... ... .C. T.. ... ... ... ... ... ... ... ... .T. ... ..G ... .T. .A.  [ 540]
Marmoset-ARGFX     CAG CAA --- --- --- --- ... ... .T. .T. .C. ... ... ... ... .T. ... G.. ... G.. ... ... ... .T. .TG ... ... ..T AT. .T.  [ 540]
Mouse_Lemur-ARGFX  CAA --- --- --- --- --- ... T.. ... ... .C. ... ... ..T ... T.G A.A --- --- ... A.. ... ..T ..A T.. ... .T. .T. A.. CA.  [ 540]
Tree_shrew-ARGFX   --- --- --- --- --- --- ... CA. ... ... .C. ... ..C ... ... ... G.. .T. .G. .T. A.. ... ... ..A ... ... ... .G. A.A .AC  [ 540]
Guinea_Pig-ARGFX   --- --- --- --- --- --- ... CTG C.. ..G TC. ..G ... .CG .C. ... C.T GT. ..C .G. ... ..T T.. ..A .T. ... G.. ... G.. .AA  [ 540]
Cow-ARGFX          --- --- --- --- --- --- ... CT. ... .C. .C. .G. ... G.. ... ... G.. ... --- G.. ... ... --- --- --- .C. GT. ..A A.. ..C  [ 540]
Horse-ARGFX        --- --- --- --- --- --- ... CT. ... ... .A. ... ... ... ... ... G.. ... --- .CG ... ... .A. ..A ... .C. G.. ..A AG. ...  [ 540]
Megabat-ARGFX      CAG CAG CGG CAA CAG CTA ... CT. ... ... .C. ... ... ..T ... ... G.. C.. --- ... ... ... T.. ..A ... .C. ... ..A A.. ...  [ 540]
```

```
Human-ARGFX       CCT TAT GCT TTT TCT --- CCT GTG ATT TCA GAT TTC TAC AGC TCC CTT CCA TCT CAG CCC TTA GAC CCT TCC AAT TGG GCA TGG AAC TCT  [ 630]
Chimp-ARGFX       ... ... ... ... ... --- ... ... ... ... ... ... ... ... ... ... ... ... ... ... ... ... ... ... ... ... ... ... ... ...  [ 630]
Orangutan-ARGFX   ... ... ... ... ... --- ... ..A ... ... ... ... ... ... ... ... ... C.. ... ... ... ... ... ... ... ... ... ... ... ...  [ 630]
Macaque-ARGFX     ... ... ... ... ... --- ... ... ... ... ... ... ... ... ... ... ... C.. ... ... ... ... ... ... ... A.. ... ... ... ...  [ 630]
Marmoset-ARGFX    T.. ... .A. ... .A. --- ... ... G.. ... ... ... ... ... ... ... ... C.. ... T.. ... .G. ... ... ... ... ... ... ... ...  [ 630]
Mouse_Lemur-ARGFX ... ..C T.. ..A .T. --- ... ... G.. ... .G. ... .GT ... .A. G.. G.. C.. ... T.T ..G ..T ... ..T ... ... ... ..T GGG .G.  [ 630]
Tree_shrew-ARGFX  ... C.. TT. ... C.C --- ... ... G.. ... ... ... ..T ..T ... .C. ... ... ... ... ... ... ... ..A ... G.. ... ... GG. ...  [ 630]
Guinea_Pig-ARGFX  G.A C.C TAC C.C A.. --- T.. C.A GC. ... ... ..T G.T G.. ... ..A T.. AGG .G. .A. AC. A.. ... --- --- --- --- ..T G.. ..A  [ 630]
Cow-ARGFX         ... C.. T.. ... CTC --- .T. .CA ... ... ... .C. ..T .A. ... ..C T.. C.. ... ... ... ... A.. .T. CCC ... ... G.. G.. ...  [ 630]
Horse-ARGFX       ... .G. T.. ... GT. T-- ... .CA G.. ..C ... ... .GT ... ... T.C .-- A.C TCA G.. C.T AGG ..C .T. CC. ... ..C ... G.. ..G  [ 630]
Megabat-ARGFX     .TA ... T.. ... .T. --- ... A.A G.. ... ... ... ..T ... ... ... ... C.. ... ... ... .G. ... C.. ..C ... ..G ... G.. ...  [ 630]

Human-ARGFX       ACC TTC ACT GAG AGT TCT ACC AGT GAC TTC CAA ATG CAA GAT ACT CAG TGG GAG AGG CTG GTG GCC TCG GTT CCT GCT TTG TAC TCT GAT  [ 720]
Chimp-ARGFX       ... ... ... ... ..C ... ... ... ... ... ... ... ... ... ... ... ... ... ... ... ... ... ... ... ... ... ... ... ... ...  [ 720]
Orangutan-ARGFX   ... ... ... ... ... ..C ..A ... ... ... ... ... ... ... ... ... ... ... ... ... ... ... ... ... ... ... ... ... ... ...  [ 720]
Macaque-ARGFX     ... ... ... ... ... ..C ..A ... ... ... ... ... ... ... ... ... ... ... ... ... ... ... ..A ..A ... ... ... ... ... ...  [ 720]
Marmoset-ARGFX    ..T ... ... ... ... C.C ..A ... ..T ... ... ... ... ... ... ... ... ..A .A. ... ..A ... ..A ... ... ... ... ... ... ...  [ 720]
Mouse_Lemur-ARGFX G.. ... ... ... ... C.C ..A .A. ..T ... ... ... ..G ... CT. ... .T. ... ... ... ... ... ..T ... ... ... ... .T. C.. ...  [ 720]
Tree_shrew-ARGFX  GT. ... ... ... ... C.C ..A ... .GT ... .C. ... G.. ... C.. --- --- --- --- T.. ... ... ..A ... ... ... ... ... ... ...  [ 720]
Guinea_Pig-ARGFX  T.G GAG GA. ... ... C.C ..A .C. A.T ... ... ... .GT ... TT. ... .TA ..C .A. ... .A. ... ..T ... .G. ... G.. .GT ..C ...  [ 720]
Cow-ARGFX         .TG A.. .TG ... .T. C.. ..A ... ..T G.. ... .C. ... ... C.. ..A CT. ... ... ..A ... ... ..A ... ... ... ... ... ... ...  [ 720]
Horse-ARGFX       .T. A.. ... ... ... C.C ..A ..A ..T G.. ... ... ..G ... CT. ... .T. ... ..A ..A ... ... ..A ... ... ..C ... ... ... ...  [ 720]
Megabat-ARGFX     .T. ACT ... ... ... C.C ..A ... ..T G.. ... ... ... .G. C.. ... CT. ... ... ..A ... ... ..A ... ... A.. ... ..T ... ...  [ 720]
```

```
Human-ARGFX        GCC TAT GAC ATA TTC CAA ATC ATA GAA CTG TAC AAT CTT CCT GAT GAG AAT GAG ATA TCC AGC TCT TCT TTC CAC TGT CTG TAT CAG TAT  [ 810]
Chimp-ARGFX        ... ... ... ... ... ... ... ... ... ... ... ... ... ... ... ... ... ... ... ... ... ... ... ... ... ... ... ... ... ...  [ 810]
Orangutan-ARGFX    ... ... ... ... ... ... ... ... ... ... ... ... ... ... ... ... ... ... ... ... ... ... ... ... ... ... ... ... ... ...  [ 810]
Macaque-ARGFX      ... ... ... ... ... ... ... ... ... ... ... ... ... ... ... ... ... ... ... ... ... ... ... ... ... ... ... C.. ... ...  [ 810]
Marmoset-ARGFX     ... ... ... ... .C. ... G.. ... ... ... ..T G.. ... ... ... ... ... ... ... ... ... ... ... ... ... ... ... ... ... ...  [ 810]
Mouse_Lemur-ARGFX  T.T ... ... ... GG. ... ... ..G ... G.. ... .G. T.. ... ... ..A G.. ... ... ... --- .G. ..C ... ... ... ..A ... ... ...  [ 810]
Tree_shrew-ARGFX   .T. ... ... ..T GC. ... ... ... ..C ... ... .G. T.. ..C ... ... G.. ... .CG ... ... ... ..C ... ... ... ... ... ... ...  [ 810]
Guinea_Pig-ARGFX   ... .T. ... ... AG. ... ... ... ..C ..A ... .G. T.. ... ... ... TG. ... GAC ..T --- GAC .G. ..T GG. ... ... ... ... ...  [ 810]
Cow-ARGFX          ..A .T. ... ..C AC. ... ... ..G ... ... ... .G. G.. ... ... ... G.. ..C ..C A.. .A. ... ..C ... T.T .C. ..A ... ... ...  [ 810]
Horse-ARGFX        ... ... ... ... GC. ... ... ..G A.. ... ..T .G. T.. ... ... ..A G.. ... ... ... ... ... ... ..T ..G .A. ... ... TG. ...  [ 810]
Megabat-ARGFX      ... ... ... ... AC. ... ... ..G ... .G. ... .G. T.A ... ... ... G.. ... G.. ... --- CAC ... ... T.. A.. ..A ... ... ...  [ 810]

Human-ARGFX        CTC TCA CCC ACA AAG TAC CAG GTA GGA GGA CAG GGT TCC TCT CTC --- AGC ATC TTT GCT GGT CCA GCT GTA GGC CTA TCT CCT GCA CAA  [ 900]
Chimp-ARGFX        ... ... ... ... ... ... ... ... ... ... ... ... ... ... ... --- ... ... ... ... ... ... ... ... ... ... ... ... ... ...  [ 900]
Orangutan-ARGFX    ... ... ... ... ... ... ... ... ... ... ... ... ... ... ... --- ... .C. ... ... ... ... ... ... ... ... ... ..C ... ...  [ 900]
Macaque-ARGFX      ... ... ... ... ... ... ... A.. .A. ... ... ... ... ... ... --- ... .C. ... ... ... ... ... ... ... ... ... ..C ... ...  [ 900]
Marmoset-ARGFX     ... ..G ..A ... ... C.. ... A.. ... ... ... ... ... ... ... --- ... ... ... ... ... ... ... ... ... .G. ... ..C AT. ...  [ 900]
Mouse_Lemur-ARGFX  ... ... ... ... .GT .C. ... C.. ... .A. T.. ... ... ... A.. --- ... .C. C.. ... .TC A.. ... ... .CT ... ... ... AGG ...  [ 900]
Tree_shrew-ARGFX   ... ... ... ... GG. .C. ..A CA. ... .A. ... ... ... ... ..T --C ... CC. ... ..C CA. ... ... A.. AAG ... C.. ..A .GG .C.  [ 900]
Guinea_Pig-ARGFX   ..T ... ..T ... .C. .C. ... CC. .A. .A. A.T .A. GT. ... ... TGC TC. C.T .A. .G. --- ..G .T. A.T T.T .C. C.. ... .GG ...  [ 900]
Cow-ARGFX          ... ... ..G ... .G. C-- .CA ..T A.. --- ATA .AG .-- ... TCT CTT .TA G.. ... ... .A. ... ... ... ... T.. .T. ... .GG ..G  [ 900]
Horse-ARGFX        ... ..G ... ... .G. CC. ... C.G .A. --- G.A CAG AGT ..C TCT CTT ... ... ... ... ... .T. .GA ... ..T ... ..C ... .GG ...  [ 900]
Megabat-ARGFX      ... ... ... ... GGC C.. -G. C.. AA. --- G.A CAA GGT ... --- TTT ... ... ... ... ... ... A.. ... ..T .G. ... ... .GT A..  [ 900]
```

```
Human-ARGFX        ACC --- TGG CCC AAT ATG ACA AGC CAA GCC TTT GAA GCC TAC AGT CTA --- --- ACA GAT AGC CTG GAA TTC CAG AAA ACC TCC AAT ATG  [ 990]
Chimp-ARGFX        ... --- ... ... ... ... ... ... ... .G. ... ... ... ... ... ... --- --- ... ... ... ... ... ... ... ... ... ... ... ...  [ 990]
Orangutan-ARGFX    ... --- ... ... .G. ... ... ... ... .A. ... ... ... ... ... ... --- --- ... ... ... ... ... ... ... ... ... ... ... ...  [ 990]
Macaque-ARGFX      ... --- ... ... .G. ... ... ... ... .G. .G. C.. ... ... ... .A. gc- -CA ... ... ... ... ... ..T ... ... ... ... ... ...  [ 990]
Marmoset-ARGFX     ... --- ... ... .G. ... ... ... ... .G. ... ... ... ... ... .C. --- --- ... ..C ..T ..A ... ... T.. ... ... ... ... ...  [ 990]
Mouse_Lemur-ARGFX  G.. --- ... T.. .G. .G. ..G ... ... AG. ... ... TAT ..A ... ... --- --- .G. ..A ... ..T ... ... ... ..T ..T ... ... ...  [ 990]
Tree_shrew-ARGFX   ... --- ..A T.. .G. G.. .TG G.. ... AAT ... .C. T.T .G. .AC .-. --- --- .G. ..C ..T ..A ... ... ... .GC ... ... .G. ...  [ 990]
Guinea_Pig-ARGFX   ... TGC ATA ... .G. G.. .GG ... ... C.. .G. .CT ... .GG G.. .C- --- --- -.C AG. ... ... .-- C.. ... ..C C.. ... .G. .-.  [ 990]
Cow-ARGFX          ... --- .CC TT. .G. G.. ... ... TGG AG. ... .C. .T. ... ... .C. --- --- CAG ..C ... ... ... ... ... ..C C.. ... .T. .CA  [ 990]
Horse-ARGFX        ... --- ... T.. .G. ... ... ... ..G CAT ... .C. AT. A.. ... ... --- --- .G. .GG GTT ..A ... ... ... ..C C.T ... .G. ...  [ 990]
Megabat-ARGFX      T.T --- CAT ..A -G. ... ... ... TG. .G. C.. .C. ... ..T ... ... --- --- .G. ..C ... ... ... ... ... ..C CT. ... ... ...  [ 990]

Human-ARGFX        GTA GAC TTG GGA TTT CTC TGA ---  [1014]
Chimp-ARGFX        ... ... ... ... ... ... ... ---  [1014]
Orangutan-ARGFX    ... ... ... ... ... ... .C. ---  [1014]
Macaque-ARGFX      ... ... ... ... C.A GAG .AC TAA  [1014]
Marmoset-ARGFX     ... .C. ..T ... ..A ... ... ---  [1014]
Mouse_Lemur-ARGFX  ... A.. ..T ... ..A ... ... ---  [1014]
Tree_shrew-ARGFX   ... ... ... .-. ... ... ... ---  [1014]
Guinea_Pig-ARGFX   .C. ... .GT ATT .C. AAT G.. ---  [1014]
Cow-ARGFX          ..G C.. ..T ... ... ... ... ---  [1014]
Horse-ARGFX        ..G ... ..T ... G.. ..T ... ---  [1014]
Megabat-ARGFX      A.G .G. ..T ... ..C .C. ... ---  [1014]
```
