# Supplementary material for: The origin and evolution of ARGFX homeobox loci in mammalian radiation
Source: BMC Evol Biol. 2010 Jun 17;10:182. doi: 10.1186/1471-2148-10-182 (PMC2894831; doi:10.1186/1471-2148-10-182)
Supplement: Additional file 3 — Pairwise poisson-distances between ten deduced ARGFX protein sequences. [file 1471-2148-10-182-S3.DOC]

**Additional file 3**

*Pairwise poisson-distances between ten deduced ARGFX protein sequences*.

| Species | N-terminal (30 aa) | Homeobox region (59 aa) | C-terminal (149 aa) | N-terminal *minus* Homeobox region | C-terminal *minus* Homeobox region |
| --- | --- | --- | --- | --- | --- |
| Human Vs Chimpanzee | 0.069 | 0.000 | 0.007 | 0.069 | 0.007 |
| Human Vs Orangutan | 0.069 | 0.034 | 0.027 | 0.035 | -0.007 |
| Human Vs Macaque | 0.266 | 0.107 | 0.091 | 0.159 | -0.016 |
| Human Vs Marmoset | 0.405 | 0.166 | 0.176 | 0.239 | 0.010 |
| Human Vs Tree Shrew | 0.629 | 0.249 | 0.389 | 0.380 | 0.140 |
| Human Vs Guinea Pig | 0.916 | 0.364 | 0.799 | 0.552 | 0.435 |
| Human Vs Cow | 0.916 | 0.249 | 0.504 | 0.667 | 0.255 |
| Human Vs Horse | 0.568 | 0.293 | 0.450 | 0.275 | 0.157 |
| Human Vs Megabat | 1.455 | 0.414 | 0.419 | 1.041 | 0.005 |
| Chimpanzee Vs Orangutan | 0.143 | 0.034 | 0.027 | 0.109 | -0.007 |
| Chimpanzee Vs Macaque | 0.357 | 0.107 | 0.084 | 0.250 | -0.023 |
| Chimpanzee Vs Marmoset | 0.511 | 0.166 | 0.168 | 0.345 | 0.002 |
| Chimpanzee Vs Tree Shrew | 0.629 | 0.249 | 0.389 | 0.380 | 0.140 |
| Chimpanzee Vs Guinea Pig | 0.836 | 0.364 | 0.799 | 0.472 | 0.435 |
| Chimpanzee Vs Cow | 0.916 | 0.249 | 0.504 | 0.667 | 0.255 |
| Chimpanzee Vs Horse | 0.629 | 0.293 | 0.450 | 0.336 | 0.157 |
| Chimpanzee Vs Megabat | 1.322 | 0.414 | 0.409 | 0.908 | -0.005 |
| Orangutan Vs Macaque | 0.266 | 0.107 | 0.084 | 0.159 | -0.023 |
| Orangutan Vs Marmoset | 0.405 | 0.166 | 0.184 | 0.239 | 0.018 |
| Orangutan Vs Tree Shrew | 0.629 | 0.249 | 0.399 | 0.380 | 0.150 |
| Orangutan Vs Guinea Pig | 0.916 | 0.364 | 0.814 | 0.552 | 0.450 |
| Orangutan Vs Cow | 0.836 | 0.271 | 0.504 | 0.565 | 0.233 |
| Orangutan Vs Horse | 0.568 | 0.293 | 0.471 | 0.275 | 0.178 |
| Orangutan Vs Megabat | 1.322 | 0.440 | 0.429 | 0.882 | -0.011 |
| Macaque Vs Marmoset | 0.457 | 0.126 | 0.208 | 0.331 | 0.082 |
| Macaque Vs Tree Shrew | 0.629 | 0.227 | 0.461 | 0.402 | 0.234 |
| Macaque Vs Guinea Pig | 1.322 | 0.364 | 0.784 | 0.958 | 0.420 |
| Macaque Vs Cow | 1.099 | 0.271 | 0.573 | 0.828 | 0.302 |
| Macaque Vs Horse | 0.511 | 0.293 | 0.527 | 0.218 | 0.234 |
| Macaque Vs Megabat | 1.322 | 0.414 | 0.461 | 0.908 | 0.047 |
| Marmoset Vs Tree Shrew | 0.511 | 0.249 | 0.482 | 0.262 | 0.233 |
| Marmoset Vs Guinea Pig | 1.099 | 0.364 | 0.830 | 0.735 | 0.466 |
| Marmoset Vs Cow | 1.099 | 0.293 | 0.561 | 0.806 | 0.268 |
| Marmoset Vs Horse | 0.629 | 0.340 | 0.504 | 0.289 | 0.164 |
| Marmoset Vs Megabat | 1.322 | 0.467 | 0.419 | 0.855 | -0.048 |
| Tree Shrew Vs Guinea Pig | 1.322 | 0.414 | 0.770 | 0.908 | 0.356 |
| Tree Shrew Vs Cow | 1.455 | 0.249 | 0.609 | 1.206 | 0.360 |
| Tree Shrew Vs Horse | 0.836 | 0.249 | 0.527 | 0.587 | 0.278 |
| Tree Shrew Vs Megabat | 1.099 | 0.364 | 0.573 | 0.735 | 0.209 |
| Guinea Pig Vs Cow | 1.099 | 0.364 | 0.893 | 0.735 | 0.529 |
| Guinea Pig Vs Horse | 1.322 | 0.440 | 0.784 | 0.882 | 0.344 |
| Guinea Pig Vs Megabat | 2.015 | 0.522 | 0.861 | 1.493 | 0.339 |
| Cow Vs Horse | 0.916 | 0.316 | 0.450 | 0.600 | 0.134 |
| Cow Vs Megabat | 1.455 | 0.389 | 0.461 | 1.066 | 0.072 |
| Horse Vs Megabat | 1.204 | 0.227 | 0.429 | 0.977 | 0.202 |
| Mean distance | 0.851 | 0.280 | 0.461 | 0.571 | 0.181 |
